# Diet quality indices and dietary patterns are associated with plasma metabolites in colorectal cancer patients

**DOI:** 10.1007/s00394-021-02488-1

**Published:** 2021-02-05

**Authors:** Anne J. M. R. Geijsen, Dieuwertje E. Kok, Moniek van Zutphen, Pekka Keski-Rahkonen, David Achaintre, Audrey Gicquiau, Andrea Gsur, Flip M. Kruyt, Cornelia M. Ulrich, Matty P. Weijenberg, Johannes H. W . de Wilt, Evertine Wesselink, Augustin Scalbert, Ellen Kampman, Fränzel J. B. van Duijnhoven

**Affiliations:** 1 Division of Human Nutrition and Health, Wageningen University and Research, P.O. Box 17, 6700 AA Wageningen, The Netherlands; 2 Biomarker Group, International Agency for Research on Cancer, Lyon, France; 3 Institute of Cancer Research, Department of Medicine I, Medical University of Vienna, Vienna, Austria; 4 Department of Surgery, Hospital Gelderse Vallei, Ede, The Netherlands; 5 Huntsman Cancer Institute, Salt Lake City, Utah USA; 6 Department of Population Health Sciences, University of Utah, Salt Lake City, Utah USA; 7 Department of Epidemiology, GROW School for Oncology and Developmental Biology, Maastricht University, Maastricht, The Netherlands; 8 Department of Surgery, Division of Surgical Oncology and Gastrointestinal Surgery, Radboud University Medical Centre, Nijmegen, The Netherlands

**Keywords:** Colorectal cancer patients, Diet quality indices, Dietary patterns, Metabolites, Metabolomics

## Abstract

**Purpose:**

Emerging evidence suggests that diet is linked to survival in colorectal cancer patients, although underlying mechanisms are not fully understood. The aim of this study was to evaluate whether dietary exposures are associated with metabolite concentrations in colorectal cancer patients.

**Methods:**

Concentrations of 134 metabolites of the Biocrates Absolute^IDQ^ p180 kit were quantified in plasma samples collected at diagnosis from 195 stage I-IV colorectal cancer patients. Food frequency questionnaires were used to calculate adherence to the World Cancer Research Fund (WCRF) dietary recommendations and the Dutch Healthy Diet (DHD15) index as well as to construct dietary patterns using Principal Component Analysis. Multivariable linear regression models were used to determine associations between dietary exposures and metabolite concentrations. All models were adjusted for age, sex, body mass index, smoking status, analytical batch, cancer stage, and multiple testing using false discovery rate.

**Results:**

Participants had a mean (SD) age of 66 (9) years, were mostly men (60%), and mostly diagnosed with stage II and III cancer. For the dietary pattern analyses, Western, Carnivore, and Prudent patterns were identified. Better adherence to the WCRF dietary recommendations was associated with lower concentrations of ten phosphatidylcholines. Higher intake of the Carnivore pattern was associated with higher concentrations of two phosphatidylcholines. The DHD15-index, Western pattern, or Prudent pattern were not associated with metabolite concentrations.

**Conclusion:**

In the current study, the WCRF dietary score and the Carnivore pattern are associated with phosphatidylcholines. Future research should elucidate the potential relevance of phosphatidylcholine metabolism in the colorectal cancer continuum.

**Clinical trial registry:**

ClinicalTrials.gov Identifier: NCT03191110.

**Supplementary Information:**

The online version contains supplementary material available at 10.1007/s00394-021-02488-1.

## Introduction

Previous research suggests that there is a relation between diet and survival after colorectal cancer diagnosis [1]. However, the underlying mechanisms are largely unknown. The identification of metabolites associated with diet in colorectal cancer patients could be a first step in unravelling the link between dietary exposures and colorectal cancer progression [2–4] and understanding the biological processes involved [5, 6]. Metabolomics measures a range of small molecules, many of which belong to a number of different biochemical pathways. Such metabolic profiles can provide a snapshot of the current metabolic state of the body, characteristic of a phenotype [7] and are, therefore, increasingly used to study the interface of diet, lifestyle and diseases [8–10].

Thus far, research has shown that dietary exposures can be associated with metabolite concentrations in blood. Predefined diet quality indices [11] as well as distinct dietary patterns such as veganism [12, 13] were reported to be associated with specific blood metabolites. Three diet quality indices were associated with metabolites, including mainly lipids and amino acids [11]. Another study reported that a vegan diet was associated with lower concentrations of glycerophospholipids, sphingolipids and amino acids compared to a diet containing meat and/or fish [12].

In terms of understanding the complex relationship between diet and metabolites, investigating dietary patterns and indices, instead of single nutrients or food groups, is of specific interest since nutrients and food groups may interact with each other [14]. Diet quality indices and dietary patterns are used to assess exposure to combinations of food groups. An example of a diet quality index, commonly used to investigate the relationship with health outcomes after cancer diagnosis [15–17], is the World Cancer Research Fund (WCRF) score that is based on the 2018 cancer prevention recommendations of the WCRF/American Institute for Cancer Research (AICR) [18]. The Dutch Healthy Diet index (DHD15-index) is used to assess the adherence to the Dutch dietary guidelines of 2015 [19] and has also previously been associated with various health outcomes [20, 21]. In addition to these indices, dietary patterns are data-driven and are also commonly evaluated against health outcomes after cancer diagnosis [22].

It is well-established that disease can have a great impact on metabolism [23–25] and, to the best of our knowledge, no research has been conducted investigating the associations between dietary exposures and metabolites in cancer patients. Metabolites associated with dietary exposures in colorectal cancer patients may give clues to potential underlying mechanisms for colorectal cancer progression which could be studied in detail in the future. Therefore, the aim of this explorative study was to investigate whether the diet, evaluated using diet quality indices and dietary patterns, is associated with plasma metabolites in colorectal cancer patients.

## Methods

### Study population

In total, 200 stage I-IV colorectal cancer patients with available plasma metabolite concentrations of the COLON study [26], a prospective cohort study among colorectal cancer patients in the Netherlands, were considered for the present study. The design and recruitment of the COLON study has been described earlier [26]. Participants were recruited from 11 hospitals in the Netherlands, shortly after colorectal cancer diagnosis. Females and males of all ages and of any stage of the disease were eligible. Non-Dutch speaking patients, patients with a history of colorectal cancer or (partial) bowel resection, chronic inflammatory bowel disease, hereditary colorectal cancer syndromes (e.g. Lynch syndrome, Familial Adenomatous Polyposis, Peutz-Jegher), dementia or another mental condition causing an inability to fill out a questionnaire correctly were excluded. All participants provided a written informed consent. The COLON study was approved by the Committee on Research involving Human Subjects (region Arnhem-Nijmegen), the Netherlands.

Participants with missing dietary intake data (*n* = 2) or with a missing cancer stage (*n* = 3) were excluded from the current study, resulting in a final study population of *n* = 195 stage I-IV colorectal cancer patients for analysis.

### Data collection

Habitual dietary intake in the month prior to diagnosis was assessed using a 204-item validated, semi-quantitative food frequency questionnaire (FFQ) developed by the Division of Human Nutrition and Health of Wageningen University & Research, the Netherlands [27, 28]. The FFQ was used to calculate a priori defined diet quality indices and to construct a posteriori data-driven dietary patterns. Demographic and lifestyle characteristics such as sex, age, weight, height, and smoking habits were assessed using self-administered questionnaires. All questionnaires were filled out prior to tumor resection. Medical information, including cancer stage, tumor location, and treatment strategies, was collected using the Dutch ColoRectal Audit [29].

Non-fasted plasma EDTA samples were collected upon recruitment, which were intended before the start of treatment, and stored at − 80 °C using a standardized protocol to ensure identical sample handling across the eleven hospitals.

### Diet quality indices

Two diet quality indices have been included in the current study, namely the WCRF dietary score and the DHD15-index. Briefly, the WCRF dietary score is based on the 2018 WCRF/AICR recommendations for cancer prevention using the standard WCRF/AICR score developed by Shams-White et al. [18]. Since the current study focusses on dietary intake, the recommendations regarding weight, physical activity, supplement use, and breastfeeding were not included. The remaining recommendations were: (1) eat a diet rich in whole grains, vegetables, fruits, and beans, (2) limit consumption of ‘fast foods’ and other processed foods high in fat, starches or sugar, (3) limit consumption of red and processed meat, (4) limit consumption of sugar-sweetened drinks, and (5) reduce alcohol consumption. Quantitative criteria were used as cut-off points for all recommendations, except for the recommendation (2) limit consumption of ‘fast foods’ and other processed foods high in fat, starches or sugar, where cut-offs were based on tertiles calculated as a percentage of total energy intake from processed foods. Processed foods included French fries, crisps, pastry and biscuits, savory snacks, sugar and candy, sauces, pizza, pancake, sandwich fillings high in sugar or fat, refined grain products, and sweet dairy desserts. Processed meat included sausages, bacon, ribs, ham, cold cuts, and unknown types of meat. Sugary drinks included sugar-sweetened soft drinks, sugar-sweetened dairy drinks, and fruit juices. We did not include yoghurt and cheese, nuts, oils and fats, and diet soft drinks in the WCRF dietary score, since these food or food groups are not part of the WCRF recommendations.

The score assigned for each recommendation of the WCRF dietary score was 1 when the recommendation was met (full adherence), a score of 0.5 was assigned to moderate adherence and a score of 0 was assigned to low adherence. The recommendation regarding a diet rich in whole grains, vegetables, fruit, and beans, included sub-recommendations for fiber intake and for fruit and vegetable consumption. As a result, the recommendation score was the sum of sub-recommendation scores of fiber intake and fruit and vegetables intake, meaning that plausible scores were 0, 0.25, 0.5, 0.75, and 1. The overall score of the WCRF dietary score ranged from 0 to 5.

The DHD15-index [19] was developed on the basis of the 2015 Dutch dietary guidelines [30] and refers to 15 recommendations. In the current study, the recommendations regarding coffee consumption and sodium intake were excluded since the type of coffee and sodium intake were not assessed in the COLON study. The DHD15-index used in the current study included the intake of sugary drinks, liquid fats and oils, processed meat, red meat, nuts, dairy products, refined grains products, whole grain products, vegetables, alcohol, legumes, solid fat, fruit, fish, and tea. The scores for each individual recommendation ranged from 0 to 10 points with a maximum total DHD15-index score of 130 points.

For both indices, a higher score represents a healthier diet, i.e. a better compliance with the recommendations of the corresponding diet quality index. Details on the used diet quality indices have been described before [18, 19, 31].

### Empirical construction of dietary patterns

Total intake of food items (g/d) and total energy intake (kcal/d) were calculated based on frequency of intake, number of portions, portion size, and the type of products, as recorded in the FFQ. All food items were categorized into 33 food groups that were constructed according to the Dutch food composition table 2011 [32]. Final food groups are described in Supplementary Table S1. Total intake of food groups was recalculated to relative intake (g/d per 1000 kcal) using total energy intake to simplify comparison of participants.

Principal component analysis was used to investigate data-driven dietary patterns among participants [33]. Food group data were log-transformed using the natural logarithm and Z-standardized before performing principal component analysis. As a result, the intake of all food groups has a mean of zero and a variance of one, which is important since the results of components highly depend on the variance of each variable [34]. In case a certain food group was not consumed, i.e. 0 g/day per 1000 kcal, 0.001 was added to the food group sum to allow log transformation. The number of dietary patterns was decided based on the components with eigenvalues > 1.0, the scree plot and the interpretability of the components [35]. The remaining components were orthogonally rotated for ease of interpretation and labels in accordance with the included food groups were given. Positive and negative food group loadings >|0.2| were considered when naming the respective dietary pattern. Participants’ scores were determined by multiplying the observed intake of all food groups by the factor loading for each of all the respective food groups [36].

**Fig. 1:**
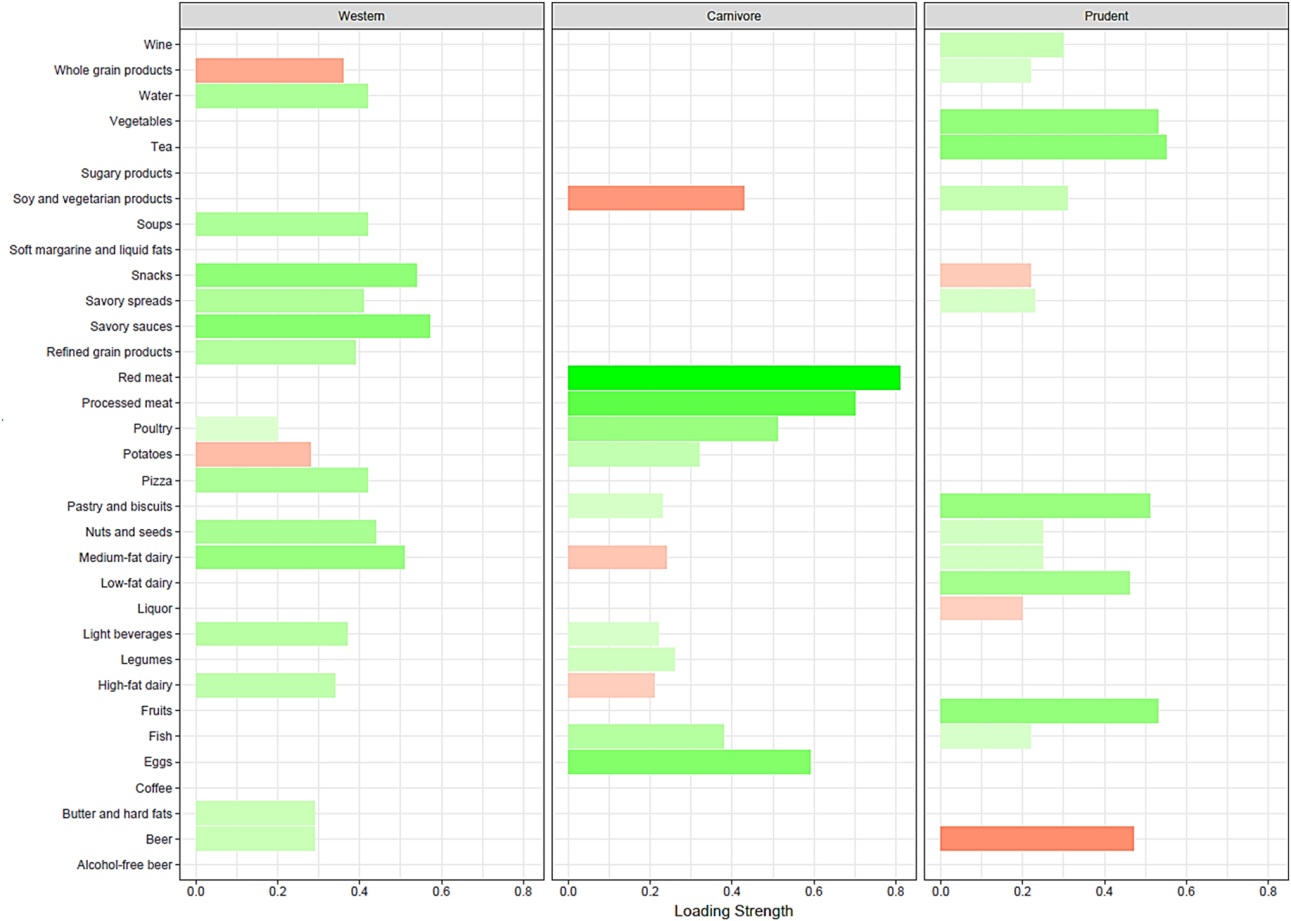
Overview of food group loadings of the Western, Carnivore, and Prudent dietary patterns. Green and red bars represent positive and negative loading strengths, respectively. A more positive loading illustrates higher consumption of a specific food group, while a more negative loading characterizes lower consumption of the food group. Only food group loadings >|0.2| were considered to contribute to the dietary pattern and visualized to improve readability

Three dietary patterns were identified based on the available data, which were defined as a Western, Carnivore, and Prudent dietary pattern, see Fig. 1. The Western dietary pattern was characterized by a high intake of snacks, savory sauces and spreads, refined grains, pizza, high and medium-fat dairy, nuts and seeds, beer, and hard fats, and a low intake of whole grain products and potatoes. The Carnivore pattern was characterized by a high intake of red and processed meat, poultry, fish, eggs, and potatoes, and a low intake of soy and vegetarian products, and medium and high-fat dairy. Lastly, the Prudent pattern consisted of a high intake of vegetables, fruits, fish, nuts and seeds, low-fat dairy, tea, pastry and biscuits, and a low intake of beer.

### Biomarker analysis

Plasma samples were analyzed in four analytical batches at the International Agency for Research on Cancer (IARC) in Lyon, France. In total, 147 metabolites were measured using the Absolute^IDQ^ p180 kit (Biocrates, Innsbruck, Austria). The analytical method [37, 38] characterizes up to 188 metabolites from five compound classes. Amino acids and biogenic amines were quantified (calibration curves, individual isotope-labelled internal standards) by ultra-high performance liquid chromatography-tandem mass spectrometry (UPLC-MS/MS). Lipids, including glycerophospholipids and sphingolipids, acylcarnitines, as well as the sum of hexose sugars were semi-quantified (one-point calibration, single representative internal standard) by flow injection analysis-tandem mass spectrometry (FIA-MS/MS).

Metabolites with > 20% missing values (*n* = 13), including values below the level of detection (LOD) and true missings, were removed from the dataset while remaining missing values were imputed in line with previous studies [38–40]. Briefly, missing values below the LOD were imputed by half of the batch-specific LOD and values below or above the quantitative range were replaced by the lower or upper limit of quantification, respectively. Subsequently, to normalize distributions, metabolite concentrations were log transformed using the natural logarithm and were Z-standardized to allow comparison of estimates among metabolites. In total, 134 metabolites were included in the current analysis (Supplementary Table S2), consisting of 12 acylcarnitines (Cx:y), 21 amino acids, 8 biogenic amines, 78 glycerophospholipids (10 lysophosphatidylcholines (lysoPC) and 68 phosphatidylcholines [PC diacyl (aa) and acyl-alkyl (ae)], 14 sphingolipids (SM Cx:y) and the sum of hexoses. The abbreviation Cx:y is used to describe the total number of carbons and double bonds in the alkyl chains, respectively.

### Statistical analysis

Clinical, demographic and lifestyle characteristics were described using descriptive analyses. Linear regression models were used to determine the associations between diet quality indices and dietary patterns as independent variables and concentrations of metabolites as dependent variables. Diet quality indices and dietary patterns were analyzed as continuous variables as well as analyzed in tertiles, for which the lowest tertile, corresponding to the lowest intake of the exposure, was used as the reference category. *P*-trend values were computed for tertiles using the medians of the corresponding tertiles.

All models were adjusted for age at diagnosis (continuous), sex, BMI (continuous), smoking status (current smoker/former smoker/never smoker), analytical batch (1–4), and cancer stage (stage I/stage II/ stage III/ stage IV). The basis for assessing whether covariates should be included in the final model were existing evidence, biological plausibility and whether the regression coefficient of interest changed by > 10% after adding the potential covariate.

Furthermore, to explore the consistency in the observed associations between dietary exposures and plasma metabolites, we evaluated the top-15 metabolites (based on the smallest *p*-value for trend over tertiles) associated with diet quality indices and dietary patterns using a heatmap.

Sensitivity analyses were conducted excluding patients from whom blood was collected during or after any type of treatment, i.e. (neo-) adjuvant chemotherapy and/or surgery (*n* = 19) and excluding stage IV patients (*n* = 7).

All statistical analyses were performed in R, version 3.4.0 and SAS, version 9.4. After correction for multiple testing, using false discovery rate (FDR) according to the Benjamin-Hochberg procedure [41, 42], a *p *value (*p*_FDR_) < 0.05 was considered statistically significant.

## Results

### Study population characteristics

Characteristics of the overall study population are summarized in Table 1. The mean (SD) age of the 195 colorectal cancer patients was 66 (9) years and almost 60% of the study population was male. Mean (SD) BMI was 25.6 (4.9) kg/m^2^, around ten percent were current smokers and participants had a stage I (27%), stage II (33%), stage III (36%) or stage IV (4%) cancer. Distal colon cancer was diagnosed in 36% of the study population, proximal colon cancer and rectal cancer in 29% and 35%, respectively. The study population had a mean (SD) total energy intake of 1856 (559) kcal/day. The mean (SD) WCRF dietary score and DHD15-index were 2.1 (0.7) and 73.8 (14.1), respectively. There were no consistent different directions in diet quality indices and dietary patterns when comparing colorectal cancer stages (data not shown). Baseline characteristics of the participants in the lowest and highest tertile of each diet quality index and dietary pattern are shown in Supplementary Table S3.

**Table 1:** Baseline characteristics of the overall study population

	Study population
Number of participants	195
Male sex, *n* (%)	115 (59)
Age	66.2 ± 9.1
Body mass index (kg/m^2^)	25.6 ± 4.9
Underweight, <18.5, *n* (%)	3 (2)
Normal weight, 18.5-24.9, *n* (%)	85 (44)
Overweight, 25-29.9, *n* (%)	79 (41)
Obese, ≥30, *n* (%)	28 (14)
Smoking, *n* (%)	
Current	19 (10)
Former	118 (60)
Never	58 (30)
Stage, *n* (%)	
I	53 (27)
II	65 (33)
III	70 (36)
IV	7 (4)
Tumor site^a^, *n* (%)	
Colon—proximal	56 (29)
Colon—distal	71 (36)
Rectal	68 (35)
Treatment, *n* (%)	
Surgery	194 (99)
Neo-adjuvant treatment	60 (31)
Total energy intake (kcal/day)	1856 ± 559
Total WCRF dietary score^b^	2.1 ± 0.7
Total DHD15-index^c^	73.8 ± 14.1

Numbers are presented as mean ± SD or median (IQR) unless mentioned otherwise

^a^Proximal consisting of: hepatic flexure, transverse colon, cecum, appendix, ascending colon; distal consisting of: descending colon, sigmoid colon, splenic flexure; rectal consisting of: rectosigmoid junction, rectum

^b^Adherence to the dietary recommendations of the World Cancer Research Fund (WCRF), ranged 0 to 5 [18]

^c^Adherence to the Dutch Healthy Diet guidelines 2015 (DHD15), ranged 0 to 130 [19]

### Diet quality indices

Table 2 presents the top-15 metabolites based on the smallest value of *p*_trend_ across tertiles for the analyses between diet quality indices and metabolite concentrations, which were ranked by the *p*_trend_ across tertiles of the diet quality indices. A higher concordance of the WCRF dietary score was statistically significantly associated after FDR adjustment with lower concentrations of ten phosphatidylcholines over increasing tertiles. Each one-point increase in the WCRF dietary score also showed statistically significantly lower concentrations of four of the above-mentioned ten phosphatidylcholines (PC ae C36:3, PC ae C36:4, PC aa C36:3, and PC aa C38:3).

The DHD15-index was not statistically significantly associated with plasma metabolites after FDR adjustment when analyzed by tertiles of the DHD15-index and continuously (Table 2). An overview of all the results on the association between the diet quality indices and all 134 metabolites is available in Supplementary Table S2.

**Table 2:** Top-15 plasma metabolites associated with diet quality indices, ranked by *p*_trend_

		Continuous			Tertiles								
					1		2			3			
Metabolite		β	95% CI	*p*_FDR_^c^			β	95% CI	*p*_FDR_^c^	β	95% CI	*p*_FDR_^c^	*p*_trend_^c^
Diet quality index: WCRF dietary score^a,b^													
No. of participants		195			77		56			62			
Mean score (range)		2.1 (0.50–4.25)			1.40 (0.50–1.75)		2.10 (2.00–2.25)			2.80 (2.50–4.25)			
*1*	PC ae C36:3	− 0.38	(− 0.58;− 0.19)	**0.02**	Ref		− 0.37	(− 0.69;− 0.06)	0.39	− 0.67	(− 0.97;− 0.36)	**0.003**	**0.005**
*2*	PC ae C36:4	− 0.37	(− 0.58;− 0.16)	**0.03**	Ref		− 0.29	(− 0.63;0.04)	0.49	− 0.67	(− 0.99;− 0.35)	**0.004**	**0.006**
*3*	PC aa C36:3	− 0.34	(− 0.54;− 0.14)	**0.04**	Ref		− 0.08	(− 0.40;0.24)	0.91	− 0.59	(− 0.90;− 0.28)	**0.01**	**0.01**
*4*	PC aa C38:3	− 0.34	(− 0.53;− 0.15)	**0.03**	Ref		− 0.11	(− 0.42;0.20)	0.87	− 0.53	(− 0.82;− 0.23)	**0.01**	**0.02**
*5*	PC ae C34:2	− 0.30	(− 0.50;− 0.10)	0.08	Ref		− 0.34	(− 0.66;− 0.01)	0.47	− 0.55	(− 0.86;− 0.24)	**0.01**	**0.02**
*6*	PC ae C38:4	− 0.29	(− 0.49;− 0.09)	0.09	Ref		− 0.24	(− 0.57;0.08)	0.61	−0.56	(− 0.87;− 0.25)	**0.01**	**0.02**
*7*	PC aa C40:4	− 0.30	(− 0.51;− 0.09)	0.09	Ref		− 0.26	(− 0.60;0.08)	0.60	− 0.52	(− 0.85;− 0.20)	**0.03**	**0.04**
*8*	PC ae C38:3	− 0.27	(− 0.46;− 0.08)	0.09	Ref		− 0.05	(− 0.36;0.26)	0.92	− 0.48	(− 0.77;− 0.18)	**0.03**	**0.04**
*9*	PC ae C38:5	− 0.30	(− 0.50;− 0.10)	0.08	Ref		− 0.22	(− 0.55;0.11)	0.71	− 0.52	(− 0.83;− 0.21)	**0.03**	**0.04**
*10*	PC ae C40:4	− 0.24	(− 0.44;− 0.04)	0.15	Ref		− 0.32	(− 0.64;0.01)	0.49	− 0.50	(− 0.80;− 0.19)	**0.03**	**0.04**
*11*	PC aa C34:2	− 0.25	(− 0.47;− 0.04)	0.15	Ref		− 0.12	(− 0.47;0.23)	0.87	− 0.46	(− 0.80;− 0.13)	0.08	0.07
*12*	PC aa C32:1	− 0.26	(− 0.47;− 0.05)	0.15	Ref		− 0.16	(− 0.51;0.19)	0.82	− 0.44	(− 0.78;− 0.11)	0.09	0.09
*13*	PC aa C34:1	− 0.27	(− 0.48;− 0.06)	0.14	Ref		− 0.12	(− 0.46;0.23)	0.87	− 0.44	(− 0.77;− 0.10)	0.09	0.09
*14*	PC ae C32:1	− 0.24	(− 0.45;− 0.04)	0.15	Ref		− 0.29	(− 0.63;0.04)	0.49	− 0.44	(− 0.76;− 0.12)	0.08	0.09
*15*	PC ae C34:1	− 0.25	(− 0.45;− 0.06)	0.14	Ref		− 0.19	(− 0.51;0.13)	0.74	− 0.41	(− 0.72;− 0.11)	0.08	0.09
Diet quality index: DHD15-index^a,d^													
No. of participants		195			65		65			65			
Mean score (range)		73.8 (40.9–114.8)			58.6 (40.9–68.3)		73.5 (68.4–78.9)			89.2 (78.9–113.8)			
*1*	PC aa C40:4	− 0.17	(− 0.28;− 0.07)	0.15	Ref		− 0.40	(− 0.75;− 0.04)	0.30	− 0.50	(− 0.85;− 0.14)	0.29	0.37
*2*	PC aa C42:0	0.14	(0.04;0.24)	0.20	Ref		0.11	(− 0.22;0.44)	0.85	0.43	(0.09;0.76)	0.45	0.37
*3*	PC aa C42:1	0.13	(0.04;0.23)	0.20	Ref		0.26	(− 0.07;0.59)	0.49	0.47	(0.14;0.81)	0.29	0.37
*4*	PC ae C36:4	− 0.14	(− 0.24;− 0.03)	0.20	Ref		− 0.59	(− 0.94;− 0.24)	0.15	− 0.50	(− 0.85;− 0.15)	0.29	0.37
*5*	lysoPC a C17:0	0.05	(− 0.06;0.16)	0.72	Ref		0.46	(0.11;0.81)	0.21	0.35	(0.00;0.71)	0.54	0.45
*6*	PC aa C32:1	− 0.15	(− 0.25;− 0.04)	0.20	Ref		− 0.40	(− 0.76;− 0.03)	0.33	− 0.39	(− 0.75;− .02)	0.54	0.45
*7*	PC aa C38:6	0.10	(0.00;0.21)	0.36	Ref		− 0.07	(− 0.42;0.27)	0.88	0.35	(0.00;0.70)	0.54	0.45
*8*	PC aa C40:5	− 0.14	(− 0.24;− 0.03)	0.20	Ref		− 0.29	(− 0.64;0.06)	0.48	− 0.38	(− 0.73;− 0.03)	0.54	0.45
*9*	PC ae C38:5	− 0.11	(− 0.21;− 0.01)	0.29	Ref		− 0.47	(− 0.81;− 0.13)	0.21	− 0.40	(− 0.74;− 0.06)	0.54	0.45
*10*	PC ae C40:6	0.10	(0.00;0.20)	0.36	Ref		0.04	(− 0.29;0.38)	0.90	0.32	(− 0.02;0.66)	0.54	0.45
*11*	PC ae C42:3	0.09	(− 0.01;0.19)	0.42	Ref		− 0.02	(− 0.36;0.31)	0.96	0.35	(0.02;0.69)	0.54	0.45
*12*	PC ae C44:6	0.10	(0.00;0.20)	0.36	Ref		0.07	(− 0.27;0.41)	0.88	0.32	(− 0.02;0.66)	0.54	0.45
*13*	Sarcosine	− 0.10	(− 0.21;0.00)	0.36	Ref		− 0.05	(− 0.41;0.31)	0.90	− 0.37	(− 0.73;− 0.01)	0.54	0.45
*14*	SM (OH) C22:2	0.05	(− 0.04;0.14)	0.64	Ref		0.18	(− 0.13;0.49)	0.56	0.28	(− 0.03;0.59)	0.56	0.45
*15*	C14:1	− 0.12	(− 0.23;− 0.02)	0.26	Ref		− 0.19	(− 0.55;0.16)	0.61	− 0.35	(− 0.70;0.01)	0.54	0.45

^a^Tested using multiple linear regression models analyzing associations of diet quality index (continuous and in tertiles) as independent variable and log transformed Z-standardized metabolite concentrations as dependent variable. The continuous analysis is presented per one-point and ten-points increase for the WCRF dietary score and DHD15-index, respectively. Tertile cut-off scores were 1.75 and 2.25 for the WCRF dietary score and 68.3–78.9 for the DHD15-index. Regression models were adjusted for sex, age, analytical batch, body mass index (continuous), smoking status, and stage

^b^Adherence to the recommendations of the World Cancer Research Fund (WCRF), ranged 0 to 5

^c^*p *value adjusted for multiple testing, using false discovery rate (FDR)

^d^Adherence to the Dutch Healthy Diet guidelines 2015 (DHD15), ranged 0 to 130

### Dietary patterns

The top-15 metabolites resulting from analysis of associations between the Western, Carnivore, and Prudent pattern and plasma metabolites, ranked by the *p*_trend_ across tertiles of the dietary patterns, are shown in Table 3. No linear trend was observed over increasing tertiles of the Western pattern in relation to plasma metabolites. In contrast, every SD increase in consumption of the Western pattern was statistically significantly associated with 35 metabolites (Table 3 and Supplementary Table S2).

**Table 3:** Top-15 plasma metabolites associated with dietary patterns, ranked by *p*_trend_

		Continuous			Tertiles								
					1		2			3			
Metabolite		β	95% CI	*p*_FDR_^b,c^			β	95% CI	*p*_FDR_^b^	β	95% CI	*p*_FDR_^b^	*p*_trend_^b^
Dietary pattern: western^a^													
No. of participants		195			65		65			65			
Mean score (range)		0.0 (− 5.2–2.4)			− 1.0 (− 5.2 to − 0.3)		0.1 (− 0.2–0.5)			0.9 (0.5–2.4)			
*1*	PC aa C28:1	0.31	(0.18;0.44)	**0.001**	Ref		0.18	(− 0.14;0.50)	0.97	0.59	(0.26;0.92)	0.08	0.10
*2*	PC aa C36:1	0.24	(0.10;0.38)	**0.01**	Ref		0.24	(− 0.08;0.57)	0.97	0.54	(0.20;0.89)	0.09	0.10
*3*	PC ae C30:0	0.20	(0.06;0.34)	**0.03**	Ref		0.27	(− 0.07;0.60)	0.97	0.49	(0.15;0.84)	0.09	0.10
*4*	PC ae C34:0	0.24	(0.10;0.39)	**0.01**	Ref		0.24	(− 0.09;0.58)	0.97	0.50	(0.15;0.85)	0.09	0.10
*5*	PC ae C36:1	0.25	(0.12;0.39)	**0.01**	Ref		0.25	(− 0.07;0.57)	0.97	0.51	(0.18;0.85)	0.09	0.10
*6*	PC ae C38:2	0.19	(0.05;0.33)	**0.04**	Ref		0.32	(− 0.01;0.65)	0.97	0.47	(0.13;0.81)	0.09	0.10
*7*	PC ae C38:3	0.19	(0.05;0.32)	**0.04**	Ref		0.23	(− 0.08;0.54)	0.97	0.49	(0.16;0.81)	0.09	0.10
*8*	PC ae C40:2	0.23	(0.10;0.37)	**0.01**	Ref		0.06	(− 0.27;0.38)	0.97	0.50	(0.16;0.83)	0.09	0.10
*9*	SM (OH) C14:1	0.22	(0.09;0.34)	**0.01**	Ref		0.11	(− 0.18;0.41)	0.97	0.44	(0.13;0.75)	0.09	0.10
*10*	SM (OH) C24:1	0.28	(0.14;0.42)	**0.01**	Ref		0.06	(− 0.27;0.39)	0.97	0.52	(0.18;0.87)	0.09	0.10
*11*	PC ae C36:3	0.23	(0.10;0.37)	**0.01**	Ref		0.27	(− 0.05;0.60)	0.97	0.45	(0.11;0.79)	0.09	0.10
*12*	PC aa C30:0	0.23	(0.09;0.38)	**0.02**	Ref		0.22	(− 0.13;0.57)	0.97	0.47	(0.11;0.84)	0.10	0.11
*13*	PC ae C34:1	0.23	(0.10;0.37)	**0.01**	Ref		0.25	(− 0.07;0.57)	0.97	0.44	(0.11;0.78)	0.09	0.11
*14*	PC ae C34:2	0.26	(0.12;0.40)	**0.01**	Ref		0.25	(− 0.08;0.58)	0.97	0.44	(0.09;0.78)	0.10	0.11
*15*	PC ae C36:0	0.25	(0.10;0.40)	**0.01**	Ref		0.26	(− 0.09;0.61)	0.97	0.48	(0.12;0.84)	0.09	0.11
Dietary pattern: carnivore^a^													
No. of participants		195			65		65			65			
Mean score (range)		0.0 (− 5.2–1.5)			− 0.9 (− 5.2 to − 0.1)		0.2 (− 0.1–0.4)			0.7 (0.4–1.5)			
*1*	PC aa C38:0	0.32	(0.18;0.45)	**0.001**	Ref		0.31	(− 0.01;0.64)	0.91	0.67	(0.33;1.01)	**0.01**	**0.02**
*2*	PC ae C38:6	0.26	(0.12;0.39)	**0.01**	Ref		0.24	(− 0.08;0.56)	0.91	0.68	(0.35;1.01)	**0.01**	**0.02**
*3*	PC ae C36:5	0.18	(0.04;0.32)	0.12	Ref		0.19	(− 0.14;0.51)	0.91	0.66	(0.32;0.99)	**0.01**	0.05
*4*	PC aa C36:0	0.22	(0.09;0.36)	0.03	Ref		0.18	(− 0.15;0.52)	0.91	0.59	(0.25;0.94)	**0.02**	0.08
*5*	PC ae C38:5	0.20	(0.06;0.34)	0.05	Ref		0.24	(− 0.09;0.57)	0.91	0.58	(0.24;0.92)	**0.02**	0.08
*6*	PC aa C38:6	0.27	(0.13;0.40)	0.01	Ref		0.19	(− 0.15;0.53)	0.91	0.51	(0.16;0.86)	0.06	0.10
*7*	PC aa C40:6	0.29	(0.16;0.43)	0.00	Ref		0.10	(− 0.23;0.43)	0.91	0.50	(0.16;0.84)	0.06	0.10
*8*	PC aa C42:2	0.13	(− 0.01;0.27)	0.35	Ref		0.22	(− 0.11;0.56)	0.91	0.50	(0.15;0.84)	0.06	0.10
*9*	PC ae C36:4	0.24	(0.10;0.38)	0.02	Ref		0.25	(− 0.10;0.59)	0.91	0.52	(0.16;0.87)	0.06	0.10
*10*	PC ae C40:6	0.26	(0.12;0.39)	0.01	Ref		0.19	(− 0.13;0.51)	0.91	0.51	(0.18;0.85)	0.06	0.10
*11*	Isoleucine	0.12	(− 0.02;0.26)	0.35	Ref		0.23	(− 0.10;0.57)	0.91	0.45	(0.10;0.80)	0.10	0.10
*12*	Methionine	0.13	(− 0.01;0.28)	0.33	Ref		0.23	(− 0.11;0.57)	0.91	0.44	(0.09;0.79)	0.10	0.10
*13*	Threonine	0.18	(0.04;0.33)	0.12	Ref		0.34	(− 0.01;0.68)	0.91	0.45	(0.09;0.80)	0.10	0.10
*14*	Tryptophan	0.18	(0.04;0.32)	0.12	Ref		0.35	(0.01;0.70)	0.91	0.49	(0.13;0.85)	0.08	0.10
*15*	Tyrosine	0.10	(− 0.05;0.24)	0.56	Ref		0.30	(− 0.05;0.65)	0.91	0.45	(0.09;0.81)	0.10	0.10
Dietary pattern: prudent^a^													
No. of participants		195			65		65			65			
Mean score (range)		0.0 (− 3.1–1.8)			− 1.1 (− 3.1 to − 0.3)		0.2 (− 0.2–0.5)			1.0 (0.5–1.8)			
*1*	PC aa C40:4	− 0.22	(− 0.39;− 0.06)	0.11	Ref		− 0.39	(− 0.74;− 0.04)	0.68	− 0.54	(− 0.9;− 0.18)	0.09	0.10
*2*	PC ae C30:2	0.20	(0.06;0.34)	0.10	Ref		0.42	(0.13;0.72)	0.68	0.50	(0.20;0.81)	0.09	0.10
*3*	SM (OH) C14:1	0.20	(0.06;0.35)	0.10	Ref		0.27	(− 0.03;0.58)	0.73	0.50	(0.18;0.81)	0.09	0.10
*4*	SM (OH) C16:1	0.22	(0.08;0.37)	0.10	Ref		0.26	(− 0.06;0.57)	0.73	0.51	(0.18;0.83)	0.09	0.10
*5*	C16	− 0.27	(− 0.44;− 0.11)	0.06	Ref		− 0.39	(− 0.74;− 0.05)	0.68	− 0.56	(− 0.92; − 0.20)	0.09	0.10
*6*	SM (OH) C22:2	0.20	(0.06;0.34)	0.10	Ref		0.30	(0.00;0.6)	0.68	0.46	(0.15;0.77)	0.09	0.12
*7*	C18:2	− 0.28	(− 0.45;− 0.11)	0.06	Ref		− 0.05	(− 0.41;0.31)	0.91	− 0.54	(− 0.91;-0.17)	0.09	0.12
*8*	C18:1	− 0.28	(− 0.45;− 0.11)	0.06	Ref		− 0.21	(− 0.57;0.15)	0.91	− 0.52	(− 0.90;-0.15)	0.11	0.13
*9*	SM (OH) C22:1	0.20	(0.05;0.35)	0.11	Ref		0.29	(− 0.03;0.61)	0.73	0.43	(0.10;0.76)	0.17	0.19
*10*	C18	− 0.23	(− 0.40;− 0.07)	0.10	Ref		− 0.32	(− 0.67;0.03)	0.73	− 0.47	(− 0.84;− 0.11)	0.17	0.19
*11*	lysoPC a C16:1	− 0.28	(− 0.45;− 0.11)	0.06	Ref		− 0.35	(− 0.71;0.01)	0.68	− 0.44	(− 0.82;− 0.06)	0.21	0.20
*12*	PC ae C40:6	0.13	(− 0.03;0.29)	0.47	Ref		0.26	(− 0.07;0.59)	0.73	0.42	(0.07;0.76)	0.19	0.20
*13*	Aspartate	− 0.12	(− 0.30;0.05)	0.52	Ref		− 0.06	(− 0.42;0.30)	0.91	− 0.46	(− 0.83;− 0.08)	0.19	0.20
*14*	Tryptophan	0.18	(0.01;0.35)	0.25	Ref		0.13	(− 0.23;0.48)	0.91	0.44	(0.08;0.81)	0.19	0.20
*15*	Sarcosine	− 0.18	(− 0.35;− 0.01)	0.25	Ref		− 0.34	(− 0.70;0.01)	0.68	− 0.41	(− 0.78;− 0.04)	0.26	0.20

^a^Tested using multiple linear regression models analyzing associations of dietary patterns (continuous, using log transformed Z-standardized scores, and in tertiles) as independent variable and log transformed Z-standardized metabolite concentrations as dependent variable. The continuous analysis is presented per each increase in SD (equaling 1) for the Western, Carnivore, and Prudent pattern, respectively. Tertile cut-off scores were − 0.3 and 0.5, − 0.1 and 0.4, and − 0.3 and 0.5 for the Western, Carnivore, and Prudent pattern, respectively. Regression models were adjusted for sex, age, analytical batch, body mass index (continuous), smoking status, and stage

^b^*p *value adjusted for multiple testing, using false discovery rate (FDR)

^c^In total, the Western pattern was statistically significantly associated (pFDR < 0.05) with 35 plasma metabolites (see Supplementary Table S2), of which the top-15 metabolites were presented here

A linear trend was observed between increasing tertiles of the Carnivore pattern and higher concentrations of two phosphatidylcholines. Similarly, every SD increase in the intake of the Carnivore pattern was also statistically significantly associated with higher concentrations of PC aa C38:0 (*p*_FDR_: 0.001) and PC ae 38:6 (*p*_FDR_: 0.01).

The Prudent pattern was not statistically significantly associated with any metabolite when evaluating the linear trend, as well as when testing each SD increase in consumption of the Prudent pattern. Results of the analyses between the dietary patterns and all 134 metabolites are provided in Supplementary Table S2.

### Overlap in the top-15 metabolites

**Fig. 2:**
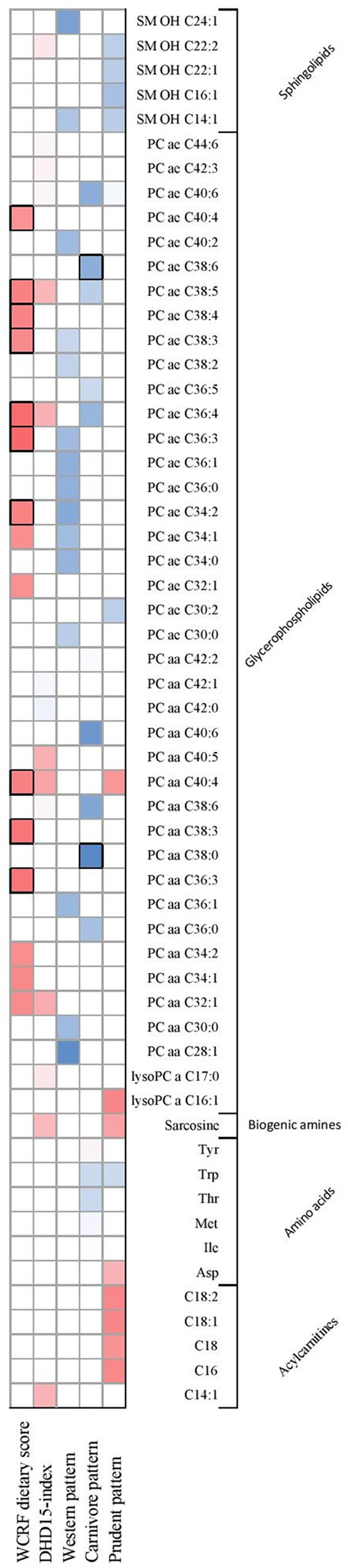
Heatmap illustrating the observed top-15 metabolites (based on the smallest *p*-value for trend over tertiles) associated with the diet quality indices, i.e. the WCRF dietary score and DHD15-index, and the dietary patterns, i.e. the Western, Carnivore, and Prudent pattern. The color is correlated to the observed β values; a darker blue color corresponds with a more positive association, while a darker red color represents a more inverse association between the dietary exposure and the plasma metabolite. Statistically significant associations are presented by a black box around the cell

Figure 2 illustrates the overlap in the observed top-15 metabolites (based on the smallest *p*-value for trend over tertiles) for each of the respective diet quality indices and dietary patterns. No overlap among acylcarnitines in the top-15 metabolites for each of the dietary exposures was observed. One amino acid was overlapping; the Carnivore and Prudent pattern both showed a positive association with plasma tryptophan. Sarcosine, a biogenic amine, was positively associated with both the DHD15-index and the Prudent pattern.

Several glycerophospholipids showed overlap between the investigated dietary exposures. An increasing adherence to the WCRF dietary score recommendations, as well as an increasing adherence to the DHD15-index recommendations were associated with decreasing concentrations of plasma phosphatidylcholine PC aa C32:1. Positive associations were observed between the DHD15-index and the Carnivore pattern and PC aa C38:6 concentrations. Inverse associations were observed for the WCRF dietary score, the DHD15-index and the Prudent pattern in relation to phosphatidylcholine PC aa C40:4.

Four phosphatidylcholines (PC ae 34:1, PC ae C34:2, PC ae C36:3, and PC ae C38:3) were among the top-15 metabolites associated with the WCRF dietary score and the Western pattern. Concentrations of these phosphatidylcholines were lower with increasing adherence to the WCRF dietary recommendations, while higher concentrations were observed with a higher consumption of the Western pattern. In addition, the WCRF dietary score and DHD15-index were inversely associated with two phosphatidylcholines (PC ae C36:4 and PC ae C38:5), while the opposite was observed for the Carnivore pattern. A higher DHD15-index and a higher intake of the Carnivore and Prudent pattern were associated with higher concentrations of PC ae C40:6.

Two sphingolipids overlapped between the dietary exposures. A higher consumption of the Western and Prudent pattern were both associated with a higher consumption of plasma SM (OH) C14:1. A higher DHD-15 index score reported higher concentrations of SM (OH) C22:2, and, in line, higher intakes of the Prudent pattern showed higher concentrations of SM (OH) C:22.

### Sensitivity analyses

Sensitivity analyses excluding patients from whom blood was collected during or after any type of treatment, i.e. (neo-) adjuvant chemotherapy and/or surgery (*n* = 19) and excluding stage IV patients (*n* = 7) showed similar beta coefficients between the dietary exposures and plasma metabolite concentrations compared to the main analysis (data not shown).

## Discussion

The aim of the current study was to explore the associations between the diet, evaluated using diet quality indices and dietary patterns, and plasma metabolite concentrations in colorectal cancer patients. The WCRF dietary score and the Carnivore pattern were observed to be statistically significantly associated with several long-chain phosphatidylcholines. In addition, when exploring the overlap in the top-15 metabolites for the respective dietary exposures, several dietary exposures were associated with identical long-chain phosphatidylcholines, which strengthens the hypothesis that diet and plasma metabolite concentrations might be associated in colorectal cancer patients.

Better adherence to the WCRF dietary and DHD guidelines, reflecting a healthier diet, was, in general, associated with lower concentrations of phosphatidylcholines in colorectal cancer patients in this study. In contrast, higher intakes of the Western pattern, which is generally regarded as an unhealthier diet [22, 43–46], showed higher concentrations of phosphatidylcholines. Similarly, a higher intake of the Carnivore pattern was positively associated with phosphatidylcholines in the current study, suggesting that a higher intake of a diet with red and processed meat, poultry, fish, and eggs is associated with higher levels of phosphatidylcholines. Interestingly, a study by Schmidt et al*.* reported that a vegan diet was characterized by lower concentrations of phosphatidylcholines and sphingolipids compared to a diet high in animal products [12]. A previous study among healthy participants also reported decreased lipid concentrations, including lysophosphatidylcholines and other glycerophospholipids, after a two-month intervention assigning healthy individuals to a Mediterranean diet, which is generally low in animal products, except for fish, compared to a control diet. The control diet was based on the American Heart Association guidelines [47], which recommend to consume low-fat dairy products, fish, poultry, and lean meats regularly. In line with our results and the study of Schmidt et al*.* [12], this may suggest that a higher intake of animal products is associated with higher phosphatidylcholine concentrations, also among those with colorectal cancer. Further studies are needed to elucidate whether phosphatidylcholine metabolism may play a role in the colorectal cancer continuum.

Diet quality indices and dietary patterns have been linked to colorectal cancer survival previously [22, 48, 49]. Associations between dietary exposures and circulating metabolites in colorectal cancer patients may provide important leads for future research regarding the underlying mechanisms between diet and colorectal cancer progression and survival. When these underlying mechanisms are identified, there is more solid scientific evidence to make nutritional recommendations for colorectal cancer survivors. However, since the current study is based on observational data only, it is not possible to clearly determine the causal relationships between dietary exposures and phosphatidylcholines in colorectal cancer patients. A previous study suggested that cancer cells display an elevated production of phosphatidylcholines, as part of enhanced lipogenesis in cancer cells [50], to further promote proliferation and evade apoptosis [51]. Given our findings that diet seems to be associated with phosphatidylcholines in colorectal cancer patients, and thus may theoretically support the hypothesized neoplastic growth, further studies studying phosphatidylcholines in relation to colorectal cancer recurrence and survival might be of interest.

The main strength of the current study is that this is, to the best of our knowledge, the first study investigating the associations between dietary exposures and plasma metabolites in a diseased population, i.e. colorectal cancer patients, using different approaches. When exploring the top-15 metabolites associated with the investigated dietary exposures, several phosphatidylcholines were observed to overlap between our exposures. This may indicate that the reported associations in our study population between dietary exposures and plasma metabolites are robust findings.

One of the limitations is that non-fasted blood samples were used for the current study and, as a result, we cannot rule out the possibility that some observed associations might be related to recent occasional dietary intake [38]. Our study was also limited to the metabolites included in the kit, while other metabolites might also be associated with the various dietary exposures. Following the presented results, a lipid-focused approach is of interest when investigating the association between diet and metabolites in colorectal cancer patients in the future. Lipid species, such as phosphatidylcholines, possess different physicochemical properties [52], and the methods used in the current and previous studies [11, 12] do not allow in-depth interpretation of the individual fatty acid compositions. Our relatively small sample size did not allow comparison of different colorectal cancer stages [40] and subtypes, although associations between diet and metabolites could potentially be related to specific tumor characteristics [53]. Lastly, we were not able to analyze the potential associations between diet, metabolites and colorectal cancer recurrence or survival.

In summary, we reported that the WCRF dietary score and the Carnivore pattern are associated with plasma concentrations of phosphatidylcholines in colorectal cancer patients. Several phosphatidylcholines were also observed to overlap between the dietary exposures when comparing the top-15 metabolites. Our findings should be replicated in larger study populations to allow more in-depth analysis regarding colorectal cancer stages and subtypes to also explore the role of nutritional metabolites in the colorectal cancer continuum. Furthermore, future studies should investigate the association between nutritional metabolites and colorectal cancer recurrence and survival. These explorative analyses might provide additional information about the potential underlying mechanisms of dietary intake in colorectal cancer patients, and the potential relationship with recurrence and survival.

## Supplementary Information

Below is the link to the electronic supplementary material.

Supplementary file1 (DOCX 17 KB)

Supplementary file2 (PDF 935 KB)

Supplementary file3 (DOCX 20 KB)

## Data Availability

Because the data consist of identifying cohort information, some access restrictions apply, and therefore, they cannot be made publicly available. Data will be shared with permission, from the acting committee of the COLON Study. Requests for data can be sent to Dr. Fränzel van Duijnhoven, Division of Human Nutrition and Health, Wageningen University and Research, Netherlands (e-mail: franzel.vanduijnhoven@wur.nl).

## Abbreviations

AICR: American Institute for Cancer Research
BMI: Body mass index
DHD15-index: Dutch Healthy Diet index 2015
FDR: False Discovery Rate
FFQ: Food Frequency Questionnaire
FIA-MS/MS: Flow injection analysis-tandem mass spectrometry
IARC: International Agency for Research on Cancer
LOD: Limit of detection
LysoPC a: Lysophosphatidylcholine (acyl)
SD: Standard deviation
SM (OH): (Hydroxy) sphingomyelin
SQUASH: Short QUestionnaire to ASsess Health enhancing physical activity
PC aa: Phosphatidylcholine (diacyl)
PC ae: Phosphatidylcholine (acyl-alkyl)
UHPLC-MS/MS: Ultra-high performance liquid chromatography-tandem mass spectrometry
WCRF: World Cancer Research Fund
