# Supplementary material for: Diet quality indices and dietary patterns are associated with plasma metabolites in colorectal cancer patients
Source: Eur J Nutr. 2021 Feb 5;60(6):3171–84. doi: 10.1007/s00394-021-02488-1 (PMC8354955; doi:10.1007/s00394-021-02488-1)
Supplement: Supplementary file 1 — Supplementary file1 (DOCX 17 KB) [file 394_2021_2488_MOESM1_ESM.docx]

**Supplementary Table S1** Food group composition

| **Food group** | **Food items** |
| --- | --- |
| Alcohol-free beer | All types of alcohol-free beer |
| Beer | All types of beer |
| Butter, hard margarines and cooking fats | Butter, low-fat butter, margarine in pack, solid backing/frying fat, lard |
| Coffee | All types of coffee |
| Eggs | Boiled and fried eggs |
| Fish | All types of fish, except for fried fish |
| Fruits | All types of fruits |
| High-fat dairy | High-fat cheese, milk, yoghurt, and quark |
| Legumes | Legumes and vegetables such as green beans, string beans, peas |
| Light beverages | All types of light beverages such as light soda |
| Liquor | All types of liquor |
| Low-fat dairy | Low-fat cheese, milk, yoghurt, and quark |
| Medium-fat dairy | Medium-fat cheese, milk, yoghurt, and quark |
| Nuts and seeds | All types of nuts and seeds |
| Pastry and biscuits | Croissants, pancakes, cake, biscuits, pastry |
| Pizza | All types of pizza |
| Potatoes | All types of potatoes |
| Poultry | All types of poultry |
| Processed meat | Sausages, bacon, ribs, ham, cold cuts, unknown types of meat |
| Red meat | Steak, organ meat, beef roll, pork tenderloin, minced meat |
| Refined grain products | Refined grain cereals, pasta, rice, and breads |
| Savory bread spreads | Fish salad, salads on biscuits, yeast extract such as Marmite, peanut butter |
| Savory sauces | All types of savory sauces such as mayonnaise, ketchup, sate sauce and dressings based on mayonnaise |
| Snacks | Chips, meatballs, snack products and fried products such as fried fish, French fries, Dutch snacks |
| Soft margarines, liquid cooking fats, vegetable oils and dressings | Olive oil, halvarine, liquid margarine and baking/frying fat, diet halvarine, oil/vinegar dressing |
| Soups | All types of soups |
| Soy and vegetarian products | Soy milk and all types of meat substitutes |
| Sugar and sugary products | Sweet spreads, candy, chocolate products, sugar in coffee and tea |
| Tea | All types of tea |
| Vegetables | All types of vegetables |
| Water | All types of mineral water |
| Whole grain products | Whole grain cereals, pasta, rice, and breads |
| Wine | All types of wine |
