# Supplementary material for: Diet quality indices and dietary patterns are associated with plasma metabolites in colorectal cancer patients
Source: Eur J Nutr. 2021 Feb 5;60(6):3171–84. doi: 10.1007/s00394-021-02488-1 (PMC8354955; doi:10.1007/s00394-021-02488-1)
Supplement: Supplementary file 2 — Supplementary file2 (PDF 935 KB) [file 394_2021_2488_MOESM2_ESM.pdf]

Supplementary Table S2. The associations between dietary exposures and plasma metabolites [1]

			WCRF dietary score [5]															
			Continuous				Tertile 1		Tertile 2					Tertile 3				
**Metabolite**	**Abbreviation**	Mean ± SD [2]	β	95% CI [3]	*p* -value	p_{FDR} [4]	Mean ± SD [2]	β	Mean ± SD [2]	β	95% CI [3]	*p* -value	p_{FDR} [4]	Mean ± SD [2]	β	95% CI [3]	*p* -value	p_{FDR} [4]
**ACYLCARNITINES**																		
Carnitine	C0	36.0 ± 7.3	0.06	(-0.16;0.27)	0.592	0.802	36.7 ± 7.5	ref.	35.2 ± 7.4	-0.14	(-0.50;0.21)	0.437	0.869	35.7 ± 7.1	-0.07	(-0.41;0.27)	0.694	0.848
Acetylcarnitine	C2	6.0 ± 1.9	-0.02	(-0.24;0.19)	0.826	0.923	6.2 ± 2.3	ref.	5.6 ± 1.6	-0.19	(-0.53;0.16)	0.298	0.756	5.9 ± 1.6	-0.07	(-0.40;0.27)	0.696	0.848
Propionylcarnitine	C3	0.5 ± 0.2	0.11	(-0.10;0.32)	0.313	0.645	0.5 ± 0.1	ref.	0.5 ± 0.2	0.11	(-0.24;0.46)	0.530	0.869	0.5 ± 0.1	0.10	(-0.23;0.43)	0.562	0.794
Butyrylcarnitine	C4	0.2 ± 0.1	-0.11	(-0.32;0.11)	0.333	0.665	0.2 ± 0.1	ref.	0.2 ± 0.1	0.11	(-0.24;0.47)	0.530	0.869	0.2 ± 0.1	-0.19	(-0.53;0.14)	0.264	0.520
Valerylcarnitine	C5	0.2 ± 0.1	0.02	(-0.18;0.22)	0.815	0.918	0.2 ± 0.1	ref.	0.2 ± 0.1	0.13	(-0.20;0.46)	0.433	0.869	0.2 ± 0.1	-0.01	(-0.32;0.31)	0.954	0.990
Decenoylcarnitine	C10:1	0.1 ± 0.1	-0.04	(-0.26;0.17)	0.701	0.862	0.1 ± 0.1	ref.	0.1 ± 0.1	-0.20	(-0.56;0.15)	0.258	0.741	0.1 ± 0.1	0.02	(-0.32;0.35)	0.928	0.979
Tetradecenoylcarnitine	C14:1	0.05 ± 0.02	-0.17	(-0.38;0.04)	0.107	0.411	0.06 ± 0.03	ref.	0.05 ± 0.03	-0.36	(-0.70;-0.02)	**0.042**	0.471	0.05 ± 0.02	-0.18	(-0.50;0.15)	0.297	0.560
Tetradecadienylcarnitine	C14:2	0.02 ± 0.01	-0.15	(-0.36;0.06)	0.161	0.473	0.03 ± 0.01	ref.	0.02 ± 0.01	-0.31	(-0.65;0.04)	0.081	0.488	0.02 ± 0.01	-0.13	(-0.46;0.20)	0.428	0.727
Hexadecanoylcarnitine	C16	0.10 ± 0.03	-0.23	(-0.44;-0.02)	**0.033**	0.222	0.11 ± 0.03	ref.	0.10 ± 0.03	-0.38	(-0.72;-0.03)	**0.033**	0.471	0.10 ± 0.03	-0.36	(-0.69;-0.03)	**0.032**	0.151
Octadecanoylcarnitine	C18	0.05 ± 0.01	-0.18	(-0.40;0.03)	0.088	0.358	0.05 ± 0.01	ref.	0.05 ± 0.01	-0.36	(-0.71;-0.02)	**0.041**	0.471	0.05 ± 0.01	-0.29	(-0.62;0.04)	0.086	0.253
Octadecenoylcarnitine	C18:1	0.09 ± 0.03	-0.12	(-0.34;0.10)	0.292	0.631	0.09 ± 0.03	ref.	0.08 ± 0.03	-0.30	(-0.66;0.06)	0.102	0.526	0.09 ± 0.03	-0.10	(-0.44;0.24)	0.573	0.794
Octadecadienylcarnitine	C18:2	0.03 ± 0.01	-0.03	(-0.25;0.19)	0.779	0.900	0.04 ± 0.01	ref.	0.03 ± 0.01	-0.21	(-0.56;0.15)	0.264	0.741	0.03 ± 0.01	-0.01	(-0.35;0.34)	0.975	0.990
**AMINO ACIDS**																		
Alanine	Ala	382.8 ± 100.7	0.16	(-0.05;0.38)	0.139	0.463	373.3 ± 103	ref.	400.1 ± 103.1	0.32	(-0.03;0.68)	0.077	0.488	379.0 ± 94.9	0.10	(-0.24;0.44)	0.550	0.794
Arginine	Arg	81.3 ± 23.5	0.07	(-0.15;0.28)	0.542	0.795	78.5 ± 22.6	ref.	82.6 ± 22.9	0.16	(-0.19;0.51)	0.377	0.815	83.8 ± 25.0	0.21	(-0.13;0.55)	0.221	0.479
Asparagine	Asn	43.5 ± 9.0	0.20	(-0.01;0.41)	0.061	0.273	42.2 ± 8.6	ref.	45.0 ± 9.6	0.31	(-0.03;0.66)	0.073	0.488	43.6 ± 9.0	0.22	(-0.10;0.55)	0.181	0.419
Aspartate	Asp	11.8 ± 4.0	-0.16	(-0.38;0.05)	0.142	0.463	11.9 ± 3.5	ref.	11.8 ± 4.0	-0.08	(-0.43;0.28)	0.676	0.917	11.7 ± 4.6	-0.21	(-0.55;0.13)	0.222	0.479
Citrulline	Cit	36.5 ± 10.2	-0.11	(-0.33;0.11)	0.333	0.665	36.8 ± 9.1	ref.	36.3 ± 10.6	-0.13	(-0.49;0.23)	0.487	0.869	36.2 ± 11.2	-0.08	(-0.42;0.26)	0.641	0.818
Glutamine	Gln	696.5 ± 98.5	0.07	(-0.13;0.28)	0.482	0.778	687.5 ± 102.9	ref.	710.0 ± 100.2	0.34	(0.00;0.67)	**0.049**	0.488	695.6 ± 91.3	0.14	(-0.18;0.46)	0.377	0.674
Glutamate	Glu	80.0 ± 29.7	-0.04	(-0.25;0.17)	0.713	0.862	80.5 ± 29.8	ref.	77.1 ± 26.9	-0.12	(-0.47;0.23)	0.505	0.869	82.0 ± 32.1	-0.04	(-0.37;0.30)	0.831	0.933
Glycine	Gly	215.6 ± 59.8	-0.02	(-0.21;0.18)	0.868	0.938	221.1 ± 70.8	ref.	211.3 ± 54.3	-0.09	(-0.41;0.24)	0.597	0.915	212.7 ± 49.0	-0.12	(-0.43;0.19)	0.463	0.756
Histidine	His	71.1 ± 12.4	0.08	(-0.13;0.29)	0.467	0.773	69.9 ± 11.1	ref.	74.5 ± 13	0.40	(0.06;0.74)	**0.023**	0.392	69.6 ± 13.0	0.00	(-0.33;0.32)	0.985	0.993
Isoleucine	Ile	75.4 ± 23.9	0.21	(0.01;0.42)	**0.044**	0.263	69.0 ± 19.5	ref.	81.0 ± 23.8	0.54	(0.21;0.87)	**0.002**	0.167	78.3 ± 27.4	0.42	(0.1;0.73)	**0.010**	0.087
Leucine	Leu	133.8 ± 42.3	0.15	(-0.06;0.36)	0.168	0.473	123.9 ± 34.9	ref.	143.7 ± 41.3	0.50	(0.17;0.84)	**0.004**	0.167	137.1 ± 49.1	0.30	(-0.03;0.62)	0.073	0.229
Lysine	Lys	193.5 ± 41.8	0.13	(-0.08;0.34)	0.234	0.550	186.0 ± 39.1	ref.	201.7 ± 37.9	0.43	(0.09;0.77)	**0.015**	0.327	195.4 ± 47.0	0.22	(-0.10;0.54)	0.186	0.421
Methionine	Met	20.20 ± 5.8	0.19	(-0.01;0.40)	0.069	0.298	19.1 ± 5.6	ref.	21.4 ± 5.5	0.45	(0.11;0.79)	**0.010**	0.264	20.7 ± 6.1	0.35	(0.02;0.67)	**0.037**	0.159
Ornithine	Orn	71.3 ± 24.6	0.03	(-0.19;0.24)	0.796	0.912	71.3 ± 26.9	ref.	71.0 ± 22.3	0.06	(-0.30;0.41)	0.759	0.917	71.6 ± 23.9	0.07	(-0.27;0.40)	0.695	0.848
Phenylalanine	Phe	62.8 ± 13.6	0.06	(-0.14;0.27)	0.548	0.795	61.1 ± 13.1	ref.	64.4 ± 12.7	0.21	(-0.13;0.55)	0.223	0.741	63.4 ± 15.0	0.15	(-0.17;0.48)	0.362	0.655
Proline	Pro	242.6 ± 79.5	0.21	(0.00;0.42)	0.051	0.263	224.2 ± 71.8	ref.	266.6 ± 84.9	0.52	(0.19;0.86)	**0.003**	0.167	243.8 ± 79.0	0.30	(-0.02;0.63)	0.065	0.217
Serine	Ser	93 ± 20.3	0.00	(-0.22;0.21)	0.994	0.994	92.8 ± 19.9	ref.	93.7 ± 20.8	-0.01	(-0.36;0.35)	0.964	0.965	92.6 ± 20.8	-0.05	(-0.39;0.29)	0.781	0.902
Threonine	Thr	108.7 ± 27.7	0.15	(-0.06;0.36)	0.165	0.473	105.3 ± 28.5	ref.	112.9 ± 27.0	0.33	(-0.01;0.68)	0.060	0.488	109.1 ± 27.3	0.24	(-0.08;0.57)	0.147	0.362
Tryptophan	Trp	53.1 ± 11.0	0.08	(-0.13;0.30)	0.442	0.759	52.2 ± 10.0	ref.	54.6 ± 10.6	0.18	(-0.17;0.53)	0.316	0.756	52.9 ± 12.4	0.01	(-0.33;0.34)	0.968	0.990
Tyrosine	Tyr	74.8 ± 24.1	0.06	(-0.15;0.28)	0.569	0.802	71.5 ± 21.4	ref.	78.2 ± 24.1	0.27	(-0.08;0.62)	0.132	0.591	75.7 ± 27.0	0.11	(-0.23;0.44)	0.527	0.794
Valine	Val	242.1 ± 64.0	0.09	(-0.11;0.30)	0.380	0.707	229.4 ± 53.6	ref.	258.4 ± 70.6	0.46	(0.12;0.79)	0.009	0.264	242.9 ± 67.2	0.20	(-0.13;0.52)	0.234	0.489
**BIOGENIC AMINES**																		
Creatinine	Creatinine	74.5 ± 17.4	0.04	(-0.14;0.21)	0.672	0.842	74.6 ± 15.5	ref.	73.4 ± 14.6	0.01	(-0.27;0.30)	0.927	0.955	75.3 ± 21.5	0.08	(-0.19;0.36)	0.551	0.794
Kynurenine	Kynurenine	2.7 ± 0.7	-0.12	(-0.32;0.08)	0.244	0.563	2.7 ± 0.8	ref.	2.6 ± 0.7	-0.09	(-0.43;0.24)	0.582	0.910	2.7 ± 0.8	-0.09	(-0.41;0.23)	0.600	0.796
Sarcosine	Sarcosine	4.4 ± 1.1	-0.21	(-0.42;0.01)	0.061	0.273	4.6 ± 1.2	ref.	4.3 ± 0.9	-0.26	(-0.61;0.09)	0.149	0.606	4.3 ± 1.1	-0.28	(-0.61;0.06)	0.105	0.292
Serotonin	Serotonin	0.8 ± 0.6	-0.08	(-0.29;0.14)	0.476	0.778	0.9 ± 0.6	ref.	0.7 ± 0.4	-0.23	(-0.59;0.12)	0.193	0.708	0.7 ± 0.6	-0.12	(-0.46;0.22)	0.482	0.770
Taurine	Taurine	120.9 ± 49.4	-0.03	(-0.24;0.19)	0.815	0.918	125.4 ± 51.9	ref.	114.3 ± 47	-0.24	(-0.59;0.12)	0.196	0.708	121.1 ± 48.4	-0.09	(-0.43;0.25)	0.588	0.794
Trans 4-Hydroxyproline	t4-OH-Pro	10.2 ± 5.0	-0.08	(-0.29;0.13)	0.450	0.759	10.4 ± 4.4	ref.	10.1 ± 4.5	0.04	(-0.31;0.38)	0.834	0.939	10.0 ± 6.1	-0.14	(-0.47;0.19)	0.402	0.691
Asymmetric dimethylarginine	ADMA	0.5 ± 0.1	0.06	(-0.14;0.25)	0.578	0.802	0.5 ± 0.1	ref.	0.4 ± 0.1	-0.03	(-0.35;0.29)	0.851	0.942	0.5 ± 0.1	0.14	(-0.16;0.45)	0.362	0.655
Symmetric dimethylarginine	SDMA	0.5 ± 0.1	0.02	(-0.18;0.22)	0.863	0.938	0.5 ± 0.1	ref.	0.4 ± 0.1	-0.02	(-0.35;0.31)	0.908	0.955	0.5 ± 0.1	0.09	(-0.22;0.40)	0.576	0.794
**GLYCEROPHOSPHOLIPIDS**																		
Lysophosphatidylcholine (acyl) C16:0	lysoPC a C16:0	90.4 ± 20.1	-0.04	(-0.26;0.18)	0.714	0.862	91.9 ± 20.7	ref.	93.0 ± 21.6	0.07	(-0.29;0.43)	0.687	0.917	86.3 ± 17.5	-0.26	(-0.60;0.09)	0.143	0.362
Lysophosphatidylcholine (acyl) C16:1	lysoPC a C16:1	2.6 ± 0.9	-0.14	(-0.36;0.08)	0.207	0.514	2.7 ± 1.0	ref.	2.5 ± 0.8	-0.18	(-0.54;0.18)	0.324	0.761	2.4 ± 0.9	-0.35	(-0.69;-0.01)	**0.047**	0.185
Lysophosphatidylcholine (acyl) C17:0	lysoPC a C17:0	1.6 ± 0.5	0.05	(-0.16;0.26)	0.654	0.840	1.6 ± 0.5	ref.	1.6 ± 0.5	0.17	(-0.17;0.52)	0.330	0.762	1.5 ± 0.4	-0.07	(-0.40;0.26)	0.692	0.848
Lysophosphatidylcholine (acyl) C18:0	lysoPC a C18:0	26.4 ± 6.8	-0.05	(-0.27;0.16)	0.632	0.834	26.8 ± 7.5	ref.	27.1 ± 7.6	0.02	(-0.33;0.37)	0.916	0.955	25.2 ± 5	-0.19	(-0.52;0.15)	0.277	0.538
Lysophosphatidylcholine (acyl) C18:1	lysoPC a C18:1	17.9 ± 5.4	0.00	(-0.21;0.22)	0.991	0.994	18.4 ± 5.5	ref.	17.8 ± 5.4	-0.05	(-0.40;0.30)	0.780	0.917	17.6 ± 5.2	-0.09	(-0.43;0.25)	0.608	0.799
Lysophosphatidylcholine (acyl) C18:2	lysoPC a C18:2	29.2 ± 10.4	0.11	(-0.09;0.32)	0.289	0.631	29.6 ± 10.0	ref.	29.1 ± 11.1	-0.02	(-0.36;0.33)	0.927	0.955	28.9 ± 10.3	0.04	(-0.29;0.37)	0.813	0.931
Lysophosphatidylcholine (acyl) C20:3	lysoPC a C20:3	2.0 ± 0.7	-0.06	(-0.28;0.15)	0.557	0.795	2.1 ± 0.8	ref.	2.0 ± 0.6	-0.05	(-0.40;0.30)	0.765	0.917	2.0 ± 0.7	-0.21	(-0.54;0.13)	0.226	0.481
Lysophosphatidylcholine (acyl) C20:4	lysoPC a C20:4	5.9 ± 1.7	0.11	(-0.10;0.33)	0.305	0.638	5.8 ± 1.6	ref.	5.8 ± 1.5	0.05	(-0.31;0.40)	0.800	0.923	6.1 ± 2.0	0.06	(-0.28;0.39)	0.746	0.881
Lysophosphatidylcholine (acyl) C24:0	lysoPC a C24:0	0.14 ± 0.03	-0.01	(-0.21;0.18)	0.888	0.944	0.14 ± 0.04	ref.	0.14 ± 0.03	0.03	(-0.29;0.35)	0.846	0.942	0.13 ± 0.03	-0.05	(-0.36;0.26)	0.755	0.881
Lysophosphatidylcholine (acyl) C28:1	lysoPC a C28:1	0.3 ± 0.1	-0.05	(-0.23;0.12)	0.545	0.795	0.3 ± 0.1	ref.	0.3 ± 0.1	0.07	(-0.22;0.36)	0.637	0.915	0.3 ± 0.1	-0.12	(-0.39;0.16)	0.398	0.691
Phosphatidylcholine (diacyl) C28:1	PC aa C28:1	3.0 ± 0.8	-0.16	(-0.36;0.04)	0.117	0.436	3.0 ± 0.7	ref.	3.1 ± 0.8	0.04	(-0.28;0.37)	0.795	0.923	2.9 ± 0.8	-0.29	(-0.60;0.01)	0.063	0.217
Phosphatidylcholine (diacyl) C30:0	PC aa C30:0	4.0 ± 1.3	-0.15	(-0.37;0.06)	0.162	0.473	4.0 ± 1.2	ref.	4.3 ± 1.5	0.14	(-0.21;0.49)	0.431	0.869	3.8 ± 1.2	-0.26	(-0.60;0.07)	0.126	0.343
Phosphatidylcholine (diacyl) C32:0	PC aa C32:0	26.6 ± 5.5	-0.14	(-0.35;0.07)	0.183	0.480	26.9 ± 4.5	ref.	26.9 ± 5.7	-0.02	(-0.36;0.32)	0.918	0.955	26.1 ± 6.4	-0.25	(-0.57;0.08)	0.135	0.354
Phosphatidylcholine (diacyl) C32:1	PC aa C32:1	32.0 ± 16.2	-0.26	(-0.47;-0.05)	**0.019**	0.153	34.3 ± 17.3	ref.	32.2 ± 16.2	-0.16	(-0.51;0.19)	0.377	0.815	29 ± 14.2	-0.44	(-0.78;-0.11)	**0.010**	0.087
Phosphatidylcholine (diacyl) C32:2	PC aa C32:2	8.3 ± 3.1	-0.15	(-0.36;0.07)	0.181	0.480	8.4 ± 2.7	ref.	8.8 ± 3.5	0.02	(-0.32;0.37)	0.890	0.955	7.8 ± 3.1	-0.30	(-0.64;0.03)	0.073	0.229
Phosphatidylcholine (diacyl) C32:3	PC aa C32:3	0.9 ± 0.2	-0.01	(-0.20;0.19)	0.950	0.974	0.9 ± 0.2	ref.	0.9 ± 0.3	0.07	(-0.25;0.39)	0.682	0.917	0.9 ± 0.2	-0.12	(-0.43;0.19)	0.455	0.753
Phosphatidylcholine (diacyl) C34:1	PC aa C34:1	365.4 ± 82.8	-0.27	(-0.48;-0.06)	**0.013**	0.145	376.5 ± 78.8	ref.	367.1 ± 84.2	-0.12	(-0.46;0.23)	0.515	0.869	350.1 ± 85.4	-0.44	(-0.77;-0.10)	**0.011**	0.087
Phosphatidylcholine (diacyl) C34:2	PC aa C34:2	739.8 ± 128.1	-0.25	(-0.47;-0.04)	**0.022**	0.155	759.8 ± 116.0	ref.	749.6 ± 129.0	-0.12	(-0.47;0.23)	0.511	0.869	706.0 ± 136.6	-0.46	(-0.80;-0.13)	**0.007**	0.081
Phosphatidylcholine (diacyl) C34:3	PC aa C34:3	30.1 ± 10.2	-0.21	(-0.42;0.00)	**0.048**	0.263	31.2 ± 10.0	ref.	30.9 ± 10.8	-0.10	(-0.44;0.25)	0.584	0.910	28.1 ± 9.6	-0.40	(-0.72;-0.07)	**0.018**	0.128
Phosphatidylcholine (diacyl) C34:4	PC aa C34:4	3.1 ± 1.2	-0.11	(-0.32;0.10)	0.302	0.638	3.1 ± 1.1	ref.	3.3 ± 1.3	0.13	(-0.21;0.48)	0.446	0.869	2.9 ± 1.1	-0.28	(-0.61;0.05)	0.095	0.271
Phosphatidylcholine (diacyl) C36:0	PC aa C36:0	3.2 ± 1.0	-0.01	(-0.22;0.20)	0.916	0.967	3.2 ± 0.9	ref.	3.3 ± 1.0	0.16	(-0.18;0.50)	0.354	0.805	3.2 ± 1.2	-0.05	(-0.38;0.27)	0.755	0.881
Phosphatidylcholine (diacyl) C36:1	PC aa C36:1	62.9 ± 17.8	-0.24	(-0.44;-0.04)	**0.019**	0.153	64.6 ± 18.1	ref.	63.8 ± 18.4	-0.06	(-0.39;0.28)	0.736	0.917	60.1 ± 16.7	-0.34	(-0.66;-0.02)	**0.036**	0.159
Phosphatidylcholine (diacyl) C36:2	PC aa C36:2	384.7 ± 79.7	-0.24	(-0.44;-0.04)	**0.020**	0.153	393.7 ± 77.0	ref.	388.4 ± 80.8	-0.11	(-0.44;0.23)	0.532	0.869	370.3 ± 81.3	-0.36	(-0.67;-0.04)	**0.029**	0.151

			Continuous				Tertile 1		Tertile 2					Tertile 3				
**Metabolite**	**Abbreviation**	Mean ± SD [2]	β	95% CI [3]	*p* -value	p_{FDR} [4]	Mean ± SD [2]	β	Mean ± SD [2]	β	95% CI [3]	*p* -value	p_{FDR} [4]	Mean ± SD [2]	β	95% CI [3]	*p* -value	p_{FDR} [4]
Phosphatidylcholine (diacyl) C36:3	PC aa C36:3	192.6 ± 43.8	-0.34	(-0.54;-0.14)	**0.001**	**0.036**	201 ± 41.4	ref.	197.4 ± 45.4	-0.08	(-0.40;0.24)	0.629	0.915	177.8 ± 42.2	-0.59	(-0.90;-0.28)	**0.0003**	**0.011**
Phosphatidylcholine (diacyl) C36:4	PC aa C36:4	291.5 ± 72.0	-0.11	(-0.32;0.09)	0.277	0.619	291.8 ± 65.6	ref.	301.1 ± 79.5	0.08	(-0.25;0.42)	0.635	0.915	282.3 ± 72.6	-0.30	(-0.62;0.02)	0.072	0.229
Phosphatidylcholine (diacyl) C36:5	PC aa C36:5	45.8 ± 27.8	0.06	(-0.15;0.28)	0.558	0.795	43.8 ± 25.3	ref.	47.1 ± 21.9	0.32	(-0.03;0.67)	0.075	0.488	47.1 ± 34.9	0.07	(-0.27;0.40)	0.702	0.848
Phosphatidylcholine (diacyl) C36:6	PC aa C36:6	1.5 ± 0.6	0.05	(-0.16;0.26)	0.658	0.840	1.5 ± 0.6	ref.	1.6 ± 0.6	0.30	(-0.04;0.64)	0.089	0.488	1.5 ± 0.7	-0.02	(-0.35;0.31)	0.908	0.979
Phosphatidylcholine (diacyl) C38:0	PC aa C38:0	2.9 ± 0.9	-0.06	(-0.27;0.14)	0.555	0.795	2.8 ± 0.8	ref.	2.9 ± 0.8	0.06	(-0.27;0.40)	0.714	0.917	2.9 ± 1.0	-0.09	(-0.41;0.23)	0.576	0.794
Phosphatidylcholine (diacyl) C38:3	PC aa C38:3	53.2 ± 13.6	-0.34	(-0.53;-0.15)	**0.001**	**0.026**	55.1 ± 13.9	ref.	54.4 ± 14.2	-0.11	(-0.42;0.20)	0.487	0.869	49.7 ± 12.2	-0.53	(-0.82;-0.23)	**0.001**	**0.013**
Phosphatidylcholine (diacyl) C38:4	PC aa C38:4	131.5 ± 35.0	-0.12	(-0.32;0.07)	0.218	0.530	131 ± 32.5	ref.	133.4 ± 36.5	-0.02	(-0.34;0.30)	0.909	0.955	130.4 ± 37.0	-0.23	(-0.54;0.07)	0.132	0.353
Phosphatidylcholine (diacyl) C38:5	PC aa C38:5	66.2 ± 19.0	-0.05	(-0.25;0.16)	0.656	0.840	65.9 ± 17.9	ref.	68.2 ± 18.6	0.19	(-0.14;0.52)	0.264	0.741	64.9 ± 20.9	-0.16	(-0.48;0.15)	0.310	0.577
Phosphatidylcholine (diacyl) C38:6	PC aa C38:6	101.1 ± 33.6	0.08	(-0.12;0.29)	0.430	0.759	95 ± 27.6	ref.	107.1 ± 36.7	0.33	(-0.01;0.67)	0.059	0.488	103.3 ± 36.7	0.08	(-0.24;0.41)	0.625	0.813
Phosphatidylcholine (diacyl) C40:2	PC aa C40:2	0.3 ± 0.1	-0.04	(-0.24;0.17)	0.729	0.867	0.3 ± 0.1	ref.	0.3 ± 0.1	0.10	(-0.24;0.43)	0.571	0.910	0.3 ± 0.1	-0.10	(-0.42;0.22)	0.541	0.794
Phosphatidylcholine (diacyl) C40:3	PC aa C40:3	0.4 ± 0.1	-0.07	(-0.27;0.13)	0.493	0.786	0.4 ± 0.1	ref.	0.4 ± 0.1	0.04	(-0.29;0.37)	0.806	0.923	0.4 ± 0.2	-0.07	(-0.38;0.25)	0.671	0.848
Phosphatidylcholine (diacyl) C40:4	PC aa C40:4	3.0 ± 0.8	-0.30	(-0.51;-0.09)	**0.005**	0.085	3.2 ± 0.9	ref.	3.0 ± 0.8	-0.26	(-0.60;0.08)	0.139	0.602	2.8 ± 0.7	-0.52	(-0.85;-0.20)	**0.002**	**0.026**
Phosphatidylcholine (diacyl) C40:5	PC aa C40:5	8.3 ± 2.4	-0.20	(-0.41;0.01)	0.058	0.273	8.5 ± 2.6	ref.	8.5 ± 2.6	-0.02	(-0.36;0.32)	0.909	0.955	7.8 ± 2.0	-0.37	(-0.69;-0.04)	**0.028**	0.151
Phosphatidylcholine (diacyl) C40:6	PC aa C40:6	28.3 ± 10.2	0.06	(-0.14;0.27)	0.552	0.795	26.4 ± 8.6	ref.	29.6 ± 10.8	0.27	(-0.07;0.61)	0.117	0.560	29.6 ± 11.2	0.12	(-0.21;0.44)	0.482	0.770
Phosphatidylcholine (diacyl) C42:0	PC aa C42:0	0.3 ± 0.1	0.10	(-0.10;0.30)	0.349	0.667	0.3 ± 0.1	ref.	0.4 ± 0.1	0.17	(-0.16;0.50)	0.305	0.756	0.4 ± 0.1	0.12	(-0.19;0.43)	0.452	0.753
Phosphatidylcholine (diacyl) C42:1	PC aa C42:1	0.18 ± 0.05	0.02	(-0.18;0.22)	0.863	0.938	0.17 ± 0.05	ref.	0.18 ± 0.05	0.06	(-0.27;0.39)	0.716	0.917	0.2 ± 0.1	0.00	(-0.31;0.31)	0.993	0.993
Phosphatidylcholine (diacyl) C42:2	PC aa C42:2	0.16 ± 0.04	0.08	(-0.13;0.29)	0.450	0.759	0.15 ± 0.04	ref.	0.16 ± 0.04	0.10	(-0.24;0.44)	0.571	0.910	0.16 ± 0.05	0.10	(-0.22;0.43)	0.533	0.794
Phosphatidylcholine (diacyl) C42:4	PC aa C42:4	0.10 ± 0.02	-0.12	(-0.34;0.09)	0.265	0.601	0.11 ± 0.02	ref.	0.10 ± 0.02	-0.11	(-0.47;0.24)	0.531	0.869	0.10 ± 0.02	-0.30	(-0.63;0.04)	0.087	0.253
Phosphatidylcholine (diacyl) C42:5	PC aa C42:5	0.3 ± 0.1	-0.05	(-0.26;0.17)	0.678	0.842	0.3 ± 0.1	ref.	0.3 ± 0.1	0.15	(-0.20;0.50)	0.406	0.864	0.3 ± 0.1	-0.08	(-0.42;0.26)	0.640	0.818
Phosphatidylcholine (diacyl) C42:6	PC aa C42:6	0.4 ± 0.1	-0.01	(-0.22;0.21)	0.952	0.974	0.4 ± 0.1	ref.	0.4 ± 0.1	0.09	(-0.26;0.44)	0.617	0.915	0.4 ± 0.1	0.03	(-0.30;0.36)	0.851	0.936
Phosphatidylcholine (acyl-alkyl) C30:0	PC ae C30:0	0.3 ± 0.1	-0.21	(-0.41;0.00)	**0.050**	0.263	0.3 ± 0.1	ref.	0.4 ± 0.1	-0.01	(-0.35;0.32)	0.943	0.958	0.3 ± 0.1	-0.36	(-0.68;-0.05)	**0.026**	0.145
Phosphatidylcholine (acyl-alkyl) C30:2	PC ae C30:2	0.08 ± 0.02	0.01	(-0.17;0.19)	0.925	0.968	0.08 ± 0.02	ref.	0.09 ± 0.02	0.17	(-0.13;0.46)	0.272	0.741	0.08 ± 0.02	-0.08	(-0.36;0.21)	0.592	0.794
Phosphatidylcholine (acyl-alkyl) C32:1	PC ae C32:1	5.3 ± 1.2	-0.24	(-0.45;-0.04)	**0.019**	0.153	5.4 ± 1.1	ref.	5.2 ± 1.2	-0.29	(-0.63;0.04)	0.085	0.488	5.1 ± 1.4	-0.44	(-0.76;-0.12)	**0.007**	0.081
Phosphatidylcholine (acyl-alkyl) C32:2	PC ae C32:2	1.4 ± 0.4	-0.15	(-0.35;0.05)	0.141	0.463	1.4 ± 0.3	ref.	1.4 ± 0.3	-0.15	(-0.47;0.17)	0.368	0.815	1.3 ± 0.4	-0.31	(-0.62;-0.01)	**0.048**	0.185
Phosphatidylcholine (acyl-alkyl) C34:0	PC ae C34:0	2.5 ± 0.6	-0.14	(-0.34;0.07)	0.200	0.514	2.5 ± 0.6	ref.	2.5 ± 0.7	0.03	(-0.31;0.36)	0.878	0.955	2.4 ± 0.7	-0.25	(-0.57;0.08)	0.138	0.356
Phosphatidylcholine (acyl-alkyl) C34:1	PC ae C34:1	18.0 ± 3.9	-0.25	(-0.45;-0.06)	**0.011**	0.137	18.5 ± 3.5	ref.	18.2 ± 4.3	-0.19	(-0.51;0.13)	0.235	0.741	17.3 ± 3.9	-0.41	(-0.72;-0.11)	**0.009**	0.083
Phosphatidylcholine (acyl-alkyl) C34:2	PC ae C34:2	20.2 ± 5.3	-0.30	(-0.50;-0.10)	**0.004**	0.084	21.5 ± 5.3	ref.	20.0 ± 5.1	-0.34	(-0.66;-0.01)	**0.042**	0.471	18.8 ± 5.3	-0.55	(-0.86;-0.24)	**0.001**	**0.013**
Phosphatidylcholine (acyl-alkyl) C34:3	PC ae C34:3	13.1 ± 4.0	-0.20	(-0.40;0.00)	0.056	0.273	13.8 ± 3.8	ref.	13.2 ± 3.9	-0.21	(-0.54;0.12)	0.212	0.741	12.3 ± 4	-0.43	(-0.74;-0.11)	**0.009**	0.083
Phosphatidylcholine (acyl-alkyl) C36:0	PC ae C36:0	1.2 ± 0.3	-0.26	(-0.47;-0.05)	**0.017**	0.153	1.2 ± 0.3	ref.	1.2 ± 0.3	-0.13	(-0.48;0.22)	0.463	0.869	1.1 ± 0.3	-0.34	(-0.67;0.00)	**0.048**	0.185
Phosphatidylcholine (acyl-alkyl) C36:1	PC ae C36:1	11.5 ± 2.7	-0.16	(-0.36;0.03)	0.105	0.411	11.6 ± 2.7	ref.	11.9 ± 2.8	0.05	(-0.27;0.38)	0.745	0.917	11.0 ± 2.6	-0.30	(-0.61;0.01)	0.057	0.207
Phosphatidylcholine (acyl-alkyl) C36:2	PC ae C36:2	21.7 ± 5.4	-0.15	(-0.34;0.04)	0.128	0.451	22.2 ± 5.5	ref.	22.0 ± 5.2	-0.06	(-0.38;0.26)	0.707	0.917	20.9 ± 5.4	-0.30	(-0.60;0.00)	0.055	0.205
Phosphatidylcholine (acyl-alkyl) C36:3	PC ae C36:3	11.4 ± 2.8	-0.38	(-0.58;-0.19)	**0.0001**	**0.019**	12.2 ± 2.8	ref.	11.3 ± 2.8	-0.37	(-0.69;-0.06)	**0.022**	0.392	10.4 ± 2.7	-0.67	(-0.97;-0.36)	**0.00002**	**0.003**
Phosphatidylcholine (acyl-alkyl) C36:4	PC ae C36:4	26.3 ± 6.4	-0.37	(-0.58;-0.16)	**0.001**	**0.026**	27.8 ± 5.9	ref.	26.4 ± 6.6	-0.29	(-0.63;0.04)	0.091	0.488	24.3 ± 6.3	-0.67	(-0.99;-0.35)	**0.0001**	**0.004**
Phosphatidylcholine (acyl-alkyl) C36:5	PC ae C36:5	18.3 ± 5.0	-0.15	(-0.35;0.06)	0.155	0.473	18.8 ± 4.5	ref.	18.4 ± 5	-0.09	(-0.42;0.25)	0.608	0.915	17.6 ± 5.4	-0.35	(-0.67;-0.03)	**0.033**	0.151
Phosphatidylcholine (acyl-alkyl) C38:0	PC ae C38:0	2.5 ± 0.8	0.04	(-0.16;0.25)	0.679	0.842	2.4 ± 0.7	ref.	2.6 ± 0.8	0.28	(-0.06;0.61)	0.108	0.535	2.5 ± 0.9	0.02	(-0.31;0.34)	0.926	0.979
Phosphatidylcholine (acyl-alkyl) C38:2	PC ae C38:2	2.2 ± 0.6	-0.20	(-0.40;0.00)	**0.047**	0.263	2.3 ± 0.5	ref.	2.2 ± 0.6	-0.13	(-0.45;0.20)	0.452	0.869	2.1 ± 0.6	-0.37	(-0.69;-0.06)	**0.020**	0.130
Phosphatidylcholine (acyl-alkyl) C38:3	PC ae C38:3	4.8 ± 1.1	-0.27	(-0.46;-0.08)	**0.006**	0.085	4.9 ± 1.1	ref.	4.9 ± 1.2	-0.05	(-0.36;0.26)	0.756	0.917	4.5 ± 1.0	-0.48	(-0.77;-0.18)	**0.002**	**0.026**
Phosphatidylcholine (acyl-alkyl) C38:4	PC ae C38:4	17.2 ± 3.6	-0.29	(-0.49;-0.09)	**0.005**	0.085	17.8 ± 3.3	ref.	17.4 ± 4.1	-0.24	(-0.57;0.08)	0.148	0.606	16.3 ± 3.5	-0.56	(-0.87;-0.25)	**0.001**	**0.013**
Phosphatidylcholine (acyl-alkyl) C38:5	PC ae C38:5	21.9 ± 4.7	-0.30	(-0.50;-0.10)	**0.004**	0.084	22.7 ± 4	ref.	22.0 ± 4.8	-0.22	(-0.55;0.11)	0.189	0.708	20.9 ± 5.1	-0.52	(-0.83;-0.21)	**0.001**	**0.025**
Phosphatidylcholine (acyl-alkyl) C38:6	PC ae C38:6	9.2 ± 2.6	-0.10	(-0.30;0.10)	0.343	0.665	9.2 ± 2.2	ref.	9.4 ± 2.6	0.05	(-0.28;0.38)	0.774	0.917	9.1 ± 3.1	-0.21	(-0.53;0.10)	0.192	0.429
Phosphatidylcholine (acyl-alkyl) C40:1	PC ae C40:1	1.1 ± 0.3	0.06	(-0.15;0.27)	0.593	0.802	1.1 ± 0.3	ref.	1.1 ± 0.3	0.19	(-0.15;0.54)	0.277	0.741	1.1 ± 0.3	-0.03	(-0.36;0.30)	0.858	0.936
Phosphatidylcholine (acyl-alkyl) C40:2	PC ae C40:2	1.6 ± 0.4	0.00	(-0.20;0.20)	0.989	0.994	1.6 ± 0.4	ref.	1.6 ± 0.4	0.08	(-0.26;0.41)	0.655	0.915	1.6 ± 0.4	-0.01	(-0.33;0.30)	0.941	0.985
Phosphatidylcholine (acyl-alkyl) C40:3	PC ae C40:3	0.8 ± 0.2	-0.20	(-0.39;-0.01)	**0.042**	0.263	0.9 ± 0.2	ref.	0.8 ± 0.2	-0.18	(-0.48;0.13)	0.268	0.741	0.8 ± 0.2	-0.35	(-0.64;-0.06)	**0.021**	0.130
Phosphatidylcholine (acyl-alkyl) C40:4	PC ae C40:4	2.2 ± 0.4	-0.24	(-0.44;-0.04)	**0.021**	0.153	2.3 ± 0.4	ref.	2.2 ± 0.5	-0.32	(-0.64;0.01)	0.057	0.488	2.1 ± 0.5	-0.50	(-0.80;-0.19)	**0.002**	**0.026**
Phosphatidylcholine (acyl-alkyl) C40:5	PC ae C40:5	3.4 ± 0.7	-0.16	(-0.35;0.04)	0.124	0.448	3.4 ± 0.6	ref.	3.4 ± 0.7	-0.12	(-0.44;0.21)	0.486	0.869	3.3 ± 0.7	-0.34	(-0.65;-0.03)	**0.031**	0.151
Phosphatidylcholine (acyl-alkyl) C40:6	PC ae C40:6	4.8 ± 1.2	0.03	(-0.17;0.23)	0.767	0.893	4.6 ± 1.2	ref.	4.9 ± 1.3	0.17	(-0.16;0.50)	0.315	0.756	4.8 ± 1.3	-0.02	(-0.33;0.30)	0.915	0.979
Phosphatidylcholine (acyl-alkyl) C42:1	PC ae C42:1	0.3 ± 0.1	-0.18	(-0.38;0.02)	0.079	0.333	0.3 ± 0.1	ref.	0.3 ± 0.1	-0.19	(-0.52;0.14)	0.252	0.741	0.3 ± 0.1	-0.37	(-0.68;-0.06)	**0.021**	0.130
Phosphatidylcholine (acyl-alkyl) C42:2	PC ae C42:2	0.4 ± 0.1	-0.07	(-0.26;0.13)	0.513	0.795	0.4 ± 0.1	ref.	0.4 ± 0.1	-0.07	(-0.39;0.25)	0.666	0.917	0.4 ± 0.1	-0.16	(-0.47;0.14)	0.294	0.560
Phosphatidylcholine (acyl-alkyl) C42:3	PC ae C42:3	0.6 ± 0.1	-0.10	(-0.30;0.10)	0.339	0.665	0.6 ± 0.1	ref.	0.6 ± 0.1	-0.08	(-0.41;0.25)	0.6370	0.915	0.5 ± 0.2	-0.22	(-0.54;0.09)	0.1639	0.392
Phosphatidylcholine (acyl-alkyl) C42:4	PC ae C42:4	0.7 ± 0.2	-0.15	(-0.35;0.06)	0.157	0.473	0.7 ± 0.1	ref.	0.7 ± 0.2	-0.30	(-0.63;0.03)	0.072	0.488	0.7 ± 0.2	-0.36	(-0.68;-0.05)	**0.026**	0.145
Phosphatidylcholine (acyl-alkyl) C42:5	PC ae C42:5	1.5 ± 0.3	-0.09	(-0.29;0.12)	0.416	0.754	1.6 ± 0.3	ref.	1.5 ± 0.3	-0.11	(-0.45;0.22)	0.507	0.869	1.5 ± 0.3	-0.19	(-0.51;0.13)	0.243	0.501
Phosphatidylcholine (acyl-alkyl) C44:3	PC ae C44:3	0.07 ± 0.02	-0.06	(-0.26;0.14)	0.541	0.795	0.07 ± 0.02	ref.	0.06 ± 0.01	-0.19	(-0.52;0.13)	0.251	0.741	0.07 ± 0.02	-0.11	(-0.42;0.21)	0.507	0.794
Phosphatidylcholine (acyl-alkyl) C44:4	PC ae C44:4	0.2 ± 0.1	-0.10	(-0.30;0.11)	0.359	0.678	0.2 ± 0.1	ref.	0.2 ± 0.1	-0.24	(-0.57;0.10)	0.169	0.666	0.2 ± 0.1	-0.28	(-0.60;0.04)	0.087	0.253
Phosphatidylcholine (acyl-alkyl) C44:5	PC ae C44:5	1.0 ± 0.3	-0.08	(-0.29;0.13)	0.447	0.759	1.0 ± 0.3	ref.	1.0 ± 0.3	-0.18	(-0.53;0.17)	0.310	0.756	1.0 ± 0.3	-0.19	(-0.52;0.14)	0.254	0.515
Phosphatidylcholine (acyl-alkyl) C44:6	PC ae C44:6	0.8 ± 0.2	0.06	(-0.14;0.26)	0.580	0.802	0.8 ± 0.2	ref.	0.8 ± 0.2	-0.05	(-0.39;0.28)	0.751	0.917	0.8 ± 0.2	-0.03	(-0.35;0.29)	0.859	0.936
**SPHINGOLIPIDS**																		
Hydroxysphingomyelin C14:1	SM OH C14:1	4.0 ± 1.0	-0.01	(-0.19;0.18)	0.933	0.969	4.0 ± 1.0	ref.	4.1 ± 1.1	0.01	(-0.29;0.31)	0.940	0.958	4.0 ± 1.0	-0.09	(-0.37;0.20)	0.560	0.794
Hydroxysphingomyelin C16:1	SM OH C16:1	1.9 ± 0.5	0.05	(-0.14;0.24)	0.635	0.834	1.8 ± 0.5	ref.	1.9 ± 0.5	0.07	(-0.24;0.39)	0.654	0.915	1.9 ± 0.5	0.03	(-0.27;0.33)	0.829	0.933
Hydroxysphingomyelin C22:1	SM OH C22:1	4.4 ± 1.1	-0.08	(-0.27;0.11)	0.412	0.754	4.4 ± 1.2	ref.	4.6 ± 1.0	0.12	(-0.20;0.44)	0.458	0.869	4.3 ± 1.0	-0.17	(-0.48;0.13)	0.259	0.518
Hydroxysphingomyelin C22:2	SM OH C22:2	3.3 ± 0.9	-0.01	(-0.20;0.17)	0.880	0.944	3.2 ± 0.9	ref.	3.5 ± 0.9	0.18	(-0.12;0.48)	0.232	0.741	3.2 ± 0.8	-0.09	(-0.38;0.19)	0.521	0.794
Hydroxysphingomyelin C24:1	SM OH C24:1	0.3 ± 0.1	-0.13	(-0.33;0.08)	0.224	0.537	0.3 ± 0.1	ref.	0.3 ± 0.1	0.01	(-0.33;0.35)	0.965	0.965	0.3 ± 0.1	-0.24	(-0.56;0.08)	0.149	0.362
Sphingomyelin C16:0	SM C16:0	67.2 ± 13.6	-0.13	(-0.32;0.06)	0.170	0.473	68.4 ± 12.6	ref.	66.1 ± 12.4	-0.17	(-0.48;0.14)	0.292	0.756	66.5 ± 15.7	-0.21	(-0.51;0.09)	0.173	0.407
Sphingomyelin C16:1	SM C16:1	9.5 ± 2.2	-0.12	(-0.30;0.06)	0.177	0.480	9.5 ± 2.2	ref.	9.6 ± 2.3	-0.06	(-0.35;0.24)	0.706	0.917	9.2 ± 2.1	-0.30	(-0.58;-0.02)	**0.038**	0.160
Sphingomyelin C18:0	SM C18:0	12.0 ± 3.3	0.04	(-0.16;0.23)	0.731	0.867	11.6 ± 2.8	ref.	12.0 ± 3.2	0.06	(-0.27;0.39)	0.719	0.917	12.5 ± 3.9	0.09	(-0.23;0.40)	0.592	0.794
Sphingomyelin C18:1	SM C18:1	5.7 ± 1.6	0.02	(-0.17;0.20)	0.854	0.938	5.5 ± 1.5	ref.	5.8 ± 1.8	0.05	(-0.26;0.35)	0.769	0.917	5.8 ± 1.7	-0.03	(-0.32;0.26)	0.835	0.933
Sphingomyelin C20:2	SM C20:2	0.2 ± 0.1	0.05	(-0.14;0.24)	0.612	0.820	0.15 ± 0.05	ref.	0.1 ± 0.1	-0.19	(-0.50;0.13)	0.241	0.741	0.15 ± 0.05	0.01	(-0.30;0.31)	0.971	0.990
Sphingomyelin C24:0	SM C24:0	6.1 ± 1.4	-0.26	(-0.46;-0.06)	**0.011**	0.137	6.4 ± 1.4	ref.	6.2 ± 1.4	-0.08	(-0.40;0.25)	0.655	0.915	5.7 ± 1.3	-0.39	(-0.71;-0.08)	**0.015**	0.110
Sphingomyelin C24:1	SM C24:1	13.9 ± 3.4	-0.08	(-0.28;0.13)	0.453	0.759	14.0 ± 3.1	ref.	13.7 ± 3.0	-0.04	(-0.37;0.30)	0.820	0.931	14.0 ± 4.2	-0.05	(-0.37;0.27)	0.756	0.881
Sphingomyelin C26:0	SM C26:0	0.04 ± 0.01	-0.03	(-0.25;0.18)	0.746	0.877	0.04 ± 0.01	ref.	0.04 ± 0.01	0.12	(-0.22;0.47)	0.482	0.869	0.04 ± 0.01	0.09	(-0.24;0.42)	0.583	0.794
Sphingomyelin C26:1	SM C26:1	0.10 ± 0.04	0.14	(-0.07;0.35)	0.206	0.514	0.10 ± 0.03	ref.	0.10 ± 0.03	0.33	(-0.02;0.67)	0.067	0.488	0.1 ± 0.1	0.32	(-0.01;0.65)	0.062	0.217
**HEXOSES**																		
Hexoses	H1	5574.0 ± 1481.3	0.07	(-0.15;0.28)	0.538	0.795	5336.7 ± 1103.8	ref.	5805.1 ± 1707.6	0.27	(-0.08;0.62)	0.132	0.591	5659.8 ± 1644.2	0.15	(-0.19;0.48)	0.389	0.686

			DHD15-index																	
			Continuous				Tertile 1		Tertile 2					Tertile 3						
**Metabolite**	**Abbreviation**	Mean ± SD [2]	β	95% CI [3]	p-value	p_{FDR} [4]	Mean ± SD [2]	β	Mean ± SD [2]	β	95% CI [3]	p-value	p_{FDR} [4]	Mean ± SD [2]	β	95% CI [3]	p-value	p_{FDR} [4]	p_{trend}	$p_{trend\ FDR}$ [4]
**ACYLCARNITINES**																				
Carnitine	C0	36.0 ± 7.3	0.04	(-0.06;0.15)	0.420	0.722	36.5 ± 8.3	ref.	34.7 ± 7.2	-0.15	(-0.51;0.21)	0.420	0.735	36.7 ± 6.2	0.18	(-0.19;0.54)	0.339	0.702	0.360	0.715
Acetylcarnitine	C2	6.0 ± 1.9	-0.08	(-0.19;0.02)	0.135	0.507	6.3 ± 2.3	ref.	5.7 ± 1.8	-0.36	(-0.72;-0.01)	**0.048**	0.380	5.8 ± 1.5	-0.23	(-0.59;0.12)	0.204	0.673	0.252	0.677
Propionylcarnitine	C3	0.5 ± 0.2	0.05	(-0.06;0.15)	0.385	0.722	0.5 ± 0.2	ref.	0.4 ± 0.1	-0.22	(-0.58;0.13)	0.218	0.522	0.5 ± 0.2	0.19	(-0.16;0.55)	0.295	0.702	0.329	0.711
Butyrylcarnitine	C4	0.2 ± 0.1	0.04	(-0.07;0.15)	0.460	0.734	0.2 ± 0.1	ref.	0.2 ± 0.1	-0.32	(-0.68;0.04)	0.087	0.433	0.2 ± 0.1	0.03	(-0.34;0.39)	0.885	0.941	0.937	0.979
Valerylcarnitine	C5	0.2 ± 0.1	0.04	(-0.06;0.14)	0.464	0.734	0.2 ± 0.1	ref.	0.2 ± 0.1	-0.07	(-0.41;0.27)	0.701	0.876	0.2 ± 0.1	0.14	(-0.20;0.48)	0.420	0.731	0.415	0.759
Decenoylcarnitine	C10:1	0.1 ± 0.1	0.01	(-0.10;0.12)	0.859	0.943	0.1 ± 0.1	ref.	0.12 ± 0.05	-0.10	(-0.46;0.26)	0.595	0.876	0.1 ± 0.1	0.05	(-0.31;0.42)	0.780	0.925	0.800	0.916
Tetradecenoylcarnitine	C14:1	0.05 ± 0.02	-0.12	(-0.23;-0.02)	**0.023**	0.259	0.06 ± 0.03	ref.	0.05 ± 0.03	-0.19	(-0.55;0.16)	0.286	0.607	0.05 ± 0.02	-0.35	(-0.70;0.01)	0.059	0.539	0.053	0.447
Tetradecadienylcarnitine	C14:2	0.02 ± 0.01	-0.10	(-0.20;0.01)	0.076	0.406	0.03 ± 0.01	ref.	0.02 ± 0.01	-0.33	(-0.68;0.03)	0.071	0.430	0.02 ± 0.01	-0.28	(-0.64;0.07)	0.121	0.578	0.106	0.555
Hexadecanoylcarnitine	C16	0.10 ± 0.03	-0.16	(-0.26;-0.05)	**0.003**	0.196	0.11 ± 0.03	ref.	0.11 ± 0.03	-0.16	(-0.52;0.19)	0.375	0.686	0.10 ± 0.02	-0.39	(-0.74;-0.03)	**0.035**	0.539	**0.034**	0.447
Octadecanoylcarnitine	C18	0.05 ± 0.01	-0.12	(-0.22;-0.01)	**0.030**	0.287	0.05 ± 0.01	ref.	0.05 ± 0.01	-0.19	(-0.54;0.17)	0.312	0.624	0.05 ± 0.01	-0.25	(-0.61;0.11)	0.169	0.624	0.150	0.598
Octadecenoylcarnitine	C18:1	0.09 ± 0.03	-0.14	(-0.25;-0.03)	**0.011**	0.196	0.08 ± 0.02	ref.	0.09 ± 0.04	0.08	(-0.29;0.44)	0.686	0.876	0.08 ± 0.03	-0.30	(-0.67;0.06)	0.108	0.578	0.106	0.555
Octadecadienylcarnitine	C18:2	0.03 ± 0.01	-0.08	(-0.19;0.03)	0.166	0.543	0.03 ± 0.01	ref.	0.04 ± 0.02	0.07	(-0.30;0.44)	0.727	0.878	0.03 ± 0.01	-0.12	(-0.49;0.25)	0.522	0.809	0.517	0.797
**AMINO ACIDS**																				
Alanine	Ala	382.8 ± 100.7	0.00	(-0.11;0.11)	0.998	0.998	380.0 ± 84.8	ref.	388.1 ± 125.5	-0.02	(-0.39;0.35)	0.924	0.967	380.3 ± 87.9	-0.04	(-0.41;0.33)	0.840	0.935	0.834	0.916
Arginine	Arg	81.3 ± 23.5	0.10	(-0.01;0.20)	0.082	0.415	79.1 ± 20.9	ref.	79.4 ± 28.1	-0.15	(-0.51;0.21)	0.422	0.735	85.5 ± 20.3	0.18	(-0.18;0.55)	0.323	0.702	0.320	0.702
Asparagine	Asn	43.5 ± 9.0	0.01	(-0.10;0.11)	0.909	0.964	43.0 ± 8.3	ref.	43.9 ± 9.4	0.01	(-0.35;0.36)	0.959	0.967	43.5 ± 9.5	-0.08	(-0.44;0.27)	0.645	0.872	0.609	0.815
Aspartate	Asp	11.8 ± 4.0	-0.07	(-0.18;0.04)	0.234	0.629	12.2 ± 3.9	ref.	11.9 ± 4.3	-0.12	(-0.49;0.25)	0.519	0.848	11.4 ± 3.7	-0.23	(-0.60;0.14)	0.227	0.682	0.238	0.677
Citrulline	Cit	36.5 ± 10.2	0.00	(-0.11;0.11)	0.971	0.983	35.3 ± 8.1	ref.	37.9 ± 11.7	0.04	(-0.33;0.41)	0.839	0.914	36.2 ± 10.5	-0.04	(-0.41;0.33)	0.826	0.935	0.827	0.916
Glutamine	Gln	696.5 ± 98.5	-0.01	(-0.12;0.09)	0.810	0.928	691.6 ± 108.0	ref.	699.0 ± 103.2	-0.03	(-0.38;0.32)	0.871	0.941	698.9 ± 83.9	0.00	(-0.35;0.35)	0.991	0.991	0.933	0.979
Glutamate	Glu	80.0 ± 29.7	-0.02	(-0.13;0.08)	0.671	0.852	85.2 ± 32.9	ref.	78.8 ± 27.4	-0.07	(-0.43;0.29)	0.700	0.876	76.0 ± 28.1	-0.19	(-0.56;0.17)	0.301	0.702	0.348	0.715
Glycine	Gly	215.6 ± 59.8	-0.05	(-0.15;0.05)	0.326	0.718	210.6 ± 67.4	ref.	228.9 ± 62.3	0.25	(-0.08;0.58)	0.136	0.493	207.3 ± 46.2	-0.07	(-0.41;0.26)	0.665	0.882	0.798	0.916
Histidine	His	71.1 ± 12.4	-0.02	(-0.12;0.09)	0.759	0.910	72.2 ± 11.4	ref.	68.5 ± 11.7	-0.34	(-0.69;0.02)	0.064	0.430	72.6 ± 13.8	-0.05	(-0.41;0.30)	0.774	0.925	0.750	0.916
Isoleucine	Ile	75.4 ± 23.9	0.07	(-0.04;0.17)	0.202	0.608	74.3 ± 20.5	ref.	72.3 ± 23.9	-0.11	(-0.46;0.24)	0.540	0.857	79.5 ± 26.8	0.16	(-0.19;0.51)	0.363	0.718	0.430	0.767
Leucine	Leu	133.8 ± 42.3	0.05	(-0.05;0.16)	0.315	0.718	133.1 ± 38.2	ref.	127.3 ± 40.4	-0.09	(-0.44;0.26)	0.619	0.876	141.1 ± 47.2	0.17	(-0.18;0.53)	0.340	0.702	0.365	0.715
Lysine	Lys	193.5 ± 41.8	0.04	(-0.06;0.15)	0.425	0.722	191 ± 37.9	ref.	190.5 ± 44.1	-0.10	(-0.45;0.26)	0.597	0.876	199.0 ± 43.2	0.12	(-0.23;0.48)	0.507	0.809	0.531	0.804
Methionine	Met	20.20 ± 5.8	0.04	(-0.06;0.15)	0.419	0.722	20.20 ± 5.2	ref.	19.4 ± 5.9	-0.18	(-0.54;0.17)	0.317	0.624	21.0 ± 6.3	0.08	(-0.28;0.43)	0.678	0.882	0.766	0.916
Ornithine	Orn	71.3 ± 24.6	-0.06	(-0.17;0.04)	0.238	0.629	70.4 ± 22.2	ref.	75.9 ± 29.7	0.07	(-0.30;0.43)	0.719	0.878	67.6 ± 20.5	-0.18	(-0.54;0.19)	0.340	0.702	0.310	0.692
Phenylalanine	Phe	62.8 ± 13.6	-0.03	(-0.13;0.07)	0.573	0.825	63.7 ± 13.4	ref.	60.5 ± 12.2	-0.34	(-0.69;0.01)	0.059	0.430	64.1 ± 15.1	-0.12	(-0.47;0.23)	0.504	0.809	0.419	0.759
Proline	Pro	242.6 ± 79.5	0.10	(0.00;0.20)	0.062	0.362	232.6 ± 77.2	ref.	237.1 ± 75.2	0.04	(-0.32;0.39)	0.832	0.914	258.1 ± 84.7	0.21	(-0.15;0.56)	0.259	0.702	0.241	0.677
Serine	Ser	93 ± 20.3	0.03	(-0.08;0.14)	0.573	0.825	92.0 ± 19.5	ref.	90.7 ± 21.3	-0.16	(-0.53;0.20)	0.379	0.686	96.4 ± 20.0	0.12	(-0.24;0.48)	0.518	0.809	0.581	0.804
Threonine	Thr	108.7 ± 27.7	0.01	(-0.10;0.12)	0.853	0.943	109.9 ± 28.7	ref.	107.3 ± 29.2	-0.11	(-0.47;0.25)	0.549	0.857	109.1 ± 25.4	-0.03	(-0.39;0.33)	0.856	0.935	0.792	0.916
Tryptophan	Trp	53.1 ± 11.0	0.04	(-0.06;0.15)	0.443	0.733	52.9 ± 11.4	ref.	51.2 ± 9.9	-0.08	(-0.44;0.28)	0.671	0.876	55.1 ± 11.4	0.20	(-0.16;0.56)	0.271	0.702	0.270	0.691
Tyrosine	Tyr	74.8 ± 24.1	0.03	(-0.08;0.13)	0.627	0.852	73.8 ± 22.0	ref.	72.0 ± 22.6	-0.19	(-0.55;0.18)	0.316	0.624	78.4 ± 27.3	0.06	(-0.30;0.43)	0.733	0.909	0.781	0.916
Valine	Val	242.1 ± 64.0	0.03	(-0.07;0.14)	0.521	0.793	243 ± 57.5	ref.	230.8 ± 60.2	-0.15	(-0.50;0.20)	0.415	0.735	252.4 ± 72.5	0.13	(-0.22;0.48)	0.468	0.775	0.495	0.794
**BIOGENIC AMINES**																				
Creatinine	Creatinine	74.5 ± 17.4	0.00	(-0.08;0.09)	0.914	0.964	75.3 ± 15.5	ref.	72.7 ± 16.8	0.04	(-0.25;0.34)	0.765	0.900	75.4 ± 19.6	0.15	(-0.15;0.44)	0.328	0.702	0.296	0.692
Kynurenine	Kynurenine	2.7 ± 0.7	-0.02	(-0.12;0.08)	0.674	0.852	2.6 ± 0.7	ref.	2.8 ± 0.8	0.23	(-0.11;0.57)	0.192	0.520	2.7 ± 0.7	0.14	(-0.20;0.49)	0.413	0.731	0.462	0.793
Sarcosine	Sarcosine	4.4 ± 1.1	-0.10	(-0.21;0.00)	0.062	0.362	4.6 ± 1.0	ref.	4.4 ± 1.2	-0.05	(-0.41;0.31)	0.793	0.903	4.2 ± 1.1	-0.37	(-0.73;-0.01)	**0.045**	0.539	0.052	0.447
Serotonin	Serotonin	0.8 ± 0.6	-0.05	(-0.16;0.05)	0.336	0.718	0.8 ± 0.6	ref.	0.7 ± 0.5	-0.32	(-0.68;0.04)	0.084	0.430	0.7 ± 0.6	-0.23	(-0.59;0.13)	0.216	0.674	0.181	0.623
Taurine	Taurine	120.9 ± 49.4	-0.03	(-0.14;0.07)	0.542	0.815	123.7 ± 51.4	ref.	117.2 ± 50.3	-0.25	(-0.62;0.11)	0.177	0.520	121.7 ± 46.9	-0.12	(-0.48;0.25)	0.538	0.811	0.548	0.804
Trans 4-Hydroxyproline	t4-OH-Pro	10.2 ± 5.0	-0.04	(-0.14;0.07)	0.507	0.790	10.9 ± 4.2	ref.	9.3 ± 3.9	-0.32	(-0.67;0.03)	0.078	0.430	10.4 ± 6.4	-0.20	(-0.55;0.16)	0.282	0.702	0.259	0.681
Asymmetric dimethylarginine	ADMA	0.5 ± 0.1	-0.06	(-0.16;0.04)	0.239	0.629	0.5 ± 0.1	ref.	0.5 ± 0.1	0.04	(-0.28;0.37)	0.794	0.903	0.4 ± 0.1	-0.17	(-0.50;0.16)	0.316	0.702	0.385	0.737
Symmetric dimethylarginine	SDMA	0.5 ± 0.1	-0.08	(-0.18;0.02)	0.110	0.462	0.5 ± 0.1	ref.	0.5 ± 0.1	0.04	(-0.30;0.37)	0.832	0.914	0.4 ± 0.1	-0.22	(-0.56;0.12)	0.206	0.673	0.232	0.677
**GLYCEROPHOSPHOLIPIDS**																				
Lysophosphatidylcholine (acyl) C16:0	lysoPC a C16:0	90.4 ± 20.1	-0.03	(-0.14;0.08)	0.650	0.852	87.8 ± 17.8	ref.	92.6 ± 23.1	0.24	(-0.13;0.61)	0.205	0.520	90.9 ± 19.0	0.15	(-0.22;0.52)	0.435	0.747	0.348	0.715
Lysophosphatidylcholine (acyl) C16:1	lysoPC a C16:1	2.6 ± 0.9	-0.14	(-0.25;-0.03)	**0.013**	0.196	2.6 ± 1.0	ref.	2.7 ± 1.1	0.01	(-0.36;0.38)	0.970	0.970	2.4 ± 0.7	-0.16	(-0.53;0.22)	0.408	0.731	0.483	0.794
Lysophosphatidylcholine (acyl) C17:0	lysoPC a C17:0	1.6 ± 0.5	0.05	(-0.06;0.16)	0.352	0.722	1.4 ± 0.5	ref.	1.7 ± 0.6	0.46	(0.11;0.81)	**0.011**	0.207	1.6 ± 0.4	0.35	(0.00;0.71)	**0.050**	0.539	**0.032**	0.447
Lysophosphatidylcholine (acyl) C18:0	lysoPC a C18:0	26.4 ± 6.8	0.01	(-0.10;0.11)	0.905	0.964	25.1 ± 5.8	ref.	27.4 ± 8.6	0.22	(-0.14;0.58)	0.238	0.549	26.6 ± 5.5	0.16	(-0.20;0.52)	0.389	0.731	0.303	0.692
Lysophosphatidylcholine (acyl) C18:1	lysoPC a C18:1	17.9 ± 5.4	-0.05	(-0.15;0.06)	0.387	0.722	17.5 ± 5.2	ref.	18.7 ± 6.0	0.23	(-0.13;0.60)	0.206	0.520	17.7 ± 4.8	0.08	(-0.29;0.44)	0.672	0.882	0.586	0.804
Lysophosphatidylcholine (acyl) C18:2	lysoPC a C18:2	29.2 ± 10.4	0.03	(-0.07;0.14)	0.520	0.793	28.2 ± 10.0	ref.	29.1 ± 11.5	0.05	(-0.30;0.40)	0.781	0.903	30.5 ± 9.6	0.19	(-0.16;0.54)	0.293	0.702	0.253	0.677
Lysophosphatidylcholine (acyl) C20:3	lysoPC a C20:3	2.0 ± 0.7	-0.08	(-0.18;0.03)	0.155	0.531	2.0 ± 0.7	ref.	2.1 ± 0.7	0.14	(-0.22;0.50)	0.441	0.758	2.0 ± 0.6	-0.03	(-0.40;0.33)	0.857	0.935	0.985	0.985
Lysophosphatidylcholine (acyl) C20:4	lysoPC a C20:4	5.9 ± 1.7	-0.05	(-0.16;0.06)	0.341	0.718	5.8 ± 1.7	ref.	6.1 ± 2.0	0.23	(-0.13;0.60)	0.210	0.520	5.8 ± 1.4	0.04	(-0.32;0.41)	0.819	0.935	0.701	0.897
Lysophosphatidylcholine (acyl) C24:0	lysoPC a C24:0	0.14 ± 0.03	0.03	(-0.07;0.13)	0.579	0.825	0.13 ± 0.03	ref.	0.14 ± 0.04	0.22	(-0.11;0.55)	0.195	0.520	0.14 ± 0.03	0.20	(-0.13;0.53)	0.242	0.690	0.189	0.634
Lysophosphatidylcholine (acyl) C28:1	lysoPC a C28:1	0.3 ± 0.1	-0.02	(-0.11;0.07)	0.669	0.852	0.3 ± 0.1	ref.	0.3 ± 0.1	0.31	(0.01;0.60)	**0.044**	0.367	0.3 ± 0.1	0.08	(-0.21;0.38)	0.591	0.833	0.498	0.794
Phosphatidylcholine (diacyl) C28:1	PC aa C28:1	3.0 ± 0.8	-0.02	(-0.12;0.08)	0.747	0.910	2.8 ± 0.7	ref.	3.1 ± 0.8	-0.02	(-0.36;0.32)	0.908	0.958	3.1 ± 0.7	-0.01	(-0.35;0.33)	0.968	0.983	0.902	0.966
Phosphatidylcholine (diacyl) C30:0	PC aa C30:0	4.0 ± 1.3	-0.04	(-0.15;0.06)	0.425	0.722	4.0 ± 1.4	ref.	4.0 ± 1.3	-0.19	(-0.55;0.18)	0.312	0.624	4.1 ± 1.3	-0.10	(-0.47;0.26)	0.577	0.832	0.579	0.804
Phosphatidylcholine (diacyl) C32:0	PC aa C32:0	26.6 ± 5.5	-0.03	(-0.13;0.07)	0.573	0.825	26.9 ± 6.3	ref.	25.9 ± 5.1	-0.36	(-0.71;-0.01)	**0.044**	0.367	27.1 ± 4.9	-0.09	(-0.44;0.26)	0.632	0.865	0.588	0.804
Phosphatidylcholine (diacyl) C32:1	PC aa C32:1	32.0 ± 16.2	-0.15	(-0.25;-0.04)	**0.008**	0.196	35.7 ± 19.7	ref.	31.1 ± 16	-0.40	(-0.76;-0.03)	**0.035**	0.330	29.2 ± 11.1	-0.39	(-0.75;-0.02)	**0.039**	0.539	**0.037**	0.447

Metabolite	Abbreviation	Mean ± SD [2]	Continuous β	95% CI [3]	*p*-value	p_{FDR} [4]	Tertile 1 Mean ± SD [2]	β	Tertile 2 Mean ± SD [2]	β	95% CI [3]	*p*-value	p_{FDR} [4]	Tertile 3 Mean ± SD [2]	β	95% CI [3]	*p*-value	p_{FDR} [4]	p_{trend}	$p_{trend\ FDR}$ [4]
Phosphatidylcholine (diacyl) C32:2	PC aa C32:2	8.3 ± 3.1	-0.04	(-0.15;0.06)	0.416	0.722	8.3 ± 3.1	ref.	8.2 ± 3.2	-0.27	(-0.63;0.09)	0.148	0.505	8.5 ± 3.0	-0.16	(-0.52;0.21)	0.402	0.731	0.503	0.794
Phosphatidylcholine (diacyl) C32:3	PC aa C32:3	0.9 ± 0.2	-0.04	(-0.13;0.06)	0.466	0.734	0.9 ± 0.2	ref.	0.9 ± 0.3	-0.19	(-0.52;0.14)	0.265	0.581	0.9 ± 0.2	-0.14	(-0.47;0.19)	0.410	0.731	0.544	0.804
Phosphatidylcholine (diacyl) C34:1	PC aa C34:1	365.4 ± 82.8	-0.12	(-0.23;-0.02)	**0.023**	0.259	376.6 ± 89.6	ref.	361.7 ± 83.5	-0.30	(-0.66;0.06)	0.108	0.481	357.9 ± 74.8	-0.30	(-0.66;0.06)	0.106	0.578	0.103	0.555
Phosphatidylcholine (diacyl) C34:2	PC aa C34:2	739.8 ± 128.1	-0.02	(-0.13;0.08)	0.656	0.852	751.2 ± 126.7	ref.	715.9 ± 132.2	-0.48	(-0.85;-0.12)	**0.010**	0.207	752.2 ± 123.8	-0.19	(-0.56;0.17)	0.297	0.702	0.308	0.692
Phosphatidylcholine (diacyl) C34:3	PC aa C34:3	30.1 ± 10.2	-0.10	(-0.21;0.00)	0.061	0.362	30.6 ± 9.2	ref.	30.0 ± 12.1	-0.40	(-0.76;-0.05)	**0.027**	0.302	29.8 ± 9.1	-0.32	(-0.67;0.04)	0.079	0.558	0.089	0.555
Phosphatidylcholine (diacyl) C34:4	PC aa C34:4	3.1 ± 1.2	-0.09	(-0.20;0.02)	0.096	0.429	3.2 ± 1.3	ref.	3.1 ± 1.2	-0.27	(-0.63;0.08)	0.133	0.493	3.0 ± 1.1	-0.26	(-0.62;0.10)	0.159	0.607	0.218	0.664
Phosphatidylcholine (diacyl) C36:0	PC aa C36:0	3.2 ± 1.0	0.06	(-0.04;0.16)	0.248	0.637	3.2 ± 1.1	ref.	3.0 ± 0.9	-0.30	(-0.65;0.05)	0.091	0.435	3.4 ± 1.0	0.11	(-0.24;0.46)	0.528	0.809	0.498	0.794
Phosphatidylcholine (diacyl) C36:1	PC aa C36:1	62.9 ± 17.8	-0.08	(-0.18;0.03)	0.142	0.507	63.1 ± 15.9	ref.	63.1 ± 20	-0.22	(-0.56;0.13)	0.215	0.522	62.6 ± 17.4	-0.16	(-0.51;0.19)	0.364	0.718	0.368	0.715
Phosphatidylcholine (diacyl) C36:2	PC aa C36:2	384.7 ± 79.7	-0.01	(-0.11;0.09)	0.866	0.943	385.0 ± 76.2	ref.	376.8 ± 87.4	-0.39	(-0.73;-0.05)	**0.027**	0.302	392.4 ± 75.4	-0.14	(-0.49;0.20)	0.416	0.731	0.435	0.767
Phosphatidylcholine (diacyl) C36:3	PC aa C36:3	192.6 ± 43.8	-0.11	(-0.21;-0.01)	**0.041**	0.362	193.8 ± 44.1	ref.	192.7 ± 45.6	-0.28	(-0.63;0.07)	0.113	0.491	191.2 ± 42.3	-0.26	(-0.61;0.08)	0.138	0.578	0.165	0.598
Phosphatidylcholine (diacyl) C36:4	PC aa C36:4	291.5 ± 72.0	-0.10	(-0.20;0.00)	0.060	0.362	300.3 ± 79.3	ref.	287.2 ± 72.3	-0.32	(-0.67;0.03)	0.074	0.430	286.9 ± 64.0	-0.28	(-0.63;0.07)	0.113	0.578	0.145	0.598
Phosphatidylcholine (diacyl) C36:5	PC aa C36:5	45.8 ± 27.8	0.01	(-0.09;0.12)	0.787	0.910	48.5 ± 36.9	ref.	43.3 ± 20.1	-0.07	(-0.44;0.29)	0.694	0.876	45.4 ± 23.7	0.04	(-0.32;0.41)	0.827	0.935	0.833	0.916
Phosphatidylcholine (diacyl) C36:6	PC aa C36:6	1.5 ± 0.6	0.05	(-0.06;0.15)	0.392	0.722	1.5 ± 0.7	ref.	1.5 ± 0.6	-0.09	(-0.44;0.27)	0.629	0.876	1.6 ± 0.7	0.17	(-0.19;0.53)	0.347	0.705	0.280	0.691
Phosphatidylcholine (diacyl) C38:0	PC aa C38:0	2.9 ± 0.9	0.09	(-0.02;0.19)	0.101	0.437	2.7 ± 0.9	ref.	2.8 ± 0.8	-0.08	(-0.42;0.26)	0.652	0.876	3.1 ± 0.9	0.24	(-0.10;0.58)	0.174	0.624	0.171	0.603
Phosphatidylcholine (diacyl) C38:3	PC aa C38:3	53.2 ± 13.6	-0.12	(-0.21;-0.02)	**0.019**	0.252	53.1 ± 12.9	ref.	53.9 ± 14.1	-0.21	(-0.54;0.11)	0.202	0.520	52.6 ± 14	-0.25	(-0.58;0.08)	0.132	0.578	0.158	0.598
Phosphatidylcholine (diacyl) C38:4	PC aa C38:4	131.5 ± 35.0	-0.09	(-0.19;0.00)	0.056	0.362	133.4 ± 38.7	ref.	132.2 ± 34.1	-0.21	(-0.54;0.12)	0.209	0.520	129.1 ± 32.2	-0.25	(-0.58;0.08)	0.133	0.578	0.165	0.598
Phosphatidylcholine (diacyl) C38:5	PC aa C38:5	66.2 ± 19.0	-0.07	(-0.17;0.04)	0.208	0.608	67.6 ± 21.6	ref.	65.4 ± 18.3	-0.24	(-0.58;0.10)	0.176	0.520	65.7 ± 17.2	-0.15	(-0.50;0.19)	0.379	0.730	0.409	0.759
Phosphatidylcholine (diacyl) C38:6	PC aa C38:6	101.1 ± 33.6	0.10	(0.00;0.21)	0.056	0.362	96.0 ± 34.9	ref.	97.1 ± 29.3	-0.07	(-0.42;0.27)	0.675	0.876	110.3 ± 34.9	0.35	(0.00;0.70)	**0.049**	0.539	**0.043**	0.447
Phosphatidylcholine (diacyl) C40:2	PC aa C40:2	0.3 ± 0.1	0.05	(-0.05;0.15)	0.328	0.718	0.3 ± 0.1	ref.	0.3 ± 0.1	-0.23	(-0.57;0.12)	0.195	0.520	0.3 ± 0.1	0.10	(-0.24;0.45)	0.561	0.817	0.596	0.807
Phosphatidylcholine (diacyl) C40:3	PC aa C40:3	0.4 ± 0.1	-0.02	(-0.12;0.08)	0.719	0.900	0.4 ± 0.2	ref.	0.4 ± 0.1	-0.22	(-0.56;0.12)	0.201	0.520	0.4 ± 0.1	-0.03	(-0.37;0.31)	0.847	0.935	0.802	0.916
Phosphatidylcholine (diacyl) C40:4	PC aa C40:4	3.0 ± 0.8	-0.17	(-0.28;-0.07)	**0.001**	0.153	3.2 ± 1.0	ref.	3.0 ± 0.8	-0.40	(-0.75;-0.04)	**0.029**	0.302	2.9 ± 0.7	-0.50	(-0.85;-0.14)	**0.007**	0.294	**0.009**	0.365
Phosphatidylcholine (diacyl) C40:5	PC aa C40:5	8.3 ± 2.4	-0.14	(-0.24;-0.03)	**0.010**	0.196	8.6 ± 2.7	ref.	8.3 ± 2.3	-0.29	(-0.64;0.06)	0.106	0.481	8.0 ± 2.3	-0.38	(-0.73;-0.03)	**0.035**	0.539	**0.036**	0.447
Phosphatidylcholine (diacyl) C40:6	PC aa C40:6	28.3 ± 10.2	0.08	(-0.02;0.18)	0.118	0.469	27 ± 10.9	ref.	27.4 ± 8.6	-0.05	(-0.40;0.29)	0.755	0.895	30.5 ± 10.7	0.28	(-0.06;0.63)	0.109	0.578	0.108	0.555
Phosphatidylcholine (diacyl) C42:0	PC aa C42:0	0.3 ± 0.1	0.14	(0.04;0.24)	**0.006**	0.196	0.3 ± 0.1	ref.	0.3 ± 0.1	0.11	(-0.22;0.44)	0.526	0.849	0.4 ± 0.1	0.43	(0.09;0.76)	**0.013**	0.445	**0.011**	0.365
Phosphatidylcholine (diacyl) C42:1	PC aa C42:1	0.18 ± 0.05	0.13	(0.04;0.23)	**0.008**	0.196	0.16 ± 0.05	ref.	0.18 ± 0.05	0.26	(-0.07;0.59)	0.131	0.493	0.19 ± 0.05	0.47	(0.14;0.81)	**0.006**	0.294	**0.005**	0.365
Phosphatidylcholine (diacyl) C42:2	PC aa C42:2	0.16 ± 0.04	0.04	(-0.06;0.15)	0.397	0.722	0.16 ± 0.05	ref.	0.15 ± 0.04	-0.27	(-0.61;0.08)	0.131	0.493	0.17 ± 0.04	0.18	(-0.16;0.53)	0.305	0.702	0.284	0.691
Phosphatidylcholine (diacyl) C42:4	PC aa C42:4	0.10 ± 0.02	-0.06	(-0.17;0.05)	0.295	0.718	0.11 ± 0.03	ref.	0.10 ± 0.02	-0.43	(-0.79;-0.07)	**0.022**	0.302	0.11 ± 0.02	-0.12	(-0.48;0.25)	0.531	0.809	0.558	0.804
Phosphatidylcholine (diacyl) C42:5	PC aa C42:5	0.3 ± 0.1	0.00	(-0.10;0.11)	0.955	0.983	0.3 ± 0.2	ref.	0.2 ± 0.1	-0.26	(-0.62;0.10)	0.157	0.514	0.3 ± 0.1	-0.06	(-0.42;0.30)	0.749	0.910	0.698	0.897
Phosphatidylcholine (diacyl) C42:6	PC aa C42:6	0.4 ± 0.1	-0.05	(-0.15;0.06)	0.384	0.722	0.4 ± 0.2	ref.	0.4 ± 0.1	-0.27	(-0.62;0.09)	0.145	0.505	0.4 ± 0.1	-0.10	(-0.46;0.26)	0.586	0.833	0.583	0.804
Phosphatidylcholine (acyl-alkyl) C30:0	PC ae C30:0	0.3 ± 0.1	-0.02	(-0.13;0.08)	0.660	0.852	0.3 ± 0.1	ref.	0.3 ± 0.1	-0.01	(-0.36;0.34)	0.951	0.967	0.3 ± 0.1	0.01	(-0.34;0.36)	0.948	0.977	0.883	0.961
Phosphatidylcholine (acyl-alkyl) C30:2	PC ae C30:2	0.08 ± 0.02	0.01	(-0.08;0.10)	0.759	0.910	0.08 ± 0.02	ref.	0.09 ± 0.02	0.22	(-0.08;0.53)	0.151	0.505	0.09 ± 0.02	0.12	(-0.19;0.42)	0.463	0.775	0.350	0.715
Phosphatidylcholine (acyl-alkyl) C32:1	PC ae C32:1	5.3 ± 1.2	-0.05	(-0.15;0.05)	0.343	0.718	5.2 ± 1.2	ref.	5.2 ± 1.3	-0.31	(-0.66;0.04)	0.080	0.430	5.3 ± 1.2	-0.16	(-0.50;0.19)	0.381	0.730	0.414	0.759
Phosphatidylcholine (acyl-alkyl) C32:2	PC ae C32:2	1.4 ± 0.4	-0.07	(-0.17;0.03)	0.159	0.531	1.3 ± 0.4	ref.	1.4 ± 0.4	-0.21	(-0.54;0.13)	0.226	0.530	1.4 ± 0.3	-0.17	(-0.51;0.16)	0.310	0.702	0.355	0.715
Phosphatidylcholine (acyl-alkyl) C34:0	PC ae C34:0	2.5 ± 0.6	-0.03	(-0.13;0.08)	0.636	0.852	2.4 ± 0.6	ref.	2.4 ± 0.7	-0.20	(-0.55;0.15)	0.254	0.568	2.5 ± 0.6	-0.02	(-0.37;0.33)	0.926	0.969	0.943	0.979
Phosphatidylcholine (acyl-alkyl) C34:1	PC ae C34:1	18.0 ± 3.9	-0.05	(-0.15;0.05)	0.324	0.718	17.5 ± 3.3	ref.	18.4 ± 4.7	-0.09	(-0.43;0.25)	0.599	0.876	18.2 ± 3.6	-0.06	(-0.40;0.28)	0.721	0.909	0.739	0.916
Phosphatidylcholine (acyl-alkyl) C34:2	PC ae C34:2	20.2 ± 5.3	-0.07	(-0.17;0.03)	0.189	0.603	20.4 ± 5.0	ref.	19.7 ± 5.9	-0.48	(-0.81;-0.14)	**0.006**	0.207	20.6 ± 5.0	-0.27	(-0.61;0.07)	0.123	0.578	0.146	0.598
Phosphatidylcholine (acyl-alkyl) C34:3	PC ae C34:3	13.1 ± 4.0	-0.01	(-0.11;0.09)	0.851	0.943	13.0 ± 3.3	ref.	12.8 ± 4.5	-0.39	(-0.73;-0.05)	**0.025**	0.302	13.6 ± 4.0	-0.14	(-0.48;0.21)	0.440	0.747	0.513	0.797
Phosphatidylcholine (acyl-alkyl) C36:0	PC ae C36:0	1.2 ± 0.3	-0.09	(-0.20;0.01)	0.089	0.424	1.2 ± 0.4	ref.	1.1 ± 0.3	-0.41	(-0.77;-0.05)	**0.027**	0.302	1.1 ± 0.3	-0.29	(-0.65;0.07)	0.111	0.578	0.090	0.555
Phosphatidylcholine (acyl-alkyl) C36:1	PC ae C36:1	11.5 ± 2.7	-0.01	(-0.11;0.09)	0.786	0.910	10.9 ± 2.2	ref.	11.8 ± 3.2	0.08	(-0.26;0.42)	0.648	0.876	11.7 ± 2.5	0.08	(-0.26;0.41)	0.660	0.882	0.579	0.804
Phosphatidylcholine (acyl-alkyl) C36:2	PC ae C36:2	21.7 ± 5.4	0.04	(-0.06;0.14)	0.404	0.722	20.6 ± 4.8	ref.	22.0 ± 6.4	-0.08	(-0.41;0.25)	0.637	0.876	22.6 ± 4.7	0.11	(-0.22;0.44)	0.525	0.809	0.448	0.780
Phosphatidylcholine (acyl-alkyl) C36:3	PC ae C36:3	11.4 ± 2.8	-0.09	(-0.19;0.01)	0.075	0.406	11.5 ± 2.6	ref.	11.2 ± 3.2	-0.45	(-0.79;-0.12)	**0.008**	0.207	11.4 ± 2.7	-0.32	(-0.66;0.02)	0.063	0.539	0.082	0.555
Phosphatidylcholine (acyl-alkyl) C36:4	PC ae C36:4	26.3 ± 6.4	-0.14	(-0.24;-0.03)	**0.012**	0.196	27.5 ± 6.2	ref.	25.5 ± 6.4	-0.59	(-0.94;-0.24)	**0.001**	0.147	25.9 ± 6.4	-0.50	(-0.85;-0.15)	**0.006**	0.294	**0.009**	0.365
Phosphatidylcholine (acyl-alkyl) C36:5	PC ae C36:5	18.3 ± 5.0	-0.08	(-0.18;0.03)	0.144	0.507	19.1 ± 5.3	ref.	17.6 ± 4.7	-0.51	(-0.85;-0.17)	**0.004**	0.207	18.3 ± 4.8	-0.32	(-0.66;0.02)	0.067	0.539	0.099	0.555
Phosphatidylcholine (acyl-alkyl) C38:0	PC ae C38:0	2.5 ± 0.8	0.06	(-0.05;0.16)	0.280	0.695	2.4 ± 0.9	ref.	2.5 ± 0.8	-0.02	(-0.37;0.32)	0.889	0.953	2.6 ± 0.8	0.25	(-0.09;0.60)	0.152	0.607	0.120	0.594
Phosphatidylcholine (acyl-alkyl) C38:2	PC ae C38:2	2.2 ± 0.6	0.00	(-0.10;0.10)	0.961	0.983	2.1 ± 0.5	ref.	2.2 ± 0.7	-0.12	(-0.46;0.23)	0.506	0.838	2.2 ± 0.5	-0.01	(-0.36;0.33)	0.933	0.969	0.970	0.985
Phosphatidylcholine (acyl-alkyl) C38:3	PC ae C38:3	4.8 ± 1.1	-0.06	(-0.16;0.04)	0.223	0.629	4.6 ± 1.0	ref.	4.9 ± 1.3	-0.01	(-0.34;0.32)	0.941	0.967	4.8 ± 1.0	-0.07	(-0.40;0.26)	0.691	0.882	0.819	0.916
Phosphatidylcholine (acyl-alkyl) C38:4	PC ae C38:4	17.2 ± 3.6	-0.09	(-0.19;0.01)	0.095	0.429	17 ± 3.3	ref.	17.4 ± 3.9	-0.23	(-0.57;0.12)	0.195	0.520	17.2 ± 3.7	-0.26	(-0.61;0.08)	0.138	0.578	0.211	0.656
Phosphatidylcholine (acyl-alkyl) C38:5	PC ae C38:5	21.9 ± 4.7	-0.11	(-0.21;-0.01)	**0.028**	0.287	22.5 ± 4.7	ref.	21.6 ± 4.6	-0.47	(-0.81;-0.13)	**0.007**	0.207	21.8 ± 4.7	-0.40	(-0.74;-0.06)	**0.022**	0.539	**0.034**	0.447
Phosphatidylcholine (acyl-alkyl) C38:6	PC ae C38:6	9.2 ± 2.6	0.01	(-0.09;0.12)	0.775	0.910	9.2 ± 2.9	ref.	8.9 ± 2.4	-0.30	(-0.64;0.04)	0.082	0.430	9.5 ± 2.5	-0.01	(-0.35;0.33)	0.960	0.982	0.983	0.985
Phosphatidylcholine (acyl-alkyl) C40:1	PC ae C40:1	1.1 ± 0.3	0.05	(-0.06;0.15)	0.399	0.722	1.1 ± 0.3	ref.	1.1 ± 0.3	-0.10	(-0.45;0.25)	0.585	0.876	1.2 ± 0.3	0.22	(-0.14;0.57)	0.234	0.682	0.202	0.645
Phosphatidylcholine (acyl-alkyl) C40:2	PC ae C40:2	1.6 ± 0.4	0.02	(-0.08;0.13)	0.632	0.852	1.5 ± 0.4	ref.	1.6 ± 0.4	0.07	(-0.27;0.41)	0.697	0.876	1.6 ± 0.4	0.10	(-0.24;0.45)	0.553	0.815	0.497	0.794
Phosphatidylcholine (acyl-alkyl) C40:3	PC ae C40:3	0.8 ± 0.2	-0.02	(-0.12;0.07)	0.642	0.852	0.8 ± 0.2	ref.	0.9 ± 0.2	-0.07	(-0.39;0.26)	0.689	0.876	0.9 ± 0.2	0.00	(-0.33;0.32)	0.987	0.991	0.891	0.962
Phosphatidylcholine (acyl-alkyl) C40:4	PC ae C40:4	2.2 ± 0.4	-0.04	(-0.14;0.06)	0.461	0.734	2.2 ± 0.4	ref.	2.2 ± 0.5	-0.18	(-0.52;0.16)	0.298	0.624	2.3 ± 0.5	-0.07	(-0.41;0.27)	0.691	0.882	0.784	0.916
Phosphatidylcholine (acyl-alkyl) C40:5	PC ae C40:5	3.4 ± 0.7	-0.01	(-0.11;0.09)	0.784	0.910	3.3 ± 0.6	ref.	3.4 ± 0.8	-0.18	(-0.52;0.15)	0.284	0.607	3.4 ± 0.6	-0.03	(-0.37;0.31)	0.869	0.935	0.957	0.985
Phosphatidylcholine (acyl-alkyl) C40:6	PC ae C40:6	4.8 ± 1.2	0.10	(0.00;0.20)	**0.047**	0.362	4.4 ± 1.2	ref.	4.8 ± 1.3	0.04	(-0.29;0.38)	0.800	0.903	5.1 ± 1.1	0.32	(-0.02;0.66)	0.064	0.539	**0.049**	0.447
Phosphatidylcholine (acyl-alkyl) C42:1	PC ae C42:1	0.3 ± 0.1	-0.07	(-0.17;0.03)	0.201	0.608	0.3 ± 0.1	ref.	0.3 ± 0.1	-0.17	(-0.51;0.17)	0.339	0.643	0.3 ± 0.1	-0.09	(-0.43;0.26)	0.620	0.857	0.758	0.916
Phosphatidylcholine (acyl-alkyl) C42:2	PC ae C42:2	0.4 ± 0.1	0.04	(-0.06;0.14)	0.423	0.722	0.4 ± 0.1	ref.	0.4 ± 0.1	-0.10	(-0.43;0.23)	0.550	0.857	0.4 ± 0.1	0.16	(-0.17;0.49)	0.336	0.702	0.301	0.692
Phosphatidylcholine (acyl-alkyl) C42:3	PC ae C42:3	0.6 ± 0.1	0.09	(-0.01;0.19)	0.0837	0.415	0.5 ± 0.1	ref.	0.5 ± 0.1	-0.02	(-0.36;0.31)	0.8990	0.956	0.6 ± 0.1	0.35	(0.02;0.69)	**0.0415**	0.539	**0.034**	0.447
Phosphatidylcholine (acyl-alkyl) C42:4	PC ae C42:4	0.7 ± 0.2	0.04	(-0.06;0.15)	0.389	0.722	0.7 ± 0.1	ref.	0.7 ± 0.2	-0.04	(-0.39;0.30)	0.807	0.903	0.7 ± 0.2	0.18	(-0.17;0.52)	0.321	0.702	0.281	0.691
Phosphatidylcholine (acyl-alkyl) C42:5	PC ae C42:5	1.5 ± 0.3	0.04	(-0.06;0.14)	0.438	0.733	1.5 ± 0.3	ref.	1.6 ± 0.3	0.12	(-0.23;0.47)	0.504	0.838	1.6 ± 0.3	0.18	(-0.17;0.53)	0.304	0.702	0.246	0.677
Phosphatidylcholine (acyl-alkyl) C44:3	PC ae C44:3	0.07 ± 0.02	0.00	(-0.10;0.10)	0.950	0.983	0.07 ± 0.02	ref.	0.07 ± 0.02	-0.16	(-0.49;0.18)	0.358	0.667	0.07 ± 0.02	0.03	(-0.31;0.37)	0.865	0.935	0.828	0.916
Phosphatidylcholine (acyl-alkyl) C44:4	PC ae C44:4	0.2 ± 0.1	0.08	(-0.03;0.18)	0.142	0.507	0.2 ± 0.1	ref.	0.2 ± 0.1	-0.07	(-0.41;0.28)	0.706	0.876	0.2 ± 0.1	0.24	(-0.11;0.59)	0.177	0.624	0.151	0.598
Phosphatidylcholine (acyl-alkyl) C44:5	PC ae C44:5	1.0 ± 0.3	0.02	(-0.09;0.12)	0.775	0.910	1.0 ± 0.3	ref.	1.0 ± 0.3	0.06	(-0.29;0.42)	0.722	0.878	1.0 ± 0.3	0.06	(-0.30;0.42)	0.744	0.910	0.674	0.886
Phosphatidylcholine (acyl-alkyl) C44:6	PC ae C44:6	0.8 ± 0.2	0.10	(0.00;0.20)	0.053	0.362	0.7 ± 0.2	ref.	0.8 ± 0.2	0.07	(-0.27;0.41)	0.678	0.876	0.8 ± 0.2	0.32	(-0.02;0.66)	0.068	0.539	0.053	0.447

			Continuous				Tertile 1		Tertile 2					Tertile 3						
**Metabolite**	**Abbreviation**	Mean ± SD [2]	β	95% CI [3]	p-value	p_{FDR} [4]	Mean ± SD [2]	β	Mean ± SD [2]	β	95% CI [3]	p-value	p_{FDR} [4]	Mean ± SD [2]	β	95% CI [3]	p-value	p_{FDR} [4]	p_{trend}	$p_{trend\ FDR}$ [4]
**SPHINGOLIPIDS**																				
Hydroxysphingomyelin C14:1	SM OH C14:1	4.0 ± 1.0	0.06	(-0.03;0.15)	0.209	0.608	3.6 ± 0.9	ref.	4.2 ± 1.2	0.15	(-0.16;0.46)	0.341	0.643	4.2 ± 0.9	0.23	(-0.09;0.54)	0.158	0.607	0.128	0.598
Hydroxysphingomyelin C16:1	SM OH C16:1	1.9 ± 0.5	0.08	(-0.02;0.17)	0.119	0.469	1.7 ± 0.4	ref.	1.9 ± 0.5	0.25	(-0.07;0.57)	0.122	0.493	1.9 ± 0.5	0.29	(-0.04;0.61)	0.084	0.565	0.070	0.549
Hydroxysphingomyelin C22:1	SM OH C22:1	4.4 ± 1.1	0.05	(-0.05;0.15)	0.302	0.718	4.1 ± 1.0	ref.	4.5 ± 1.2	0.05	(-0.27;0.38)	0.749	0.895	4.6 ± 1.0	0.21	(-0.12;0.54)	0.216	0.674	0.162	0.598
Hydroxysphingomyelin C22:2	SM OH C22:2	3.3 ± 0.9	0.05	(-0.04;0.14)	0.252	0.637	3.0 ± 0.8	ref.	3.5 ± 1.0	0.18	(-0.13;0.49)	0.248	0.564	3.5 ± 0.8	0.28	(-0.03;0.59)	0.078	0.558	0.052	0.447
Hydroxysphingomyelin C24:1	SM OH C24:1	0.3 ± 0.1	0.06	(-0.04;0.17)	0.239	0.629	0.3 ± 0.1	ref.	0.3 ± 0.1	0.25	(-0.10;0.60)	0.161	0.514	0.3 ± 0.1	0.27	(-0.08;0.62)	0.129	0.578	0.091	0.555
Sphingomyelin C16:0	SM C16:0	67.2 ± 13.6	-0.01	(-0.10;0.09)	0.863	0.943	65.3 ± 14.5	ref.	67.9 ± 13.2	-0.09	(-0.41;0.23)	0.590	0.876	68.3 ± 13.1	-0.04	(-0.36;0.29)	0.832	0.935	0.939	0.979
Sphingomyelin C16:1	SM C16:1	9.5 ± 2.2	-0.02	(-0.11;0.07)	0.615	0.852	9.0 ± 2.0	ref.	9.7 ± 2.4	-0.07	(-0.38;0.23)	0.634	0.876	9.7 ± 2.1	-0.03	(-0.34;0.27)	0.824	0.935	0.975	0.985
Sphingomyelin C18:0	SM C18:0	12.0 ± 3.3	0.01	(-0.09;0.11)	0.853	0.943	11.7 ± 4.0	ref.	12.2 ± 3.1	-0.01	(-0.35;0.33)	0.949	0.967	12.2 ± 2.7	0.05	(-0.28;0.39)	0.753	0.910	0.703	0.897
Sphingomyelin C18:1	SM C18:1	5.7 ± 1.6	0.00	(-0.09;0.09)	0.976	0.983	5.4 ± 1.8	ref.	5.8 ± 1.7	0.01	(-0.31;0.33)	0.954	0.967	5.8 ± 1.5	0.06	(-0.26;0.37)	0.731	0.909	0.620	0.823
Sphingomyelin C20:2	SM C20:2	0.2 ± 0.1	0.03	(-0.07;0.12)	0.566	0.825	0.1 ± 0.1	ref.	0.2 ± 0.1	0.16	(-0.16;0.49)	0.324	0.629	0.15 ± 0.05	0.09	(-0.24;0.41)	0.607	0.847	0.535	0.804
Sphingomyelin C24:0	SM C24:0	6.1 ± 1.4	-0.01	(-0.11;0.09)	0.896	0.964	6.0 ± 1.4	ref.	6.1 ± 1.3	-0.13	(-0.47;0.21)	0.456	0.774	6.3 ± 1.4	0.03	(-0.32;0.37)	0.872	0.935	0.726	0.916
Sphingomyelin C24:1	SM C24:1	13.9 ± 3.4	0.00	(-0.10;0.10)	0.960	0.983	13.9 ± 4.4	ref.	13.8 ± 2.8	-0.04	(-0.39;0.30)	0.809	0.903	13.9 ± 2.9	0.02	(-0.33;0.36)	0.913	0.964	0.834	0.916
Sphingomyelin C26:0	SM C26:0	0.04 ± 0.01	0.02	(-0.09;0.12)	0.735	0.910	0.03 ± 0.01	ref.	0.04 ± 0.01	0.33	(-0.03;0.68)	0.074	0.430	0.04 ± 0.02	0.24	(-0.12;0.59)	0.192	0.659	0.131	0.598
Sphingomyelin C26:1	SM C26:1	0.10 ± 0.04	0.05	(-0.05;0.16)	0.338	0.718	0.1 ± 0.1	ref.	0.10 ± 0.03	0.08	(-0.28;0.44)	0.674	0.876	0.11 ± 0.03	0.22	(-0.14;0.58)	0.230	0.682	0.197	0.643
**HEXOSES**																				
Hexoses	H1	5574.0 ± 1481.3	-0.03	(-0.13;0.08)	0.620	0.852	5590.4 ± 1221.1	ref.	5440.8 ± 1494.2	-0.28	(-0.64;0.08)	0.131	0.493	5690.8 ± 1701.5	-0.11	(-0.48;0.25)	0.547	0.815	0.488	0.794

Metabolite	Abbreviation	Mean ± SD [2]	Western pattern																	
			Continuous				Tertile 1		Tertile 2					Tertile 3						
			β	95% CI [3]	*p*-value	p_{FDR} [4]	Mean ± SD [2]	β	Mean ± SD [2]	β	95% CI [3]	*p*-value	p_{FDR} [4]	Mean ± SD [2]	β	95% CI [3]	*p*-value	p_{FDR} [4]	p_{trend}	$p_{trend\ FDR}$ [4]
**ACYLCARNITINES**																				
Carnitine	C0	36.0 ± 7.3	-0.03	(-0.18;0.12)	0.696	0.818	36.8 ± 7.7	ref.	35.4 ± 7.3	-0.18	(-0.54;0.17)	0.313	0.969	35.7 ± 7.0	-0.20	(-0.57;0.17)	0.288	0.543	0.261	0.514
Acetylcarnitine	C2	6.0 ± 1.9	0.02	(-0.13;0.17)	0.823	0.892	6.2 ± 2.0	ref.	6.0 ± 1.7	-0.07	(-0.42;0.27)	0.675	0.969	5.7 ± 2.0	-0.26	(-0.62;0.11)	0.168	0.386	0.202	0.424
Propionylcarnitine	C3	0.5 ± 0.2	-0.03	(-0.18;0.12)	0.687	0.815	0.5 ± 0.2	ref.	0.5 ± 0.2	-0.05	(-0.40;0.30)	0.790	0.969	0.4 ± 0.1	-0.07	(-0.43;0.30)	0.716	0.824	0.707	0.839
Butyrylcarnitine	C4	0.2 ± 0.1	0.07	(-0.08;0.23)	0.342	0.512	0.2 ± 0.1	ref.	0.2 ± 0.1	0.01	(-0.34;0.37)	0.955	0.997	0.2 ± 0.1	0.03	(-0.34;0.40)	0.882	0.944	0.889	0.943
Valerylcarnitine	C5	0.2 ± 0.1	0.06	(-0.08;0.21)	0.379	0.535	0.2 ± 0.1	ref.	0.2 ± 0.1	-0.02	(-0.35;0.31)	0.905	0.997	0.2 ± 0.1	0.16	(-0.19;0.50)	0.371	0.630	0.389	0.652
Decenoylcarnitine	C10:1	0.1 ± 0.1	0.02	(-0.13;0.18)	0.762	0.881	0.1 ± 0.1	ref.	0.1 ± 0.1	-0.25	(-0.60;0.11)	0.173	0.969	0.1 ± 0.1	-0.16	(-0.53;0.21)	0.399	0.644	0.347	0.598
Tetradecenoylcarnitine	C14:1	0.05 ± 0.02	0.12	(-0.03;0.27)	0.115	0.253	0.06 ± 0.03	ref.	0.05 ± 0.02	-0.06	(-0.40;0.29)	0.750	0.969	0.05 ± 0.03	0.04	(-0.33;0.40)	0.846	0.926	0.901	0.943
Tetradecadienylcarnitine	C14:2	0.02 ± 0.01	0.05	(-0.10;0.19)	0.550	0.683	0.03 ± 0.01	ref.	0.02 ± 0.01	-0.13	(-0.48;0.21)	0.454	0.969	0.02 ± 0.01	-0.08	(-0.44;0.28)	0.677	0.804	0.622	0.806
Hexadecanoylcarnitine	C16	0.10 ± 0.03	0.10	(-0.05;0.25)	0.187	0.360	0.11 ± 0.03	ref.	0.10 ± 0.02	-0.13	(-0.47;0.22)	0.469	0.969	0.11 ± 0.03	0.16	(-0.20;0.52)	0.382	0.640	0.489	0.730
Octadecanoylcarnitine	C18	0.05 ± 0.01	0.15	(0.00;0.30)	0.056	0.149	0.05 ± 0.01	ref.	0.05 ± 0.01	0.01	(-0.34;0.36)	0.951	0.997	0.05 ± 0.01	0.25	(-0.11;0.62)	0.175	0.386	0.210	0.428
Octadecenoylcarnitine	C18:1	0.09 ± 0.03	-0.03	(-0.19;0.12)	0.664	0.802	0.09 ± 0.04	ref.	0.08 ± 0.02	-0.20	(-0.56;0.16)	0.279	0.969	0.08 ± 0.03	-0.12	(-0.49;0.26)	0.538	0.731	0.469	0.714
Octadecadienylcarnitine	C18:2	0.03 ± 0.01	-0.15	(-0.30;0.01)	0.060	0.159	0.04 ± 0.02	ref.	0.03 ± 0.01	-0.29	(-0.65;0.07)	0.116	0.969	0.03 ± 0.01	-0.26	(-0.63;0.11)	0.174	0.386	0.143	0.392
**AMINO ACIDS**																				
Alanine	Ala	382.8 ± 100.7	0.01	(-0.14;0.17)	0.886	0.924	385.3 ± 93.4	ref.	380.1 ± 108.3	0.00	(-0.36;0.36)	0.990	0.997	383.0 ± 101.2	-0.01	(-0.38;0.37)	0.962	0.971	0.966	0.973
Arginine	Arg	81.3 ± 23.5	0.11	(-0.04;0.26)	0.154	0.312	78.9 ± 22.3	ref.	85.1 ± 23.1	0.27	(-0.08;0.62)	0.137	0.969	80.0 ± 24.7	0.13	(-0.24;0.50)	0.501	0.712	0.412	0.674
Asparagine	Asn	43.5 ± 9.0	0.02	(-0.13;0.17)	0.788	0.886	42.9 ± 8.3	ref.	44.9 ± 9.4	0.24	(-0.11;0.58)	0.178	0.969	42.6 ± 9.4	0.01	(-0.35;0.37)	0.963	0.971	0.838	0.913
Aspartate	Asp	11.8 ± 4.0	-0.01	(-0.16;0.15)	0.934	0.948	11.8 ± 3.8	ref.	11.8 ± 4.2	-0.02	(-0.38;0.34)	0.922	0.997	11.8 ± 4	0.09	(-0.28;0.47)	0.623	0.792	0.669	0.815
Citrulline	Cit	36.5 ± 10.2	-0.01	(-0.17;0.14)	0.874	0.922	36.4 ± 10.2	ref.	37.0 ± 10.2	0.16	(-0.20;0.51)	0.394	0.969	35.9 ± 10.3	0.10	(-0.28;0.47)	0.610	0.792	0.587	0.795
Glutamine	Gln	696.5 ± 98.5	0.08	(-0.06;0.23)	0.255	0.437	692.5 ± 86.4	ref.	703.0 ± 102.3	0.05	(-0.29;0.38)	0.788	0.969	694.1 ± 106.8	-0.01	(-0.37;0.34)	0.937	0.971	0.987	0.987
Glutamate	Glu	80.0 ± 29.7	0.09	(-0.06;0.24)	0.241	0.430	80.1 ± 31	ref.	77.9 ± 30.0	0.05	(-0.31;0.40)	0.798	0.969	81.9 ± 28.4	0.18	(-0.19;0.54)	0.346	0.603	0.348	0.598
Glycine	Gly	215.6 ± 59.8	-0.12	(-0.26;0.02)	0.096	0.218	221.4 ± 65.2	ref.	220.6 ± 61.6	-0.07	(-0.39;0.26)	0.681	0.969	204.8 ± 51.1	-0.27	(-0.61;0.07)	0.120	0.335	0.159	0.392
Histidine	His	71.1 ± 12.4	0.16	(0.01;0.31)	0.035	0.107	69.5 ± 11.4	ref.	70.9 ± 13.4	0.08	(-0.27;0.43)	0.648	0.969	73.0 ± 12.3	0.19	(-0.17;0.55)	0.300	0.553	0.306	0.561
Isoleucine	Ile	75.4 ± 23.9	-0.08	(-0.22;0.07)	0.296	0.490	75.3 ± 27.3	ref.	79.1 ± 27.3	0.16	(-0.18;0.50)	0.356	0.969	71.7 ± 21.6	-0.15	(-0.51;0.20)	0.392	0.641	0.496	0.730
Leucine	Leu	133.8 ± 42.3	0.02	(-0.13;0.16)	0.839	0.892	131.6 ± 40.3	ref.	139.4 ± 47.2	0.21	(-0.14;0.55)	0.241	0.969	130.4 ± 39	0.00	(-0.36;0.35)	0.979	0.979	0.892	0.943
Lysine	Lys	193.5 ± 41.8	0.05	(-0.09;0.20)	0.471	0.625	191.6 ± 34.0	ref.	199.4 ± 43.1	0.17	(-0.18;0.51)	0.339	0.969	189.5 ± 47.1	-0.09	(-0.45;0.27)	0.629	0.792	0.749	0.858
Methionine	Met	20.20 ± 5.8	-0.02	(-0.17;0.13)	0.787	0.886	20.8 ± 5.8	ref.	20.4 ± 6.0	0.03	(-0.31;0.38)	0.856	0.997	19.5 ± 5.6	-0.12	(-0.48;0.24)	0.498	0.712	0.506	0.730
Ornithine	Orn	71.3 ± 24.6	-0.07	(-0.22;0.08)	0.366	0.521	72.3 ± 22.3	ref.	70.6 ± 22.8	-0.07	(-0.43;0.28)	0.680	0.969	71.1 ± 28.5	-0.05	(-0.42;0.32)	0.777	0.874	0.747	0.858
Phenylalanine	Phe	62.8 ± 13.6	-0.02	(-0.16;0.13)	0.832	0.892	63.8 ± 13.0	ref.	63.8 ± 14.4	0.07	(-0.27;0.41)	0.684	0.969	60.7 ± 13.4	-0.12	(-0.47;0.24)	0.521	0.720	0.555	0.761
Proline	Pro	242.6 ± 79.5	-0.07	(-0.22;0.08)	0.344	0.512	246.5 ± 70.8	ref.	253.1 ± 86.6	0.05	(-0.30;0.39)	0.788	0.969	228.2 ± 79.4	-0.21	(-0.56;0.15)	0.257	0.500	0.334	0.597
Serine	Ser	93 ± 20.3	-0.02	(-0.17;0.14)	0.832	0.892	92.5 ± 19.6	ref.	95.5 ± 17.1	0.24	(-0.11;0.60)	0.176	0.969	91.0 ± 23.7	-0.01	(-0.38;0.36)	0.952	0.971	0.929	0.952
Threonine	Thr	108.7 ± 27.7	-0.08	(-0.22;0.07)	0.320	0.505	109.2 ± 24.0	ref.	110.2 ± 28.3	0.09	(-0.26;0.43)	0.624	0.969	106.8 ± 30.7	-0.12	(-0.49;0.24)	0.503	0.712	0.550	0.761
Tryptophan	Trp	53.1 ± 11.0	0.01	(-0.14;0.16)	0.890	0.924	54.2 ± 12.3	ref.	51.9 ± 11.6	-0.17	(-0.52;0.18)	0.341	0.969	53.2 ± 8.8	-0.07	(-0.44;0.29)	0.699	0.819	0.640	0.806
Tyrosine	Tyr	74.8 ± 24.1	-0.01	(-0.16;0.14)	0.897	0.925	77.2 ± 26.5	ref.	75.7 ± 25.1	0.08	(-0.28;0.43)	0.672	0.969	71.3 ± 20.2	-0.02	(-0.39;0.34)	0.901	0.951	0.930	0.952
Valine	Val	242.1 ± 64.0	0.02	(-0.13;0.16)	0.826	0.892	241.6 ± 62	ref.	247.9 ± 75.2	0.09	(-0.25;0.43)	0.598	0.969	236.7 ± 53.6	-0.07	(-0.43;0.29)	0.703	0.819	0.769	0.873
**BIOGENIC AMINES**																				
Creatinine	Creatinine	74.5 ± 17.4	0.04	(-0.08;0.16)	0.542	0.683	74.8 ± 19.3	ref.	75.1 ± 18.2	0.06	(-0.22;0.35)	0.673	0.969	73.5 ± 14.4	-0.03	(-0.33;0.27)	0.850	0.926	0.928	0.952
Kynurenine	Kynurenine	2.7 ± 0.7	-0.07	(-0.21;0.08)	0.364	0.521	2.9 ± 0.8	ref.	2.5 ± 0.6	-0.38	(-0.71;-0.05)	**0.025**	0.969	2.7 ± 0.8	-0.16	(-0.51;0.18)	0.360	0.619	0.220	0.440
Sarcosine	Sarcosine	4.4 ± 1.1	0.05	(-0.11;0.20)	0.555	0.683	4.4 ± 1.1	ref.	4.3 ± 1.0	-0.08	(-0.43;0.27)	0.662	0.969	4.5 ± 1.2	0.09	(-0.28;0.46)	0.632	0.792	0.638	0.806
Serotonin	Serotonin	0.8 ± 0.6	0.06	(-0.10;0.21)	0.470	0.625	0.7 ± 0.5	ref.	0.7 ± 0.6	-0.02	(-0.37;0.34)	0.927	0.997	0.8 ± 0.5	0.12	(-0.25;0.48)	0.540	0.731	0.625	0.806
Taurine	Taurine	120.9 ± 49.4	0.07	(-0.08;0.23)	0.346	0.512	120.6 ± 47.0	ref.	121.4 ± 51.6	0.00	(-0.36;0.36)	0.985	0.997	120.6 ± 50.3	0.04	(-0.34;0.41)	0.849	0.926	0.868	0.938
Trans 4-Hydroxyproline	t4-OH-Pro	10.2 ± 5.0	0.03	(-0.12;0.18)	0.724	0.843	10.6 ± 6.1	ref.	10.3 ± 4.6	-0.01	(-0.35;0.34)	0.967	0.997	9.6 ± 4.2	-0.12	(-0.48;0.24)	0.505	0.712	0.557	0.761
Asymmetric dimethylarginine	ADMA	0.5 ± 0.1	-0.14	(-0.27;0.00)	**0.046**	0.130	0.5 ± 0.1	ref.	0.5 ± 0.1	0.00	(-0.32;0.31)	0.995	0.997	0.4 ± 0.1	-0.31	(-0.64;0.02)	0.068	0.238	0.104	0.316
Symmetric dimethylarginine	SDMA	0.5 ± 0.1	0.06	(-0.08;0.20)	0.417	0.570	0.5 ± 0.1	ref.	0.5 ± 0.1	0.11	(-0.22;0.43)	0.525	0.969	0.4 ± 0.1	-0.12	(-0.47;0.22)	0.476	0.708	0.601	0.797
**GLYCEROPHOSPHOLIPIDS**																				
Lysophosphatidylcholine (acyl) C16:0	lysoPC a C16:0	90.4 ± 20.1	0.05	(-0.11;0.20)	0.553	0.683	88.6 ± 19.6	ref.	89.8 ± 20.5	-0.05	(-0.41;0.32)	0.802	0.969	92.8 ± 20.2	0.10	(-0.28;0.48)	0.605	0.792	0.596	0.797
Lysophosphatidylcholine (acyl) C16:1	lysoPC a C16:1	2.6 ± 0.9	0.09	(-0.07;0.24)	0.271	0.459	2.5 ± 0.9	ref.	2.6 ± 1.1	0.01	(-0.36;0.37)	0.976	0.997	2.6 ± 0.8	0.15	(-0.22;0.53)	0.432	0.673	0.430	0.680
Lysophosphatidylcholine (acyl) C17:0	lysoPC a C17:0	1.6 ± 0.5	0.07	(-0.08;0.22)	0.356	0.519	1.5 ± 0.4	ref.	1.6 ± 0.5	0.10	(-0.25;0.44)	0.573	0.969	1.6 ± 0.6	0.22	(-0.14;0.58)	0.225	0.469	0.200	0.424
Lysophosphatidylcholine (acyl) C18:0	lysoPC a C18:0	26.4 ± 6.8	0.05	(-0.11;0.20)	0.550	0.683	25.5 ± 5.7	ref.	26.2 ± 6.6	-0.03	(-0.38;0.32)	0.858	0.997	27.4 ± 7.9	0.15	(-0.22;0.52)	0.426	0.671	0.422	0.680
Lysophosphatidylcholine (acyl) C18:1	lysoPC a C18:1	17.9 ± 5.4	0.11	(-0.04;0.26)	0.139	0.292	16.9 ± 5.0	ref.	18.4 ± 5.7	0.16	(-0.20;0.51)	0.386	0.969	18.5 ± 5.3	0.23	(-0.13;0.60)	0.216	0.459	0.184	0.399
Lysophosphatidylcholine (acyl) C18:2	lysoPC a C18:2	29.2 ± 10.4	0.05	(-0.10;0.20)	0.515	0.663	27.2 ± 9.6	ref.	30.4 ± 11	0.10	(-0.24;0.45)	0.558	0.969	30.20 ± 10.3	0.11	(-0.25;0.47)	0.551	0.739	0.468	0.714
Lysophosphatidylcholine (acyl) C20:3	lysoPC a C20:3	2.0 ± 0.7	0.04	(-0.11;0.19)	0.591	0.720	1.9 ± 0.7	ref.	2.1 ± 0.7	0.06	(-0.29;0.41)	0.756	0.969	2.2 ± 0.7	0.14	(-0.22;0.51)	0.444	0.684	0.412	0.674
Lysophosphatidylcholine (acyl) C20:4	lysoPC a C20:4	5.9 ± 1.7	0.00	(-0.15;0.16)	0.961	0.962	6.0 ± 2.0	ref.	5.8 ± 1.5	-0.15	(-0.51;0.20)	0.401	0.969	5.9 ± 1.6	-0.08	(-0.45;0.29)	0.663	0.804	0.667	0.815
Lysophosphatidylcholine (acyl) C24:0	lysoPC a C24:0	0.14 ± 0.03	-0.05	(-0.19;0.09)	0.499	0.656	0.14 ± 0.03	ref.	0.13 ± 0.03	-0.20	(-0.52;0.12)	0.220	0.969	0.14 ± 0.03	-0.03	(-0.36;0.31)	0.877	0.944	0.802	0.896
Lysophosphatidylcholine (acyl) C28:1	lysoPC a C28:1	0.3 ± 0.1	0.08	(-0.04;0.21)	0.190	0.360	0.3 ± 0.1	ref.	0.3 ± 0.1	-0.05	(-0.34;0.24)	0.735	0.969	0.3 ± 0.1	0.18	(-0.12;0.48)	0.245	0.489	0.283	0.534
Phosphatidylcholine (diacyl) C28:1	PC aa C28:1	3.0 ± 0.8	0.31	(0.18;0.44)	**0.000**	**0.001**	2.9 ± 0.8	ref.	3.0 ± 0.8	0.18	(-0.14;0.50)	0.274	0.969	3.1 ± 0.7	0.59	(0.26;0.92)	**0.001**	0.083	**0.001**	0.100
Phosphatidylcholine (diacyl) C30:0	PC aa C30:0	4.0 ± 1.3	0.23	(0.09;0.38)	**0.002**	**0.016**	3.8 ± 1.5	ref.	4.0 ± 1.3	0.22	(-0.13;0.57)	0.210	0.969	4.2 ± 1.2	0.47	(0.11;0.84)	**0.012**	0.095	**0.013**	0.108
Phosphatidylcholine (diacyl) C32:0	PC aa C32:0	26.6 ± 5.5	0.17	(0.02;0.31)	**0.025**	0.081	26.5 ± 5.6	ref.	26.8 ± 5.6	0.11	(-0.24;0.45)	0.545	0.969	26.6 ± 5.3	0.21	(-0.15;0.56)	0.257	0.500	0.266	0.516

			Continuous				Tertile 1		Tertile 2					Tertile 3						
**Metabolite**	**Abbreviation**	Mean ± SD [2]	β	95% CI [3]	*p* -value	p_{FDR} [4]	Mean ± SD [2]	β	Mean ± SD [2]	β	95% CI [3]	*p* -value	p_{FDR} [4]	Mean ± SD [2]	β	95% CI [3]	*p* -value	p_{FDR} [4]	p_{trend}	$p_{trend\ FDR}$ [4]
Phosphatidylcholine (diacyl) C32:1	PC aa C32:1	32.0 ± 16.2	0.17	(0.02;0.33)	**0.027**	0.085	31.2 ± 17.3	ref.	31.4 ± 16	0.11	(-0.25;0.47)	0.543	0.969	33.5 ± 15.2	0.30	(-0.07;0.67)	0.114	0.333	0.123	0.351
Phosphatidylcholine (diacyl) C32:2	PC aa C32:2	8.3 ± 3.1	0.21	(0.06;0.36)	**0.005**	**0.031**	7.9 ± 3.5	ref.	8.3 ± 3.0	0.13	(-0.22;0.48)	0.467	0.969	8.9 ± 2.7	0.40	(0.04;0.77)	**0.030**	0.150	**0.031**	0.154
Phosphatidylcholine (diacyl) C32:3	PC aa C32:3	0.9 ± 0.2	0.12	(-0.02;0.26)	0.086	0.210	0.9 ± 0.3	ref.	0.9 ± 0.2	0.10	(-0.22;0.43)	0.528	0.969	0.9 ± 0.2	0.27	(-0.07;0.60)	0.121	0.335	0.117	0.340
Phosphatidylcholine (diacyl) C34:1	PC aa C34:1	365.4 ± 82.8	0.24	(0.09;0.38)	**0.002**	**0.016**	352.8 ± 83.8	ref.	368.1 ± 82.1	0.22	(-0.13;0.57)	0.218	0.969	375.3 ± 82.3	0.40	(0.04;0.77)	**0.033**	0.153	**0.032**	0.154
Phosphatidylcholine (diacyl) C34:2	PC aa C34:2	739.8 ± 128.1	0.20	(0.05;0.35)	**0.009**	**0.036**	718.4 ± 135.0	ref.	747.2 ± 123.7	0.19	(-0.17;0.54)	0.307	0.969	753.7 ± 124.4	0.32	(-0.06;0.69)	0.097	0.304	0.086	0.295
Phosphatidylcholine (diacyl) C34:3	PC aa C34:3	30.1 ± 10.2	0.15	(0.00;0.29)	0.052	0.143	29.1 ± 10.7	ref.	30.6 ± 10.8	0.12	(-0.23;0.46)	0.513	0.969	30.8 ± 8.9	0.27	(-0.09;0.63)	0.150	0.374	0.140	0.391
Phosphatidylcholine (diacyl) C34:4	PC aa C34:4	3.1 ± 1.2	0.19	(0.04;0.33)	**0.014**	0.050	3.0 ± 1.4	ref.	3.1 ± 1.1	0.10	(-0.25;0.44)	0.577	0.969	3.3 ± 1.0	0.36	(0.00;0.72)	0.050	0.197	0.051	0.209
Phosphatidylcholine (diacyl) C36:0	PC aa C36:0	3.2 ± 1.0	0.10	(-0.04;0.25)	0.168	0.335	3.1 ± 0.9	ref.	3.3 ± 1.1	0.10	(-0.24;0.44)	0.572	0.969	3.2 ± 1.1	0.24	(-0.11;0.60)	0.179	0.386	0.173	0.392
Phosphatidylcholine (diacyl) C36:1	PC aa C36:1	62.9 ± 17.8	0.24	(0.1;0.38)	**0.001**	**0.010**	58.7 ± 17.1	ref.	63.3 ± 17.6	0.24	(-0.08;0.57)	0.146	0.969	66.9 ± 17.9	0.54	(0.2;0.89)	**0.002**	0.089	**0.002**	0.100
Phosphatidylcholine (diacyl) C36:2	PC aa C36:2	384.7 ± 79.7	0.19	(0.05;0.33)	**0.009**	**0.036**	366.3 ± 73.4	ref.	390.7 ± 80.5	0.22	(-0.12;0.55)	0.202	0.969	397.3 ± 82.7	0.38	(0.03;0.73)	**0.033**	0.153	**0.028**	0.149
Phosphatidylcholine (diacyl) C36:3	PC aa C36:3	192.6 ± 43.8	0.21	(0.07;0.35)	**0.004**	**0.024**	183 ± 44.8	ref.	193.0 ± 41.1	0.18	(-0.15;0.51)	0.289	0.969	201.9 ± 44	0.41	(0.06;0.76)	**0.021**	0.119	**0.021**	0.126
Phosphatidylcholine (diacyl) C36:4	PC aa C36:4	291.5 ± 72.0	0.17	(0.02;0.31)	**0.023**	0.078	297.2 ± 89.5	ref.	285.7 ± 61	-0.08	(-0.42;0.26)	0.635	0.969	291.5 ± 62.6	0.12	(-0.23;0.48)	0.493	0.712	0.529	0.754
Phosphatidylcholine (diacyl) C36:5	PC aa C36:5	45.8 ± 27.8	0.00	(-0.15;0.16)	0.962	0.962	44.4 ± 21.2	ref.	47.1 ± 34.4	-0.01	(-0.36;0.34)	0.954	0.997	45.7 ± 26.5	0.08	(-0.28;0.45)	0.657	0.804	0.682	0.824
Phosphatidylcholine (diacyl) C36:6	PC aa C36:6	1.5 ± 0.6	0.10	(-0.05;0.25)	0.196	0.360	1.5 ± 0.6	ref.	1.6 ± 0.7	0.14	(-0.20;0.48)	0.426	0.969	1.6 ± 0.6	0.30	(-0.06;0.66)	0.103	0.315	0.093	0.310
Phosphatidylcholine (diacyl) C38:0	PC aa C38:0	2.9 ± 0.9	0.08	(-0.07;0.22)	0.288	0.482	2.8 ± 0.9	ref.	2.9 ± 0.9	0.05	(-0.28;0.39)	0.759	0.969	2.9 ± 0.9	0.26	(-0.09;0.61)	0.141	0.371	0.162	0.392
Phosphatidylcholine (diacyl) C38:3	PC aa C38:3	53.2 ± 13.6	0.12	(-0.02;0.25)	0.097	0.218	51.5 ± 13.8	ref.	52.2 ± 12.4	0.06	(-0.26;0.38)	0.719	0.969	55.9 ± 14.4	0.34	(0.01;0.68)	**0.042**	0.183	0.051	0.209
Phosphatidylcholine (diacyl) C38:4	PC aa C38:4	131.5 ± 35.0	0.10	(-0.03;0.24)	0.140	0.292	136.2 ± 42.3	ref.	128.0 ± 29.9	-0.14	(-0.46;0.18)	0.385	0.969	130.4 ± 31.5	0.06	(-0.27;0.39)	0.733	0.832	0.807	0.896
Phosphatidylcholine (diacyl) C38:5	PC aa C38:5	66.2 ± 19.0	0.07	(-0.07;0.21)	0.328	0.511	66.7 ± 19.1	ref.	66.0 ± 20.8	-0.08	(-0.41;0.25)	0.645	0.969	66.0 ± 17.3	0.07	(-0.27;0.42)	0.678	0.804	0.726	0.846
Phosphatidylcholine (diacyl) C38:6	PC aa C38:6	101.1 ± 33.6	0.02	(-0.13;0.17)	0.793	0.886	102.1 ± 29.9	ref.	101.1 ± 37.8	-0.08	(-0.42;0.27)	0.663	0.969	100.2 ± 33.1	0.10	(-0.26;0.45)	0.603	0.792	0.630	0.806
Phosphatidylcholine (diacyl) C40:2	PC aa C40:2	0.3 ± 0.1	0.09	(-0.06;0.23)	0.244	0.430	0.3 ± 0.1	ref.	0.3 ± 0.1	0.13	(-0.21;0.46)	0.458	0.969	0.3 ± 0.1	0.24	(-0.11;0.59)	0.177	0.386	0.176	0.392
Phosphatidylcholine (diacyl) C40:3	PC aa C40:3	0.4 ± 0.1	0.07	(-0.07;0.21)	0.332	0.511	0.4 ± 0.1	ref.	0.4 ± 0.2	0.17	(-0.16;0.50)	0.326	0.969	0.4 ± 0.1	0.21	(-0.13;0.56)	0.228	0.469	0.211	0.428
Phosphatidylcholine (diacyl) C40:4	PC aa C40:4	3.0 ± 0.8	0.19	(0.05;0.34)	**0.011**	**0.041**	2.9 ± 0.8	ref.	2.9 ± 0.8	-0.02	(-0.36;0.33)	0.924	0.997	3.2 ± 0.9	0.28	(-0.08;0.65)	0.124	0.335	0.153	0.392
Phosphatidylcholine (diacyl) C40:5	PC aa C40:5	8.3 ± 2.4	0.10	(-0.05;0.25)	0.191	0.360	8.3 ± 2.4	ref.	8.0 ± 2.4	-0.11	(-0.45;0.23)	0.529	0.969	8.5 ± 2.6	0.13	(-0.22;0.49)	0.463	0.701	0.546	0.761
Phosphatidylcholine (diacyl) C40:6	PC aa C40:6	28.3 ± 10.2	-0.02	(-0.17;0.12)	0.775	0.886	29.2 ± 9.8	ref.	27.8 ± 10.5	-0.09	(-0.43;0.24)	0.590	0.969	27.9 ± 10.4	0.08	(-0.27;0.43)	0.659	0.804	0.717	0.843
Phosphatidylcholine (diacyl) C42:0	PC aa C42:0	0.3 ± 0.1	-0.05	(-0.19;0.09)	0.512	0.663	0.4 ± 0.1	ref.	0.3 ± 0.1	-0.09	(-0.42;0.24)	0.595	0.969	0.3 ± 0.1	-0.01	(-0.36;0.33)	0.935	0.971	0.899	0.943
Phosphatidylcholine (diacyl) C42:1	PC aa C42:1	0.18 ± 0.05	0.01	(-0.13;0.15)	0.920	0.941	0.2 ± 0.1	ref.	0.18 ± 0.05	-0.02	(-0.35;0.31)	0.909	0.997	0.18 ± 0.05	0.09	(-0.26;0.43)	0.625	0.792	0.666	0.815
Phosphatidylcholine (diacyl) C42:2	PC aa C42:2	0.16 ± 0.04	0.11	(-0.03;0.26)	0.134	0.290	0.15 ± 0.04	ref.	0.16 ± 0.05	0.07	(-0.27;0.41)	0.670	0.969	0.16 ± 0.04	0.18	(-0.17;0.54)	0.310	0.553	0.315	0.571
Phosphatidylcholine (diacyl) C42:4	PC aa C42:4	0.10 ± 0.02	0.10	(-0.05;0.25)	0.196	0.360	0.10 ± 0.02	ref.	0.10 ± 0.02	0.02	(-0.34;0.38)	0.910	0.997	0.10 ± 0.03	0.04	(-0.33;0.41)	0.840	0.926	0.834	0.913
Phosphatidylcholine (diacyl) C42:5	PC aa C42:5	0.3 ± 0.1	0.09	(-0.06;0.24)	0.249	0.434	0.2 ± 0.1	ref.	0.3 ± 0.1	0.07	(-0.29;0.42)	0.715	0.969	0.3 ± 0.1	0.21	(-0.16;0.58)	0.268	0.513	0.296	0.550
Phosphatidylcholine (diacyl) C42:6	PC aa C42:6	0.4 ± 0.1	0.09	(-0.06;0.24)	0.240	0.430	0.4 ± 0.1	ref.	0.4 ± 0.1	0.00	(-0.35;0.35)	0.988	0.997	0.4 ± 0.2	0.15	(-0.21;0.52)	0.404	0.644	0.449	0.699
Phosphatidylcholine (acyl-alkyl) C30:0	PC ae C30:0	0.3 ± 0.1	0.20	(0.06;0.34)	**0.006**	**0.033**	0.3 ± 0.1	ref.	0.3 ± 0.1	0.27	(-0.07;0.60)	0.119	0.969	0.4 ± 0.1	0.49	(0.15;0.84)	**0.006**	0.089	**0.005**	0.100
Phosphatidylcholine (acyl-alkyl) C30:2	PC ae C30:2	0.08 ± 0.02	0.14	(0.01;0.26)	**0.037**	0.108	0.08 ± 0.02	ref.	0.08 ± 0.02	0.00	(-0.30;0.29)	0.997	0.997	0.09 ± 0.02	0.29	(-0.02;0.59)	0.069	0.238	0.084	0.295
Phosphatidylcholine (acyl-alkyl) C32:1	PC ae C32:1	5.3 ± 1.2	0.22	(0.08;0.36)	**0.002**	**0.016**	5.1 ± 1.2	ref.	5.3 ± 1.3	0.16	(-0.18;0.49)	0.356	0.969	5.3 ± 1.2	0.36	(0.01;0.71)	**0.047**	0.192	**0.049**	0.209
Phosphatidylcholine (acyl-alkyl) C32:2	PC ae C32:2	1.4 ± 0.4	0.19	(0.06;0.33)	**0.006**	**0.034**	1.3 ± 0.3	ref.	1.4 ± 0.4	0.18	(-0.14;0.50)	0.272	0.969	1.4 ± 0.4	0.33	(-0.01;0.67)	0.057	0.214	0.054	0.209
Phosphatidylcholine (acyl-alkyl) C34:0	PC ae C34:0	2.5 ± 0.6	0.24	(0.1;0.39)	**0.001**	**0.010**	2.4 ± 0.7	ref.	2.5 ± 0.7	0.24	(-0.09;0.58)	0.153	0.969	2.5 ± 0.5	0.50	(0.15;0.85)	**0.006**	0.089	**0.006**	0.100
Phosphatidylcholine (acyl-alkyl) C34:1	PC ae C34:1	18.0 ± 3.9	0.23	(0.1;0.37)	**0.001**	**0.010**	17.5 ± 4	ref.	18.3 ± 4.0	0.25	(-0.07;0.57)	0.126	0.969	18.4 ± 3.7	0.44	(0.11;0.78)	**0.010**	0.094	**0.010**	0.108
Phosphatidylcholine (acyl-alkyl) C34:2	PC ae C34:2	20.2 ± 5.3	0.26	(0.12;0.40)	**0.000**	**0.010**	19.0 ± 5.0	ref.	20.9 ± 5.6	0.25	(-0.08;0.58)	0.140	0.969	20.8 ± 5.2	0.44	(0.09;0.78)	**0.013**	0.100	**0.012**	0.108
Phosphatidylcholine (acyl-alkyl) C34:3	PC ae C34:3	13.1 ± 4.0	0.23	(0.09;0.37)	**0.002**	**0.012**	12.2 ± 3.5	ref.	13.6 ± 4.1	0.21	(-0.12;0.54)	0.210	0.969	13.6 ± 4.1	0.42	(0.07;0.76)	**0.020**	0.119	**0.018**	0.121
Phosphatidylcholine (acyl-alkyl) C36:0	PC ae C36:0	1.2 ± 0.3	0.25	(0.1;0.40)	**0.001**	**0.010**	1.1 ± 0.3	ref.	1.2 ± 0.4	0.26	(-0.09;0.61)	0.142	0.969	1.2 ± 0.3	0.48	(0.12;0.84)	**0.010**	0.094	**0.012**	0.108
Phosphatidylcholine (acyl-alkyl) C36:1	PC ae C36:1	11.5 ± 2.7	0.25	(0.12;0.39)	**0.0004**	**0.010**	11 ± 2.7	ref.	11.6 ± 2.8	0.25	(-0.07;0.57)	0.123	0.969	11.8 ± 2.6	0.51	(0.18;0.85)	**0.003**	0.089	**0.003**	0.100
Phosphatidylcholine (acyl-alkyl) C36:2	PC ae C36:2	21.7 ± 5.4	0.20	(0.06;0.33)	**0.005**	**0.029**	20.5 ± 4.9	ref.	22.4 ± 5.4	0.27	(-0.04;0.59)	0.091	0.969	22.2 ± 5.7	0.41	(0.08;0.74)	**0.015**	0.104	**0.011**	0.108
Phosphatidylcholine (acyl-alkyl) C36:3	PC ae C36:3	11.4 ± 2.8	0.23	(0.1;0.37)	**0.001**	**0.010**	10.7 ± 2.7	ref.	11.7 ± 2.9	0.27	(-0.05;0.60)	0.105	0.969	11.7 ± 2.8	0.45	(0.11;0.79)	**0.010**	0.094	**0.009**	0.105
Phosphatidylcholine (acyl-alkyl) C36:4	PC ae C36:4	26.3 ± 6.4	0.26	(0.11;0.40)	**0.001**	**0.010**	25.3 ± 6.3	ref.	26.4 ± 6.3	0.14	(-0.21;0.48)	0.443	0.969	27.1 ± 6.5	0.40	(0.04;0.76)	**0.029**	0.150	**0.032**	0.154
Phosphatidylcholine (acyl-alkyl) C36:5	PC ae C36:5	18.3 ± 5.0	0.19	(0.05;0.34)	**0.009**	**0.036**	17.8 ± 4.5	ref.	18.6 ± 5.1	0.07	(-0.27;0.41)	0.680	0.969	18.5 ± 5.3	0.26	(-0.09;0.61)	0.146	0.374	0.155	0.392
Phosphatidylcholine (acyl-alkyl) C38:0	PC ae C38:0	2.5 ± 0.8	0.06	(-0.08;0.21)	0.402	0.562	2.4 ± 0.8	ref.	2.6 ± 0.9	0.07	(-0.27;0.40)	0.706	0.969	2.5 ± 0.7	0.19	(-0.17;0.54)	0.302	0.553	0.282	0.534
Phosphatidylcholine (acyl-alkyl) C38:2	PC ae C38:2	2.2 ± 0.6	0.19	(0.05;0.33)	**0.007**	**0.036**	2.1 ± 0.6	ref.	2.3 ± 0.6	0.32	(-0.01;0.65)	0.057	0.969	2.3 ± 0.5	0.47	(0.13;0.81)	**0.008**	0.094	**0.006**	0.100
Phosphatidylcholine (acyl-alkyl) C38:3	PC ae C38:3	4.8 ± 1.1	0.19	(0.05;0.32)	**0.007**	**0.036**	4.6 ± 1.1	ref.	4.8 ± 1.2	0.23	(-0.08;0.54)	0.149	0.969	4.9 ± 1.0	0.49	(0.16;0.81)	**0.004**	0.089	**0.004**	0.100
Phosphatidylcholine (acyl-alkyl) C38:4	PC ae C38:4	17.2 ± 3.6	0.25	(0.11;0.39)	**0.001**	**0.010**	17.0 ± 4.1	ref.	17.1 ± 3.5	0.06	(-0.26;0.39)	0.702	0.969	17.6 ± 3.3	0.45	(0.11;0.79)	**0.011**	0.094	**0.015**	0.112
Phosphatidylcholine (acyl-alkyl) C38:5	PC ae C38:5	21.9 ± 4.7	0.19	(0.05;0.33)	**0.009**	**0.036**	21.5 ± 4.8	ref.	22.2 ± 4.6	0.10	(-0.23;0.44)	0.550	0.969	22.1 ± 4.6	0.30	(-0.05;0.65)	0.090	0.302	0.099	0.316
Phosphatidylcholine (acyl-alkyl) C38:6	PC ae C38:6	9.2 ± 2.6	0.13	(-0.01;0.27)	0.068	0.174	9.0 ± 2.3	ref.	9.4 ± 2.8	0.08	(-0.25;0.41)	0.639	0.969	9.4 ± 2.8	0.30	(-0.05;0.64)	0.093	0.303	0.104	0.316
Phosphatidylcholine (acyl-alkyl) C40:1	PC ae C40:1	1.1 ± 0.3	0.08	(-0.07;0.22)	0.316	0.505	1.1 ± 0.3	ref.	1.1 ± 0.3	-0.09	(-0.43;0.25)	0.606	0.969	1.1 ± 0.3	0.13	(-0.22;0.49)	0.466	0.701	0.503	0.730
Phosphatidylcholine (acyl-alkyl) C40:2	PC ae C40:2	1.6 ± 0.4	0.23	(0.1;0.37)	**0.001**	**0.010**	1.5 ± 0.4	ref.	1.5 ± 0.4	0.06	(-0.27;0.38)	0.736	0.969	1.6 ± 0.4	0.50	(0.16;0.83)	**0.004**	0.089	**0.007**	0.100
Phosphatidylcholine (acyl-alkyl) C40:3	PC ae C40:3	0.8 ± 0.2	0.17	(0.04;0.30)	**0.013**	**0.047**	0.8 ± 0.2	ref.	0.8 ± 0.2	0.11	(-0.20;0.42)	0.475	0.969	0.9 ± 0.2	0.42	(0.1;0.74)	**0.011**	0.094	**0.014**	0.108
Phosphatidylcholine (acyl-alkyl) C40:4	PC ae C40:4	2.2 ± 0.4	0.23	(0.09;0.37)	**0.001**	**0.010**	2.2 ± 0.5	ref.	2.2 ± 0.5	0.07	(-0.25;0.40)	0.664	0.969	2.3 ± 0.4	0.42	(0.08;0.76)	**0.017**	0.108	**0.024**	0.137
Phosphatidylcholine (acyl-alkyl) C40:5	PC ae C40:5	3.4 ± 0.7	0.19	(0.05;0.33)	**0.009**	**0.036**	3.3 ± 0.7	ref.	3.3 ± 0.7	-0.02	(-0.34;0.30)	0.896	0.997	3.5 ± 0.6	0.39	(0.05;0.72)	**0.025**	0.136	**0.040**	0.179
Phosphatidylcholine (acyl-alkyl) C40:6	PC ae C40:6	4.8 ± 1.2	0.07	(-0.07;0.21)	0.311	0.505	4.8 ± 1.2	ref.	4.8 ± 1.2	-0.02	(-0.34;0.31)	0.927	0.997	4.8 ± 1.3	0.25	(-0.09;0.59)	0.154	0.374	0.180	0.395
Phosphatidylcholine (acyl-alkyl) C42:1	PC ae C42:1	0.3 ± 0.1	0.07	(-0.07;0.21)	0.348	0.512	0.3 ± 0.1	ref.	0.3 ± 0.1	-0.01	(-0.34;0.32)	0.956	0.997	0.3 ± 0.1	0.06	(-0.28;0.41)	0.719	0.824	0.699	0.836
Phosphatidylcholine (acyl-alkyl) C42:2	PC ae C42:2	0.4 ± 0.1	0.12	(-0.02;0.26)	0.092	0.217	0.4 ± 0.1	ref.	0.4 ± 0.1	0.07	(-0.25;0.39)	0.650	0.969	0.4 ± 0.1	0.34	(0.01;0.67)	**0.047**	0.192	0.055	0.209
Phosphatidylcholine (acyl-alkyl) C42:3	PC ae C42:3	0.6 ± 0.1	0.13	(-0.01;0.27)	0.0782	0.194	0.5 ± 0.2	ref.	0.6 ± 0.1	0.14	(-0.19;0.47)	0.3992	0.969	0.6 ± 0.1	0.33	(-0.01;0.68)	0.0576	0.214	0.059	0.219
Phosphatidylcholine (acyl-alkyl) C42:4	PC ae C42:4	0.7 ± 0.2	0.17	(0.02;0.31)	**0.023**	0.078	0.7 ± 0.2	ref.	0.7 ± 0.2	0.14	(-0.19;0.47)	0.417	0.969	0.7 ± 0.1	0.29	(-0.05;0.64)	0.097	0.304	0.097	0.316
Phosphatidylcholine (acyl-alkyl) C42:5	PC ae C42:5	1.5 ± 0.3	0.13	(-0.01;0.28)	0.074	0.187	1.5 ± 0.4	ref.	1.5 ± 0.3	-0.06	(-0.39;0.27)	0.727	0.969	1.6 ± 0.3	0.27	(-0.07;0.62)	0.125	0.335	0.161	0.392
Phosphatidylcholine (acyl-alkyl) C44:3	PC ae C44:3	0.07 ± 0.02	0.03	(-0.11;0.17)	0.684	0.815	0.07 ± 0.02	ref.	0.07 ± 0.02	0.05	(-0.27;0.38)	0.748	0.969	0.07 ± 0.02	-0.01	(-0.35;0.33)	0.951	0.971	0.953	0.967
Phosphatidylcholine (acyl-alkyl) C44:4	PC ae C44:4	0.2 ± 0.1	0.10	(-0.04;0.24)	0.179	0.352	0.2 ± 0.1	ref.	0.2 ± 0.1	-0.15	(-0.48;0.19)	0.390	0.969	0.23 ± 0.05	0.17	(-0.18;0.52)	0.342	0.603	0.431	0.680
Phosphatidylcholine (acyl-alkyl) C44:5	PC ae C44:5	1.0 ± 0.3	0.13	(-0.02;0.28)	0.092	0.217	1.0 ± 0.3	ref.	1.0 ± 0.3	-0.06	(-0.41;0.28)	0.725	0.969	1.0 ± 0.2	0.19	(-0.17;0.55)	0.307	0.553	0.364	0.618
Phosphatidylcholine (acyl-alkyl) C44:6	PC ae C44:6	0.8 ± 0.2	-0.01	(-0.16;0.13)	0.837	0.892	0.8 ± 0.2	ref.	0.8 ± 0.2	-0.20	(-0.53;0.13)	0.242	0.969	0.8 ± 0.2	-0.02	(-0.37;0.32)	0.887	0.944	0.809	0.896

Metabolite	Abbreviation	Mean ± SD [2]	Continuous				Tertile 1		Tertile 2					Tertile 3						
			β	95% CI [3]	*p*-value	p_{FDR} [4]	Mean ± SD [2]	β	Mean ± SD [2]	β	95% CI [3]	*p*-value	p_{FDR} [4]	Mean ± SD [2]	β	95% CI [3]	*p*-value	p_{FDR} [4]	p_{trend}	$p_{trend\ FDR}$ [4]
**SPHINGOLIPIDS**																				
Hydroxysphingomyelin C14:1	SM OH C14:1	4.0 ± 1.0	0.22	(0.09;0.34)	**0.001**	**0.010**	3.9 ± 1.0	ref.	4.0 ± 1.1	0.11	(-0.18;0.41)	0.462	0.969	4.1 ± 1.0	0.44	(0.13;0.75)	**0.006**	0.089	**0.007**	0.100
Hydroxysphingomyelin C16:1	SM OH C16:1	1.9 ± 0.5	0.18	(0.05;0.31)	**0.009**	**0.036**	1.8 ± 0.5	ref.	1.9 ± 0.5	0.10	(-0.21;0.41)	0.542	0.969	1.9 ± 0.5	0.38	(0.06;0.71)	**0.021**	0.119	**0.026**	0.144
Hydroxysphingomyelin C22:1	SM OH C22:1	4.4 ± 1.1	0.22	(0.09;0.36)	**0.001**	**0.010**	4.2 ± 0.9	ref.	4.3 ± 1.2	0.02	(-0.29;0.33)	0.901	0.997	4.7 ± 1.1	0.43	(0.11;0.76)	**0.010**	0.094	**0.016**	0.112
Hydroxysphingomyelin C22:2	SM OH C22:2	3.3 ± 0.9	0.15	(0.03;0.28)	**0.019**	0.067	3.2 ± 0.8	ref.	3.3 ± 1.0	0.05	(-0.25;0.35)	0.728	0.969	3.4 ± 0.9	0.30	(-0.02;0.61)	0.064	0.233	0.071	0.257
Hydroxysphingomyelin C24:1	SM OH C24:1	0.3 ± 0.1	0.28	(0.14;0.42)	**0.0001**	**0.010**	0.3 ± 0.1	ref.	0.3 ± 0.1	0.06	(-0.27;0.39)	0.715	0.969	0.3 ± 0.1	0.52	(0.18;0.87)	**0.003**	0.089	**0.005**	0.100
Sphingomyelin C16:0	SM C16:0	67.2 ± 13.6	0.17	(0.04;0.31)	**0.011**	**0.041**	65.4 ± 12.3	ref.	67.5 ± 13.3	0.09	(-0.23;0.40)	0.591	0.969	68.6 ± 15.1	0.35	(0.03;0.68)	**0.035**	0.156	**0.038**	0.174
Sphingomyelin C16:1	SM C16:1	9.5 ± 2.2	0.11	(-0.02;0.23)	0.098	0.218	9.3 ± 2.0	ref.	9.6 ± 2.4	0.05	(-0.25;0.35)	0.741	0.969	9.5 ± 2.2	0.21	(-0.09;0.52)	0.176	0.386	0.167	0.392
Sphingomyelin C18:0	SM C18:0	12.0 ± 3.3	0.14	(0.00;0.28)	**0.044**	0.125	11.9 ± 3.1	ref.	12 ± 3.2	0.04	(-0.28;0.37)	0.793	0.969	12.2 ± 3.7	0.25	(-0.09;0.59)	0.153	0.374	0.170	0.392
Sphingomyelin C18:1	SM C18:1	5.7 ± 1.6	0.05	(-0.08;0.18)	0.443	0.599	5.7 ± 1.6	ref.	5.8 ± 1.7	0.03	(-0.28;0.34)	0.851	0.997	5.6 ± 1.6	0.07	(-0.25;0.39)	0.677	0.804	0.643	0.806
Sphingomyelin C20:2	SM C20:2	0.2 ± 0.1	-0.07	(-0.20;0.07)	0.320	0.505	0.16 ± 0.05	ref.	0.1 ± 0.1	-0.41	(-0.72;-0.10)	**0.011**	0.969	0.1 ± 0.1	-0.20	(-0.52;0.13)	0.231	0.469	0.166	0.392
Sphingomyelin C24:0	SM C24:0	6.1 ± 1.4	0.23	(0.09;0.37)	**0.001**	**0.010**	5.7 ± 1.3	ref.	6.0 ± 1.5	0.05	(-0.28;0.38)	0.778	0.969	6.6 ± 1.4	0.43	(0.08;0.77)	**0.015**	0.104	**0.020**	0.126
Sphingomyelin C24:1	SM C24:1	13.9 ± 3.4	0.16	(0.02;0.30)	**0.031**	0.097	13.3 ± 2.8	ref.	14.0 ± 3.8	0.09	(-0.25;0.42)	0.611	0.969	14.2 ± 3.6	0.24	(-0.10;0.59)	0.169	0.386	0.162	0.392
Sphingomyelin C26:0	SM C26:0	0.04 ± 0.01	0.16	(0.01;0.31)	**0.036**	0.107	0.04 ± 0.01	ref.	0.04 ± 0.01	0.10	(-0.24;0.45)	0.556	0.969	0.04 ± 0.01	0.16	(-0.20;0.52)	0.392	0.641	0.339	0.598
Sphingomyelin C26:1	SM C26:1	0.10 ± 0.04	0.11	(-0.04;0.26)	0.150	0.310	0.10 ± 0.03	ref.	0.1 ± 0.1	0.14	(-0.21;0.49)	0.424	0.969	0.11 ± 0.03	0.29	(-0.07;0.66)	0.114	0.333	0.109	0.323
**HEXOSES**																				
Hexoses	H1	5574.0 ± 1481.3	0.06	(-0.09;0.21)	0.413	0.570	5503.9 ± 1166.4	ref.	5612.4 ± 1481.7	0.11	(-0.24;0.47)	0.533	0.969	5605.5 ± 1757.1	0.12	(-0.25;0.49)	0.514	0.718	0.500	0.730

			Carnivore pattern																	
			Continuous				Tertile 1		Tertile 2					Tertile 3						
**Metabolite**	**Abbreviation**	Mean ± SD [2]	β	95% CI [3]	*p* -value	*p* FDR [4]	Mean ± SD [2]	β	Mean ± SD [2]	β	95% CI [3]	*p* -value	*p* FDR [4]	Mean ± SD [2]	β	95% CI [3]	*p* -value	*p* FDR [4]	*p* trend	*p* trend FDR [4]
**ACYLCARNITINES**																				
Carnitine	C0	36.0 ± 7.3	0.09	(-0.06;0.24)	0.227	0.584	34.4 ± 7.7	ref.	35.9 ± 6.8	0.17	(-0.19;0.52)	0.356	0.915	37.6 ± 7.1	0.42	(0.06;0.79)	0.024	0.127	0.027	0.169
Acetylcarnitine	C2	6.0 ± 1.9	0.02	(-0.13;0.16)	0.824	0.917	5.7 ± 1.8	ref.	6.0 ± 1.7	0.06	(-0.29;0.41)	0.744	0.961	6.1 ± 2.1	0.12	(-0.25;0.48)	0.531	0.812	0.709	0.872
Propionylcarnitine	C3	0.5 ± 0.2	0.09	(-0.06;0.23)	0.237	0.599	0.4 ± 0.1	ref.	0.4 ± 0.1	0.00	(-0.34;0.35)	0.985	0.988	0.5 ± 0.2	0.42	(0.07;0.78)	0.021	0.116	0.035	0.185
Butyrylcarnitine	C4	0.2 ± 0.1	0.09	(-0.06;0.24)	0.225	0.584	0.2 ± 0.1	ref.	0.2 ± 0.1	0.17	(-0.19;0.52)	0.359	0.915	0.3 ± 0.1	0.33	(-0.04;0.69)	0.080	0.306	0.075	0.316
Valerylcarnitine	C5	0.2 ± 0.1	0.15	(0.02;0.29)	0.027	0.170	0.1 ± 0.1	ref.	0.2 ± 0.1	0.27	(-0.05;0.60)	0.099	0.915	0.2 ± 0.1	0.50	(0.16;0.83)	0.004	0.062	0.006	0.104
Decenoylcarnitine	C10:1	0.1 ± 0.1	0.07	(-0.08;0.21)	0.380	0.766	0.12 ± 0.05	ref.	0.13 ± 0.05	0.04	(-0.32;0.40)	0.827	0.961	0.1 ± 0.1	0.07	(-0.30;0.44)	0.706	0.888	0.692	0.872
Tetradecenoylcarnitine	C14:1	0.05 ± 0.02	-0.03	(-0.17;0.12)	0.730	0.901	0.05 ± 0.03	ref.	0.05 ± 0.02	-0.03	(-0.38;0.32)	0.845	0.961	0.05 ± 0.03	-0.13	(-0.49;0.24)	0.496	0.801	0.577	0.869
Tetradecadienylcarnitine	C14:2	0.02 ± 0.01	-0.04	(-0.18;0.10)	0.577	0.901	0.02 ± 0.01	ref.	0.02 ± 0.01	-0.13	(-0.47;0.22)	0.479	0.915	0.02 ± 0.01	-0.16	(-0.52;0.21)	0.399	0.716	0.466	0.781
Hexadecanoylcarnitine	C16	0.10 ± 0.03	0.01	(-0.14;0.15)	0.900	0.942	0.10 ± 0.03	ref.	0.10 ± 0.02	-0.01	(-0.36;0.34)	0.956	0.988	0.10 ± 0.03	-0.12	(-0.48;0.24)	0.508	0.801	0.586	0.869
Octadecanoylcarnitine	C18	0.05 ± 0.01	-0.01	(-0.16;0.13)	0.887	0.936	0.05 ± 0.01	ref.	0.05 ± 0.01	0.10	(-0.25;0.45)	0.589	0.915	0.05 ± 0.01	-0.10	(-0.47;0.26)	0.577	0.849	0.737	0.876
Octadecenoylcarnitine	C18:1	0.09 ± 0.03	-0.03	(-0.18;0.12)	0.666	0.901	0.09 ± 0.04	ref.	0.08 ± 0.03	-0.05	(-0.41;0.31)	0.780	0.961	0.08 ± 0.03	-0.07	(-0.44;0.31)	0.729	0.888	0.760	0.876
Octadecadienylcarnitine	C18:2	0.03 ± 0.01	0.03	(-0.12;0.18)	0.712	0.901	0.03 ± 0.02	ref.	0.04 ± 0.01	0.07	(-0.30;0.43)	0.724	0.961	0.03 ± 0.01	0.03	(-0.35;0.40)	0.888	0.974	0.847	0.884
**AMINO ACIDS**																				
Alanine	Ala	382.8 ± 100.7	-0.05	(-0.19;0.10)	0.550	0.901	378.1 ± 102.4	ref.	381.4 ± 103.1	-0.04	(-0.40;0.32)	0.820	0.961	389 ± 97.7	0.06	(-0.32;0.43)	0.769	0.920	0.826	0.879
Arginine	Arg	81.3 ± 23.5	0.13	(-0.02;0.27)	0.085	0.351	77.3 ± 22.5	ref.	80.8 ± 23.5	0.09	(-0.26;0.45)	0.613	0.915	85.9 ± 23.9	0.36	(-0.01;0.72)	0.057	0.245	0.078	0.318
Asparagine	Asn	43.5 ± 9.0	-0.02	(-0.16;0.13)	0.814	0.917	43.0 ± 9.0	ref.	44.2 ± 9.1	0.22	(-0.13;0.57)	0.214	0.915	43.2 ± 9.1	0.14	(-0.22;0.50)	0.449	0.743	0.364	0.718
Aspartate	Asp	11.8 ± 4.0	-0.14	(-0.28;0.01)	0.074	0.349	12.6 ± 4.3	ref.	11.1 ± 3.5	-0.28	(-0.64;0.07)	0.122	0.915	11.8 ± 4.0	-0.19	(-0.56;0.18)	0.318	0.656	0.266	0.604
Citrulline	Cit	36.5 ± 10.2	0.04	(-0.11;0.19)	0.572	0.901	36.1 ± 10.5	ref.	35.2 ± 9.5	-0.10	(-0.46;0.26)	0.592	0.915	38.1 ± 10.6	0.21	(-0.17;0.58)	0.280	0.610	0.326	0.671
Glutamine	Gln	696.5 ± 98.5	-0.04	(-0.18;0.10)	0.618	0.901	689.9 ± 95.5	ref.	703.8 ± 95.9	0.08	(-0.26;0.42)	0.633	0.932	695.8 ± 104.9	0.12	(-0.23;0.47)	0.504	0.801	0.643	0.869
Glutamate	Glu	80.0 ± 29.7	0.00	(-0.14;0.15)	0.979	0.979	77.9 ± 26.5	ref.	78.5 ± 28.4	-0.05	(-0.40;0.30)	0.784	0.961	83.6 ± 33.7	-0.01	(-0.38;0.35)	0.947	0.993	0.793	0.876
Glycine	Gly	215.6 ± 59.8	-0.08	(-0.21;0.06)	0.249	0.610	229.8 ± 70.8	ref.	212.1 ± 50.6	-0.12	(-0.45;0.20)	0.464	0.915	204.8 ± 54	-0.14	(-0.48;0.20)	0.424	0.728	0.258	0.601
Histidine	His	71.1 ± 12.4	0.07	(-0.08;0.21)	0.362	0.766	69.4 ± 13.1	ref.	72.2 ± 10.4	0.14	(-0.21;0.49)	0.433	0.915	71.8 ± 13.5	0.10	(-0.26;0.46)	0.573	0.849	0.537	0.869
Isoleucine	Ile	75.4 ± 23.9	0.12	(-0.02;0.26)	0.086	0.351	67.7 ± 17.8	ref.	76.8 ± 25.4	0.23	(-0.10;0.57)	0.175	0.915	81.6 ± 26.0	0.45	(0.1;0.80)	0.012	0.095	0.010	0.104
Leucine	Leu	133.8 ± 42.3	0.14	(0.00;0.28)	0.054	0.303	121.4 ± 34.3	ref.	137 ± 41.4	0.27	(-0.07;0.61)	0.126	0.915	143 ± 47.8	0.36	(0.00;0.71)	0.050	0.223	0.047	0.234
Lysine	Lys	193.5 ± 41.8	0.13	(-0.01;0.27)	0.069	0.342	182.9 ± 33.9	ref.	196 ± 46.1	0.20	(-0.15;0.54)	0.264	0.915	201.6 ± 42.8	0.41	(0.05;0.76)	0.025	0.128	0.028	0.169
Methionine	Met	20.20 ± 5.8	0.13	(-0.01;0.28)	0.065	0.335	18.8 ± 5.0	ref.	20.3 ± 6.2	0.23	(-0.11;0.57)	0.185	0.915	21.6 ± 5.9	0.44	(0.09;0.79)	0.016	0.102	0.011	0.104
Ornithine	Orn	71.3 ± 24.6	0.03	(-0.12;0.17)	0.720	0.901	70.7 ± 28.9	ref.	70.1 ± 24.3	0.08	(-0.28;0.43)	0.677	0.961	73.1 ± 19.9	0.32	(-0.04;0.69)	0.084	0.311	0.087	0.341
Phenylalanine	Phe	62.8 ± 13.6	0.09	(-0.05;0.23)	0.226	0.584	59.7 ± 11.6	ref.	63.7 ± 14.6	0.16	(-0.18;0.51)	0.347	0.915	64.9 ± 14.2	0.24	(-0.11;0.60)	0.179	0.490	0.122	0.408
Proline	Pro	242.6 ± 79.5	0.04	(-0.10;0.18)	0.573	0.901	234.9 ± 74.4	ref.	249.9 ± 85.7	0.12	(-0.22;0.47)	0.486	0.915	243 ± 78.6	0.08	(-0.28;0.44)	0.664	0.888	0.737	0.876
Serine	Ser	93 ± 20.3	0.10	(-0.04;0.25)	0.169	0.513	90.1 ± 19.2	ref.	94.3 ± 21.4	0.26	(-0.10;0.61)	0.157	0.915	94.7 ± 20.3	0.31	(-0.06;0.67)	0.101	0.348	0.075	0.316
Threonine	Thr	108.7 ± 27.7	0.18	(0.04;0.33)	0.012	0.118	103.5 ± 26.0	ref.	109.9 ± 29.5	0.34	(-0.01;0.68)	0.057	0.915	112.9 ± 27.1	0.45	(0.09;0.80)	0.015	0.102	0.009	0.104
Tryptophan	Trp	53.1 ± 11.0	0.18	(0.04;0.32)	0.013	0.118	49.8 ± 11.1	ref.	54.1 ± 10.6	0.35	(0.01;0.70)	0.048	0.915	55.5 ± 10.6	0.49	(0.13;0.85)	0.008	0.081	0.009	0.104
Tyrosine	Tyr	74.8 ± 24.1	0.10	(-0.05;0.24)	0.195	0.555	67.5 ± 19.9	ref.	76.0 ± 22.8	0.30	(-0.05;0.65)	0.095	0.915	80.7 ± 27.4	0.45	(0.09;0.81)	0.016	0.102	0.012	0.104
Valine	Val	242.1 ± 64.0	0.17	(0.03;0.31)	**0.019**	0.141	221.0 ± 52.8	ref.	245.3 ± 59.5	0.26	(-0.08;0.60)	0.134	0.915	259.9 ± 73	0.43	(0.08;0.78)	**0.018**	0.106	**0.018**	0.126
**BIOGENIC AMINES**																				
Creatinine	Creatinine	74.5 ± 17.4	0.05	(-0.06;0.17)	0.368	0.766	71.8 ± 16.2	ref.	73.3 ± 13.1	-0.10	(-0.38;0.19)	0.506	0.915	78.3 ± 21.3	0.00	(-0.29;0.30)	0.988	0.993	0.838	0.884
Kynurenine	Kynurenine	2.7 ± 0.7	0.03	(-0.11;0.17)	0.697	0.901	2.6 ± 0.9	ref.	2.6 ± 0.6	-0.19	(-0.52;0.15)	0.276	0.915	2.8 ± 0.7	0.02	(-0.33;0.37)	0.904	0.974	0.798	0.876
Sarcosine	Sarcosine	4.4 ± 1.1	0.10	(-0.05;0.24)	0.185	0.540	4.4 ± 1.3	ref.	4.4 ± 1.1	0.16	(-0.19;0.52)	0.373	0.915	4.4 ± 0.9	0.17	(-0.20;0.54)	0.361	0.677	0.437	0.751
Serotonin	Serotonin	0.8 ± 0.6	-0.05	(-0.19;0.10)	0.546	0.901	0.8 ± 0.6		0.8 ± 0.6	-0.02	(-0.37;0.34)	0.934	0.988	0.7 ± 0.5	-0.10	(-0.46;0.27)	0.608	0.867	0.788	0.876
Taurine	Taurine	120.9 ± 49.4	-0.05	(-0.19;0.10)	0.545	0.901	125.2 ± 48.9		120.1 ± 50.7	-0.03	(-0.39;0.33)	0.875	0.984	117.2 ± 49	-0.05	(-0.43;0.32)	0.777	0.921	0.768	0.876
Trans 4-Hydroxyproline	t4-OH-Pro	10.2 ± 5.0	0.11	(-0.03;0.26)	0.116	0.408	9.6 ± 5.3	ref.	10.20 ± 5.5	0.06	(-0.29;0.40)	0.746	0.961	10.7 ± 4.2	0.20	(-0.16;0.56)	0.284	0.610	0.318	0.666
Asymmetric dimethylarginine	ADMA	0.5 ± 0.1	-0.05	(-0.19;0.08)	0.418	0.788	0.5 ± 0.1	ref.	0.5 ± 0.1	-0.12	(-0.44;0.20)	0.459	0.915	0.5 ± 0.1	-0.03	(-0.36;0.30)	0.850	0.957	0.593	0.869
Symmetric dimethylarginine	SDMA	0.5 ± 0.1	-0.17	(-0.31;-0.04)	**0.013**	0.118	0.5 ± 0.1	ref.	0.5 ± 0.1	-0.16	(-0.49;0.17)	0.331	0.915	0.5 ± 0.1	-0.16	(-0.50;0.18)	0.358	0.677	0.251	0.601
**GLYCEROPHOSPHOLIPIDS**																				
Lysophosphatidylcholine (acyl) C16:0	lysoPC a C16:0	90.4 ± 20.1	0.08	(-0.07;0.23)	0.282	0.630	92.1 ± 21.2	ref.	90.7 ± 22.7	-0.05	(-0.42;0.31)	0.785	0.961	88.5 ± 15.9	-0.02	(-0.40;0.35)	0.909	0.974	0.654	0.869
Lysophosphatidylcholine (acyl) C16:1	lysoPC a C16:1	2.6 ± 0.9	-0.02	(-0.17;0.13)	0.832	0.917	2.7 ± 1.1	ref.	2.5 ± 0.9	-0.17	(-0.53;0.19)	0.366	0.915	2.5 ± 0.8	-0.09	(-0.47;0.28)	0.636	0.888	0.437	0.751
Lysophosphatidylcholine (acyl) C17:0	lysoPC a C17:0	1.6 ± 0.5	0.03	(-0.12;0.17)	0.730	0.901	1.7 ± 0.5	ref.	1.5 ± 0.5	-0.17	(-0.52;0.18)	0.334	0.915	1.5 ± 0.4	-0.07	(-0.43;0.29)	0.710	0.888	0.392	0.745
Lysophosphatidylcholine (acyl) C18:0	lysoPC a C18:0	26.4 ± 6.8	0.10	(-0.04;0.25)	0.162	0.504	27.2 ± 7.5	ref.	26.4 ± 7.5	-0.02	(-0.38;0.33)	0.895	0.986	25.4 ± 5.2	0.01	(-0.36;0.38)	0.956	0.993	0.776	0.876
Lysophosphatidylcholine (acyl) C18:1	lysoPC a C18:1	17.9 ± 5.4	-0.02	(-0.16;0.13)	0.823	0.917	18.8 ± 5.8	ref.	17.9 ± 5.9	-0.06	(-0.42;0.30)	0.740	0.961	17.1 ± 4.3	-0.07	(-0.43;0.30)	0.729	0.888	0.562	0.869
Lysophosphatidylcholine (acyl) C18:2	lysoPC a C18:2	29.2 ± 10.4	-0.01	(-0.15;0.14)	0.932	0.952	29.8 ± 10.1	ref.	29.8 ± 10.9	-0.01	(-0.35;0.34)	0.966	0.988	28.1 ± 10.1	-0.04	(-0.40;0.31)	0.809	0.930	0.619	0.869
Lysophosphatidylcholine (acyl) C20:3	lysoPC a C20:3	2.0 ± 0.7	-0.02	(-0.17;0.12)	0.770	0.901	2.2 ± 0.7	ref.	2.0 ± 0.8	-0.25	(-0.60;0.10)	0.158	0.915	1.9 ± 0.6	-0.25	(-0.61;0.11)	0.178	0.490	0.089	0.341
Lysophosphatidylcholine (acyl) C20:4	lysoPC a C20:4	5.9 ± 1.7	0.12	(-0.02;0.27)	0.098	0.365	6.0 ± 2.0	ref.	5.7 ± 1.6	-0.08	(-0.44;0.27)	0.650	0.947	6.1 ± 1.6	0.18	(-0.19;0.55)	0.333	0.677	0.591	0.869
Lysophosphatidylcholine (acyl) C24:0	lysoPC a C24:0	0.14 ± 0.03	0.11	(-0.02;0.24)	0.103	0.374	0.13 ± 0.04		0.13 ± 0.03	0.00	(-0.32;0.32)	0.988	0.988	0.14 ± 0.03	0.30	(-0.03;0.63)	0.079	0.306	0.172	0.490
Lysophosphatidylcholine (acyl) C28:1	lysoPC a C28:1	0.3 ± 0.1	-0.05	(-0.17;0.07)	0.443	0.825	0.3 ± 0.1	ref.	0.3 ± 0.1	-0.26	(-0.55;0.03)	0.084	0.915	0.3 ± 0.1	-0.20	(-0.49;0.10)	0.201	0.509	0.109	0.383
Phosphatidylcholine (diacyl) C28:1	PC aa C28:1	3.0 ± 0.8	-0.10	(-0.24;0.03)	0.143	0.491	3.1 ± 0.8	ref.	3.0 ± 0.7	-0.09	(-0.42;0.24)	0.584	0.915	2.9 ± 0.7	-0.09	(-0.43;0.25)	0.600	0.864	0.406	0.745
Phosphatidylcholine (diacyl) C30:0	PC aa C30:0	4.0 ± 1.3	-0.02	(-0.17;0.12)	0.766	0.901	4.1 ± 1.1	ref.	4.0 ± 1.3	-0.08	(-0.43;0.28)	0.679	0.961	4.0 ± 1.6	-0.03	(-0.40;0.34)	0.880	0.974	0.851	0.884
Phosphatidylcholine (diacyl) C32:0	PC aa C32:0	26.6 ± 5.5	0.02	(-0.13;0.16)	0.825	0.917	27 ± 4.9	ref.	26.0 ± 5.0	-0.10	(-0.45;0.24)	0.552	0.915	26.9 ± 6.5	0.09	(-0.27;0.44)	0.637	0.888	0.669	0.869

			Continuous				Tertile 1		Tertile 2					Tertile 3						
**Metabolite**	**Abbreviation**	Mean ± SD [2]	β	95% CI [3]	*p* -value	p_{FDR} [4]	Mean ± SD [2]	β	Mean ± SD [2]	β	95% CI [3]	*p* -value	p_{FDR} [4]	Mean ± SD [2]	β	95% CI [3]	*p* -value	p_{FDR} [4]	p_{trend}	$p_{trend\ FDR}$ [4]
Phosphatidylcholine (diacyl) C32:1	PC aa C32:1	32.0 ± 16.2	-0.05	(-0.20;0.10)	0.508	0.884	33 ± 15.4	ref.	30.8 ± 14.0	-0.13	(-0.49;0.23)	0.467	0.915	32.3 ± 18.8	-0.08	(-0.45;0.29)	0.667	0.888	0.641	0.869
Phosphatidylcholine (diacyl) C32:2	PC aa C32:2	8.3 ± 3.1	-0.10	(-0.25;0.04)	0.161	0.504	8.7 ± 3.0	ref.	8.6 ± 2.9	-0.05	(-0.40;0.30)	0.779	0.961	7.8 ± 3.4	-0.20	(-0.56;0.17)	0.287	0.610	0.198	0.520
Phosphatidylcholine (diacyl) C32:3	PC aa C32:3	0.9 ± 0.2	-0.02	(-0.16;0.11)	0.718	0.901	0.9 ± 0.2	ref.	0.9 ± 0.2	0.04	(-0.28;0.37)	0.807	0.961	0.9 ± 0.3	-0.02	(-0.36;0.31)	0.897	0.974	0.681	0.869
Phosphatidylcholine (diacyl) C34:1	PC aa C34:1	365.4 ± 82.8	-0.05	(-0.20;0.09)	0.464	0.840	371.8 ± 86.9	ref.	364.3 ± 77.0	-0.04	(-0.40;0.31)	0.815	0.961	360.20 ± 85.1	-0.07	(-0.44;0.29)	0.693	0.888	0.706	0.872
Phosphatidylcholine (diacyl) C34:2	PC aa C34:2	739.8 ± 128.1	-0.06	(-0.21;0.09)	0.405	0.776	751.7 ± 130.8	ref.	746.8 ± 117.6	0.00	(-0.36;0.36)	0.983	0.988	720.8 ± 135	-0.15	(-0.52;0.23)	0.441	0.743	0.423	0.751
Phosphatidylcholine (diacyl) C34:3	PC aa C34:3	30.1 ± 10.2	-0.04	(-0.18;0.11)	0.628	0.901	31.0 ± 10.9	ref.	31.0 ± 9.4	0.03	(-0.32;0.37)	0.882	0.984	28.4 ± 10.1	-0.15	(-0.51;0.21)	0.421	0.728	0.397	0.745
Phosphatidylcholine (diacyl) C34:4	PC aa C34:4	3.1 ± 1.2	0.04	(-0.10;0.18)	0.584	0.901	3.0 ± 1.1	ref.	3.1 ± 1.1	0.04	(-0.31;0.39)	0.810	0.961	3.1 ± 1.3	0.17	(-0.19;0.53)	0.357	0.677	0.574	0.869
Phosphatidylcholine (diacyl) C36:0	PC aa C36:0	3.2 ± 1.0	0.22	(0.09;0.36)	**0.002**	**0.029**	3.1 ± 1.0	ref.	3.1 ± 0.9	0.18	(-0.15;0.52)	0.281	0.915	3.5 ± 1.2	0.59	(0.25;0.94)	**0.001**	**0.025**	**0.003**	0.077
Phosphatidylcholine (diacyl) C36:1	PC aa C36:1	62.9 ± 17.8	-0.01	(-0.14;0.13)	0.938	0.952	64.3 ± 18.3	ref.	62.3 ± 15.8	-0.01	(-0.34;0.33)	0.975	0.988	62.2 ± 19.2	0.06	(-0.29;0.41)	0.726	0.888	0.749	0.876
Phosphatidylcholine (diacyl) C36:2	PC aa C36:2	384.7 ± 79.7	-0.02	(-0.16;0.12)	0.767	0.901	393.9 ± 81.8	ref.	387.1 ± 72.4	-0.02	(-0.36;0.32)	0.917	0.988	373.3 ± 84.2	-0.07	(-0.42;0.28)	0.701	0.888	0.631	0.869
Phosphatidylcholine (diacyl) C36:3	PC aa C36:3	192.6 ± 43.8	-0.06	(-0.20;0.08)	0.388	0.766	200.6 ± 42.2	ref.	192.9 ± 42.7	-0.18	(-0.51;0.16)	0.311	0.915	184.4 ± 45.7	-0.23	(-0.58;0.12)	0.193	0.507	0.135	0.430
Phosphatidylcholine (diacyl) C36:4	PC aa C36:4	291.5 ± 72.0	0.14	(0.00;0.28)	0.054	0.303	282.8 ± 72.9	ref.	285.8 ± 65.8	0.05	(-0.29;0.39)	0.762	0.961	305.8 ± 75.9	0.37	(0.02;0.72)	**0.042**	0.208	0.098	0.355
Phosphatidylcholine (diacyl) C36:5	PC aa C36:5	45.8 ± 27.8	0.09	(-0.05;0.24)	0.217	0.584	44.1 ± 25.5	ref.	44.3 ± 24.5	0.07	(-0.29;0.42)	0.713	0.961	48.8 ± 32.8	0.26	(-0.10;0.63)	0.162	0.476	0.181	0.495
Phosphatidylcholine (diacyl) C36:6	PC aa C36:6	1.5 ± 0.6	0.12	(-0.02;0.26)	0.097	0.365	1.5 ± 0.6	ref.	1.5 ± 0.7	0.10	(-0.24;0.45)	0.561	0.915	1.6 ± 0.6	0.29	(-0.07;0.65)	0.114	0.379	0.223	0.554
Phosphatidylcholine (diacyl) C38:0	PC aa C38:0	2.9 ± 0.9	0.32	(0.18;0.45)	**0.00001**	**0.001**	2.7 ± 0.9	ref.	2.8 ± 0.8	0.31	(-0.01;0.64)	0.060	0.915	3.1 ± 0.9	0.67	(0.33;1.01)	**0.0001**	**0.009**	**0.0002**	**0.020**
Phosphatidylcholine (diacyl) C38:3	PC aa C38:3	53.2 ± 13.6	0.04	(-0.09;0.18)	0.525	0.901	54.4 ± 11.7	ref.	52.3 ± 13	-0.19	(-0.51;0.13)	0.248	0.915	53.0 ± 15.9	-0.02	(-0.36;0.31)	0.893	0.974	0.676	0.869
Phosphatidylcholine (diacyl) C38:4	PC aa C38:4	131.5 ± 35.0	0.19	(0.06;0.32)	**0.004**	0.054	127.8 ± 34.4	ref.	126.6 ± 28.5	0.01	(-0.30;0.33)	0.931	0.988	140.20 ± 40	0.42	(0.1;0.75)	**0.012**	0.095	**0.036**	0.185
Phosphatidylcholine (diacyl) C38:5	PC aa C38:5	66.2 ± 19.0	0.09	(-0.04;0.23)	0.184	0.540	66.3 ± 17.8	ref.	64.8 ± 18.2	-0.01	(-0.34;0.32)	0.951	0.988	67.6 ± 21.1	0.22	(-0.12;0.57)	0.210	0.521	0.302	0.643
Phosphatidylcholine (diacyl) C38:6	PC aa C38:6	101.1 ± 33.6	0.27	(0.13;0.40)	**0.0002**	**0.006**	95.3 ± 31.1	ref.	100.1 ± 35.2	0.19	(-0.15;0.53)	0.283	0.915	107.9 ± 33.6	0.51	(0.16;0.86)	**0.005**	0.062	**0.012**	0.104
Phosphatidylcholine (diacyl) C40:2	PC aa C40:2	0.3 ± 0.1	0.01	(-0.13;0.15)	0.855	0.924	0.3 ± 0.1	ref.	0.3 ± 0.1	0.05	(-0.28;0.39)	0.753	0.961	0.3 ± 0.1	0.25	(-0.10;0.60)	0.164	0.476	0.175	0.490
Phosphatidylcholine (diacyl) C40:3	PC aa C40:3	0.4 ± 0.1	0.01	(-0.13;0.15)	0.909	0.944	0.4 ± 0.1	ref.	0.4 ± 0.1	-0.11	(-0.44;0.22)	0.515	0.915	0.4 ± 0.2	0.19	(-0.15;0.53)	0.275	0.610	0.349	0.697
Phosphatidylcholine (diacyl) C40:4	PC aa C40:4	3.0 ± 0.8	0.08	(-0.06;0.23)	0.274	0.623	3.0 ± 0.8	ref.	2.9 ± 0.7	-0.04	(-0.39;0.31)	0.834	0.961	3.1 ± 1	0.16	(-0.20;0.53)	0.375	0.688	0.508	0.840
Phosphatidylcholine (diacyl) C40:5	PC aa C40:5	8.3 ± 2.4	0.06	(-0.08;0.20)	0.394	0.766	8.2 ± 2.0	ref.	8.2 ± 2.3	-0.03	(-0.38;0.31)	0.844	0.961	8.4 ± 3	0.07	(-0.29;0.43)	0.710	0.888	0.774	0.876
Phosphatidylcholine (diacyl) C40:6	PC aa C40:6	28.3 ± 10.2	0.29	(0.16;0.43)	**0.00003**	**0.002**	26.8 ± 9.1	ref.	27.3 ± 10.2	0.10	(-0.23;0.43)	0.565	0.915	30.8 ± 10.9	0.50	(0.16;0.84)	**0.005**	0.062	**0.010**	0.104
Phosphatidylcholine (diacyl) C42:0	PC aa C42:0	0.3 ± 0.1	0.21	(0.08;0.34)	**0.002**	**0.035**	0.4 ± 0.1	ref.	0.3 ± 0.1	0.17	(-0.15;0.50)	0.299	0.915	0.3 ± 0.1	0.34	(0.00;0.68)	**0.050**	0.223	0.068	0.316
Phosphatidylcholine (diacyl) C42:1	PC aa C42:1	0.18 ± 0.05	0.23	(0.1;0.36)	**0.001**	**0.016**	0.2 ± 0.1	ref.	0.17 ± 0.04	0.12	(-0.20;0.45)	0.455	0.915	0.2 ± 0.1	0.45	(0.12;0.78)	**0.009**	0.088	**0.018**	0.126
Phosphatidylcholine (diacyl) C42:2	PC aa C42:2	0.16 ± 0.04	0.13	(-0.01;0.27)	0.076	0.349	0.15 ± 0.04	ref.	0.16 ± 0.04	0.22	(-0.11;0.56)	0.190	0.915	0.16 ± 0.05	0.50	(0.15;0.84)	**0.006**	0.062	**0.010**	0.104
Phosphatidylcholine (diacyl) C42:4	PC aa C42:4	0.10 ± 0.02	0.04	(-0.11;0.18)	0.620	0.901	0.10 ± 0.02	ref.	0.10 ± 0.02	0.04	(-0.32;0.39)	0.847	0.961	0.11 ± 0.02	0.24	(-0.12;0.61)	0.194	0.507	0.260	0.601
Phosphatidylcholine (diacyl) C42:5	PC aa C42:5	0.3 ± 0.1	0.03	(-0.11;0.18)	0.658	0.901	0.3 ± 0.1	ref.	0.2 ± 0.1	-0.12	(-0.48;0.23)	0.489	0.915	0.3 ± 0.1	0.17	(-0.20;0.53)	0.364	0.677	0.404	0.745
Phosphatidylcholine (diacyl) C42:6	PC aa C42:6	0.4 ± 0.1	0.02	(-0.12;0.17)	0.737	0.901	0.4 ± 0.1	ref.	0.4 ± 0.1	-0.02	(-0.37;0.33)	0.916	0.988	0.4 ± 0.2	0.21	(-0.15;0.58)	0.244	0.564	0.283	0.622
Phosphatidylcholine (acyl-alkyl) C30:0	PC ae C30:0	0.3 ± 0.1	-0.06	(-0.20;0.08)	0.391	0.766	0.4 ± 0.1	ref.	0.3 ± 0.1	-0.15	(-0.49;0.19)	0.375	0.915	0.3 ± 0.1	-0.20	(-0.55;0.16)	0.276	0.610	0.209	0.527
Phosphatidylcholine (acyl-alkyl) C30:2	PC ae C30:2	0.08 ± 0.02	0.01	(-0.11;0.14)	0.835	0.917	0.09 ± 0.02	ref.	0.08 ± 0.02	-0.10	(-0.40;0.20)	0.519	0.915	0.08 ± 0.02	-0.04	(-0.35;0.27)	0.812	0.930	0.541	0.869
Phosphatidylcholine (acyl-alkyl) C32:1	PC ae C32:1	5.3 ± 1.2	-0.03	(-0.17;0.11)	0.657	0.901	5.4 ± 1.2	ref.	5.1 ± 1	-0.10	(-0.44;0.24)	0.556	0.915	5.3 ± 1.4	0.08	(-0.27;0.43)	0.668	0.888	0.812	0.877
Phosphatidylcholine (acyl-alkyl) C32:2	PC ae C32:2	1.4 ± 0.4	-0.01	(-0.15;0.12)	0.851	0.924	1.4 ± 0.4	ref.	1.3 ± 0.3	-0.11	(-0.43;0.22)	0.511	0.915	1.4 ± 0.4	0.20	(-0.13;0.54)	0.241	0.564	0.400	0.745
Phosphatidylcholine (acyl-alkyl) C34:0	PC ae C34:0	2.5 ± 0.6	0.00	(-0.14;0.14)	0.976	0.979	2.5 ± 0.6	ref.	2.4 ± 0.6	-0.09	(-0.43;0.25)	0.601	0.915	2.4 ± 0.7	0.00	(-0.35;0.36)	0.990	0.993	0.939	0.960
Phosphatidylcholine (acyl-alkyl) C34:1	PC ae C34:1	18.0 ± 3.9	-0.10	(-0.23;0.03)	0.147	0.491	19.1 ± 4.2	ref.	17.7 ± 3.3	-0.12	(-0.45;0.20)	0.458	0.915	17.3 ± 3.9	-0.16	(-0.50;0.18)	0.361	0.677	0.341	0.693
Phosphatidylcholine (acyl-alkyl) C34:2	PC ae C34:2	20.2 ± 5.3	0.03	(-0.11;0.17)	0.690	0.901	20.4 ± 5.5	ref.	20.9 ± 5.1	0.19	(-0.15;0.52)	0.275	0.915	19.4 ± 5.3	0.05	(-0.30;0.39)	0.797	0.930	0.811	0.877
Phosphatidylcholine (acyl-alkyl) C34:3	PC ae C34:3	13.1 ± 4.0	0.03	(-0.11;0.17)	0.679	0.901	13.2 ± 4.3	ref.	13.6 ± 3.8	0.18	(-0.16;0.51)	0.310	0.915	12.6 ± 3.8	0.06	(-0.28;0.41)	0.720	0.888	0.797	0.876
Phosphatidylcholine (acyl-alkyl) C36:0	PC ae C36:0	1.2 ± 0.3	0.06	(-0.09;0.20)	0.452	0.830	1.1 ± 0.3	ref.	1.2 ± 0.3	0.12	(-0.24;0.47)	0.522	0.915	1.2 ± 0.4	0.23	(-0.13;0.60)	0.218	0.531	0.149	0.465
Phosphatidylcholine (acyl-alkyl) C36:1	PC ae C36:1	11.5 ± 2.7	-0.08	(-0.21;0.06)	0.250	0.610	12.2 ± 3.2	ref.	11.2 ± 2.2	-0.13	(-0.45;0.20)	0.455	0.915	11 ± 2.4	-0.10	(-0.44;0.24)	0.576	0.849	0.437	0.751
Phosphatidylcholine (acyl-alkyl) C36:2	PC ae C36:2	21.7 ± 5.4	-0.07	(-0.21;0.06)	0.272	0.623	23.3 ± 6.1	ref.	21.5 ± 4.8	-0.12	(-0.44;0.20)	0.472	0.915	20.3 ± 4.8	-0.20	(-0.53;0.13)	0.239	0.564	0.156	0.473
Phosphatidylcholine (acyl-alkyl) C36:3	PC ae C36:3	11.4 ± 2.8	0.03	(-0.10;0.17)	0.641	0.901	11.6 ± 3.0	ref.	11.7 ± 2.7	0.14	(-0.19;0.47)	0.404	0.915	10.9 ± 2.8	0.01	(-0.33;0.35)	0.950	0.993	0.982	0.990
Phosphatidylcholine (acyl-alkyl) C36:4	PC ae C36:4	26.3 ± 6.4	0.24	(0.1;0.38)	**0.001**	**0.018**	24.8 ± 6.0	ref.	26.4 ± 6.2	0.25	(-0.10;0.59)	0.159	0.915	27.5 ± 6.7	0.52	(0.16;0.87)	**0.005**	0.062	**0.012**	0.104
Phosphatidylcholine (acyl-alkyl) C36:5	PC ae C36:5	18.3 ± 5.0	0.18	(0.04;0.32)	**0.011**	0.118	17.2 ± 4.3	ref.	17.9 ± 4.5	0.19	(-0.14;0.51)	0.263	0.915	19.8 ± 5.7	0.66	(0.32;0.99)	**0.0002**	**0.009**	**0.001**	0.052
Phosphatidylcholine (acyl-alkyl) C38:0	PC ae C38:0	2.5 ± 0.8	0.16	(0.02;0.29)	**0.029**	0.177	2.4 ± 0.7	ref.	2.5 ± 0.8	0.22	(-0.11;0.56)	0.193	0.915	2.6 ± 0.9	0.44	(0.1;0.79)	**0.013**	0.095	**0.031**	0.174
Phosphatidylcholine (acyl-alkyl) C38:2	PC ae C38:2	2.2 ± 0.6	-0.03	(-0.17;0.10)	0.634	0.901	2.3 ± 0.6	ref.	2.1 ± 0.5	-0.16	(-0.49;0.17)	0.350	0.915	2.1 ± 0.5	-0.04	(-0.39;0.30)	0.804	0.930	0.569	0.869
Phosphatidylcholine (acyl-alkyl) C38:3	PC ae C38:3	4.8 ± 1.1	-0.04	(-0.17;0.10)	0.603	0.901	5.1 ± 1.2	ref.	4.6 ± 1.0	-0.32	(-0.64;-0.01)	**0.046**	0.915	4.5 ± 1	-0.24	(-0.57;0.09)	0.154	0.468	0.076	0.316
Phosphatidylcholine (acyl-alkyl) C38:4	PC ae C38:4	17.2 ± 3.6	0.16	(0.03;0.30)	**0.020**	0.141	17.2 ± 4.0	ref.	17.0 ± 3.2	0.10	(-0.23;0.44)	0.550	0.915	17.5 ± 3.7	0.33	(-0.02;0.67)	0.064	0.267	0.160	0.473
Phosphatidylcholine (acyl-alkyl) C38:5	PC ae C38:5	21.9 ± 4.7	0.20	(0.06;0.34)	**0.004**	0.054	21.2 ± 4.6	ref.	21.8 ± 4.3	0.24	(-0.09;0.57)	0.149	0.915	22.9 ± 4.9	0.58	(0.24;0.92)	**0.001**	**0.025**	**0.003**	0.077
Phosphatidylcholine (acyl-alkyl) C38:6	PC ae C38:6	9.2 ± 2.6	0.26	(0.12;0.39)	**0.0002**	**0.006**	8.7 ± 2.4	ref.	9.1 ± 2.5	0.24	(-0.08;0.56)	0.144	0.915	9.9 ± 2.8	0.68	(0.35;1.01)	**0.0001**	**0.009**	**0.0003**	**0.020**
Phosphatidylcholine (acyl-alkyl) C40:1	PC ae C40:1	1.1 ± 0.3	0.17	(0.03;0.31)	**0.018**	0.141	1.1 ± 0.3	ref.	1.1 ± 0.3	0.09	(-0.25;0.43)	0.614	0.915	1.2 ± 0.3	0.51	(0.17;0.86)	**0.004**	0.062	**0.015**	0.120
Phosphatidylcholine (acyl-alkyl) C40:2	PC ae C40:2	1.6 ± 0.4	-0.02	(-0.15;0.12)	0.819	0.917	1.6 ± 0.4	ref.	1.6 ± 0.4	0.09	(-0.24;0.42)	0.595	0.915	1.6 ± 0.5	0.15	(-0.20;0.49)	0.401	0.716	0.466	0.781
Phosphatidylcholine (acyl-alkyl) C40:3	PC ae C40:3	0.8 ± 0.2	-0.01	(-0.14;0.12)	0.880	0.936	0.9 ± 0.2	ref.	0.8 ± 0.2	-0.06	(-0.37;0.26)	0.712	0.961	0.8 ± 0.2	-0.03	(-0.36;0.29)	0.848	0.957	0.662	0.869
Phosphatidylcholine (acyl-alkyl) C40:4	PC ae C40:4	2.2 ± 0.4	0.13	(-0.01;0.27)	0.064	0.335	2.2 ± 0.5	ref.	2.2 ± 0.4	0.17	(-0.16;0.50)	0.314	0.915	2.2 ± 0.5	0.27	(-0.07;0.62)	0.120	0.383	0.162	0.473
Phosphatidylcholine (acyl-alkyl) C40:5	PC ae C40:5	3.4 ± 0.7	0.12	(-0.02;0.25)	0.090	0.353	3.4 ± 0.7	ref.	3.3 ± 0.6	0.14	(-0.19;0.46)	0.420	0.915	3.4 ± 0.8	0.26	(-0.08;0.60)	0.139	0.433	0.189	0.508
Phosphatidylcholine (acyl-alkyl) C40:6	PC ae C40:6	4.8 ± 1.2	0.26	(0.12;0.39)	**0.0002**	**0.006**	4.7 ± 1.3	ref.	4.7 ± 1.1	0.19	(-0.13;0.51)	0.253	0.915	4.9 ± 1.2	0.51	(0.18;0.85)	**0.003**	0.062	**0.009**	0.104
Phosphatidylcholine (acyl-alkyl) C42:1	PC ae C42:1	0.3 ± 0.1	0.12	(-0.01;0.26)	0.082	0.351	0.3 ± 0.1	ref.	0.3 ± 0.1	-0.05	(-0.38;0.28)	0.756	0.961	0.3 ± 0.1	0.27	(-0.07;0.62)	0.116	0.379	0.289	0.625
Phosphatidylcholine (acyl-alkyl) C42:2	PC ae C42:2	0.4 ± 0.1	0.06	(-0.07;0.19)	0.391	0.766	0.4 ± 0.1	ref.	0.4 ± 0.1	0.16	(-0.16;0.49)	0.322	0.915	0.4 ± 0.1	0.24	(-0.10;0.57)	0.167	0.477	0.202	0.520
Phosphatidylcholine (acyl-alkyl) C42:3	PC ae C42:3	0.6 ± 0.1	0.08	(-0.06;0.22)	0.2565	0.614	0.6 ± 0.1	ref.	0.6 ± 0.1	0.14	(-0.19;0.47)	0.4141	0.915	0.5 ± 0.1	0.23	(-0.12;0.57)	0.1969	0.507	0.270	0.604
Phosphatidylcholine (acyl-alkyl) C42:4	PC ae C42:4	0.7 ± 0.2	0.05	(-0.09;0.19)	0.500	0.881	0.7 ± 0.2	ref.	0.7 ± 0.1	0.15	(-0.19;0.48)	0.388	0.915	0.7 ± 0.2	0.05	(-0.29;0.40)	0.763	0.920	0.786	0.876
Phosphatidylcholine (acyl-alkyl) C42:5	PC ae C42:5	1.5 ± 0.3	0.16	(0.03;0.30)	**0.020**	0.141	1.5 ± 0.3	ref.	1.5 ± 0.3	0.22	(-0.11;0.56)	0.197	0.915	1.5 ± 0.4	0.36	(0.01;0.70)	**0.046**	0.221	0.072	0.316
Phosphatidylcholine (acyl-alkyl) C44:3	PC ae C44:3	0.07 ± 0.02	0.08	(-0.06;0.21)	0.266	0.623	0.07 ± 0.02	ref.	0.07 ± 0.02	0.10	(-0.23;0.42)	0.555	0.915	0.07 ± 0.02	0.41	(0.07;0.74)	**0.018**	0.106	**0.027**	0.169
Phosphatidylcholine (acyl-alkyl) C44:4	PC ae C44:4	0.2 ± 0.1	0.01	(-0.13;0.15)	0.920	0.948	0.2 ± 0.1	ref.	0.2 ± 0.1	0.01	(-0.33;0.35)	0.971	0.988	0.2 ± 0.1	-0.01	(-0.36;0.35)	0.978	0.993	0.882	0.909
Phosphatidylcholine (acyl-alkyl) C44:5	PC ae C44:5	1.0 ± 0.3	0.13	(-0.02;0.27)	0.082	0.351	1.0 ± 0.3	ref.	1.0 ± 0.2	0.30	(-0.05;0.64)	0.094	0.915	1.0 ± 0.3	0.31	(-0.05;0.67)	0.089	0.322	0.092	0.343
Phosphatidylcholine (acyl-alkyl) C44:6	PC ae C44:6	0.8 ± 0.2	0.15	(0.02;0.29)	**0.026**	0.170	0.8 ± 0.2	ref.	0.8 ± 0.2	0.17	(-0.16;0.50)	0.318	0.915	0.8 ± 0.3	0.32	(-0.03;0.66)	0.072	0.293	0.113	0.390

			Continuous				Tertile 1		Tertile 2					Tertile 3						
**Metabolite**	**Abbreviation**	Mean ± SD [2]	β	95% CI [3]	*p* -value	p_{FDR} [4]	Mean ± SD [2]	β	Mean ± SD [2]	β	95% CI [3]	*p* -value	p_{FDR} [4]	Mean ± SD [2]	β	95% CI [3]	*p* -value	p_{FDR} [4]	p_{trend}	$p_{trend\ FDR}$ [4]
**SPHINGOLIPIDS**																				
Hydroxysphingomyelin C14:1	SM OH C14:1	4.0 ± 1.0	-0.09	(-0.21;0.04)	0.162	0.504	4.2 ± 1.2	ref.	4.0 ± 0.9	-0.04	(-0.34;0.27)	0.819	0.961	3.8 ± 1.0	-0.06	(-0.37;0.26)	0.710	0.888	0.596	0.869
Hydroxysphingomyelin C16:1	SM OH C16:1	1.9 ± 0.5	-0.06	(-0.19;0.07)	0.338	0.731	1.9 ± 0.5	ref.	1.8 ± 0.4	0.02	(-0.30;0.34)	0.898	0.986	1.8 ± 0.5	0.01	(-0.31;0.34)	0.932	0.991	0.998	0.998
Hydroxysphingomyelin C22:1	SM OH C22:1	4.4 ± 1.1	-0.02	(-0.15;0.11)	0.768	0.901	4.5 ± 1.1	ref.	4.5 ± 1.0	0.17	(-0.15;0.49)	0.295	0.915	4.2 ± 1.0	0.07	(-0.26;0.40)	0.675	0.888	0.794	0.876
Hydroxysphingomyelin C22:2	SM OH C22:2	3.3 ± 0.9	-0.02	(-0.14;0.11)	0.771	0.901	3.4 ± 1.0	ref.	3.3 ± 0.8	0.15	(-0.15;0.45)	0.330	0.915	3.2 ± 0.9	0.12	(-0.19;0.43)	0.448	0.743	0.606	0.869
Hydroxysphingomyelin C24:1	SM OH C24:1	0.3 ± 0.1	0.02	(-0.12;0.16)	0.773	0.901	0.3 ± 0.1	ref.	0.3 ± 0.1	0.17	(-0.17;0.51)	0.329	0.915	0.3 ± 0.1	0.11	(-0.24;0.47)	0.527	0.812	0.680	0.869
Sphingomyelin C16:0	SM C16:0	67.2 ± 13.6	-0.02	(-0.15;0.11)	0.714	0.901	68.1 ± 12.4	ref.	67.7 ± 12.3	0.12	(-0.20;0.43)	0.471	0.915	65.8 ± 15.9	0.10	(-0.22;0.43)	0.533	0.812	0.663	0.869
Sphingomyelin C16:1	SM C16:1	9.5 ± 2.2	0.03	(-0.09;0.16)	0.605	0.901	9.5 ± 2.2	ref.	9.6 ± 2.1	0.09	(-0.21;0.39)	0.547	0.915	9.3 ± 2.3	0.13	(-0.18;0.44)	0.410	0.722	0.709	0.872
Sphingomyelin C18:0	SM C18:0	12.0 ± 3.3	-0.03	(-0.17;0.10)	0.638	0.901	12.1 ± 3.4	ref.	12.0 ± 2.6	0.10	(-0.23;0.43)	0.538	0.915	12 ± 3.8	0.09	(-0.25;0.44)	0.589	0.858	0.619	0.869
Sphingomyelin C18:1	SM C18:1	5.7 ± 1.6	0.03	(-0.10;0.16)	0.662	0.901	5.6 ± 1.7	ref.	5.7 ± 1.4	0.15	(-0.16;0.46)	0.349	0.915	5.7 ± 1.8	0.17	(-0.15;0.49)	0.299	0.626	0.415	0.751
Sphingomyelin C20:2	SM C20:2	0.2 ± 0.1	-0.08	(-0.21;0.05)	0.218	0.584	0.2 ± 0.1	ref.	0.14 ± 0.05	-0.19	(-0.50;0.13)	0.249	0.915	0.1 ± 0.1	-0.16	(-0.48;0.17)	0.351	0.677	0.258	0.601
Sphingomyelin C24:0	SM C24:0	6.1 ± 1.4	0.03	(-0.11;0.17)	0.652	0.901	6.1 ± 1.4	ref.	6.4 ± 1.4	0.25	(-0.08;0.59)	0.140	0.915	5.9 ± 1.4	0.12	(-0.22;0.47)	0.491	0.801	0.614	0.869
Sphingomyelin C24:1	SM C24:1	13.9 ± 3.4	0.04	(-0.10;0.18)	0.566	0.901	13.8 ± 3.2	ref.	13.9 ± 2.8	0.24	(-0.09;0.57)	0.163	0.915	13.9 ± 4.2	0.29	(-0.05;0.64)	0.097	0.340	0.132	0.430
Sphingomyelin C26:0	SM C26:0	0.04 ± 0.01	-0.05	(-0.19;0.09)	0.495	0.881	0.04 ± 0.01	ref.	0.04 ± 0.01	0.20	(-0.15;0.54)	0.272	0.915	0.03 ± 0.01	0.00	(-0.36;0.36)	0.993	0.993	0.822	0.879
Sphingomyelin C26:1	SM C26:1	0.10 ± 0.04	0.08	(-0.07;0.22)	0.300	0.660	0.10 ± 0.03	ref.	0.10 ± 0.03	0.09	(-0.25;0.44)	0.603	0.915	0.1 ± 0.1	0.46	(0.1;0.82)	**0.013**	0.095	**0.029**	0.169
**HEXOSES**																				
Hexoses	H1	5574.0 ± 1481.3	-0.01	(-0.16;0.13)	0.868	0.931	5496.6 ± 1445.1	ref.	5583.0 ± 1605.3	-0.06	(-0.42;0.29)	0.721	0.961	5642.3 ± 1405.3	0.00	(-0.36;0.37)	0.979	0.993	0.951	0.965

			Prudent pattern																	
			Continuous				Tertile 1		Tertile 2					Tertile 3						
**Metabolite**	**Abbreviation**	Mean ± SD [2]	β	95% CI [3]	*p* -value	p_{FDR} [4]	Mean ± SD [2]	β	Mean ± SD [2]	β	95% CI [3]	*p* -value	p_{FDR} [4]	Mean ± SD [2]	β	95% CI [3]	*p* -value	p_{FDR} [4]	p_{trend}	$p_{trend\ FDR}$ [4]
**ACYLCARNITINES**																				
Carnitine	C0	36.0 ± 7.3	-0.05	(-0.22;0.13)	0.606	0.882	37.3 ± 7.8	ref.	35.4 ± 7.3	-0.13	(-0.49;0.23)	0.476	0.907	35.2 ± 6.7	-0.06	(-0.44;0.31)	0.738	0.875	0.712	0.868
Acetylcarnitine	C2	6.0 ± 1.9	-0.10	(-0.27;0.07)	0.250	0.598	6.2 ± 2	ref.	6.0 ± 2.2	-0.10	(-0.45;0.26)	0.600	0.907	5.7 ± 1.5	-0.16	(-0.53;0.21)	0.410	0.686	0.338	0.604
Propionylcarnitine	C3	0.5 ± 0.2	0.01	(-0.16;0.18)	0.905	0.947	0.5 ± 0.2	ref.	0.5 ± 0.1	0.13	(-0.22;0.49)	0.467	0.907	0.5 ± 0.2	0.12	(-0.25;0.49)	0.537	0.786	0.497	0.754
Butyrylcarnitine	C4	0.2 ± 0.1	0.04	(-0.14;0.21)	0.678	0.908	0.2 ± 0.1	ref.	0.2 ± 0.1	0.06	(-0.30;0.42)	0.747	0.907	0.2 ± 0.1	0.08	(-0.29;0.46)	0.672	0.849	0.664	0.845
Valerylcarnitine	C5	0.2 ± 0.1	0.02	(-0.14;0.18)	0.811	0.947	0.2 ± 0.1	ref.	0.2 ± 0.1	0.08	(-0.26;0.41)	0.663	0.907	0.16 ± 0.05	0.12	(-0.23;0.47)	0.498	0.759	0.529	0.775
Decenoylcarnitine	C10:1	0.1 ± 0.1	0.01	(-0.16;0.18)	0.875	0.947	0.1 ± 0.1	ref.	0.1 ± 0.1	0.30	(-0.05;0.66)	0.097	0.728	0.1 ± 0.1	-0.03	(-0.40;0.34)	0.880	0.936	0.902	0.960
Tetradecenoylcarnitine	C14:1	0.05 ± 0.02	-0.12	(-0.28;0.05)	0.178	0.523	0.06 ± 0.03	ref.	0.06 ± 0.03	-0.06	(-0.41;0.30)	0.748	0.907	0.05 ± 0.02	-0.22	(-0.59;0.15)	0.246	0.522	0.255	0.542
Tetradecadienylcarnitine	C14:2	0.02 ± 0.01	-0.11	(-0.27;0.06)	0.214	0.531	0.03 ± 0.01	ref.	0.02 ± 0.01	0.03	(-0.32;0.38)	0.875	0.938	0.02 ± 0.01	-0.21	(-0.57;0.16)	0.267	0.537	0.274	0.562
Hexadecanoylcarnitine	C16	0.10 ± 0.03	-0.27	(-0.44;-0.11)	**0.001**	0.056	0.11 ± 0.03	ref.	0.10 ± 0.03	-0.39	(-0.74;-0.05)	0.027	0.685	0.10 ± 0.02	-0.56	(-0.92;-0.20)	0.003	0.091	0.003	0.098
Octadecanoylcarnitine	C18	0.05 ± 0.01	-0.23	(-0.40;-0.07)	**0.006**	0.098	0.05 ± 0.01	ref.	0.05 ± 0.01	-0.32	(-0.67;0.03)	0.079	0.728	0.05 ± 0.01	-0.47	(-0.84;-0.11)	0.012	0.168	0.013	0.188
Octadecenoylcarnitine	C18:1	0.09 ± 0.03	-0.28	(-0.45;-0.11)	**0.001**	0.056	0.09 ± 0.03	ref.	0.09 ± 0.03	-0.21	(-0.57;0.15)	0.262	0.907	0.08 ± 0.03	-0.52	(-0.90;-0.15)	0.007	0.111	0.008	0.131
Octadecadienylcarnitine	C18:2	0.03 ± 0.01	-0.28	(-0.45;-0.11)	**0.002**	0.056	0.04 ± 0.02	ref.	0.04 ± 0.01	-0.05	(-0.41;0.31)	0.790	0.907	0.03 ± 0.01	-0.54	(-0.91;-0.17)	0.005	0.091	0.006	0.118
**AMINO ACIDS**																				
Alanine	Ala	382.8 ± 100.7	-0.14	(-0.31;0.03)	0.117	0.471	389.8 ± 99.7	ref.	396.1 ± 112.8	0.02	(-0.35;0.38)	0.929	0.944	362.6 ± 86.1	-0.25	(-0.63;0.13)	0.202	0.480	0.242	0.542
Arginine	Arg	81.3 ± 23.5	0.15	(-0.02;0.32)	0.078	0.388	79.2 ± 24.2	ref.	79.1 ± 20.2	0.16	(-0.20;0.52)	0.385	0.907	85.7 ± 25.4	0.36	(-0.01;0.73)	0.061	0.315	0.070	0.350
Asparagine	Asn	43.5 ± 9.0	0.04	(-0.13;0.20)	0.675	0.908	43.0 ± 8.6	ref.	43.8 ± 9.2	0.06	(-0.30;0.41)	0.752	0.907	43.6 ± 9.5	0.01	(-0.35;0.38)	0.950	0.968	0.911	0.961
Aspartate	Asp	11.8 ± 4.0	-0.12	(-0.30;0.05)	0.167	0.517	12.1 ± 3.5	ref.	12.3 ± 4.5	-0.06	(-0.42;0.30)	0.727	0.907	11 ± 3.8	-0.46	(-0.83;-0.08)	0.017	0.195	0.022	0.199
Citrulline	Cit	36.5 ± 10.2	-0.08	(-0.25;0.10)	0.387	0.720	36.2 ± 8.9	ref.	39.3 ± 10.1	0.22	(-0.13;0.58)	0.221	0.907	33.9 ± 11	-0.33	(-0.70;0.04)	0.081	0.327	0.118	0.369
Glutamine	Gln	696.5 ± 98.5	-0.06	(-0.22;0.11)	0.499	0.787	699.5 ± 108	ref.	697 ± 99	0.10	(-0.24;0.45)	0.565	0.907	693.1 ± 89	-0.03	(-0.39;0.33)	0.857	0.936	0.816	0.912
Glutamate	Glu	80.0 ± 29.7	0.00	(-0.17;0.17)	0.980	0.983	85.8 ± 30.9	ref.	78.5 ± 29.7	-0.21	(-0.56;0.15)	0.260	0.907	75.6 ± 27.8	-0.27	(-0.64;0.10)	0.161	0.431	0.153	0.433
Glycine	Gly	215.6 ± 59.8	-0.21	(-0.36;-0.05)	**0.010**	0.113	218.6 ± 64	ref.	215.8 ± 63.9	-0.09	(-0.42;0.24)	0.584	0.907	212.4 ± 51.3	-0.30	(-0.65;0.04)	0.085	0.327	0.062	0.329
Histidine	His	71.1 ± 12.4	0.03	(-0.13;0.20)	0.696	0.919	71.6 ± 11.9	ref.	69.9 ± 12.3	-0.02	(-0.37;0.33)	0.917	0.944	71.8 ± 13.2	0.12	(-0.24;0.49)	0.514	0.774	0.500	0.754
Isoleucine	Ile	75.4 ± 23.9	0.08	(-0.09;0.24)	0.364	0.707	75.4 ± 18.7	ref.	73.6 ± 27.4	-0.06	(-0.41;0.28)	0.713	0.907	77.1 ± 25.2	0.22	(-0.14;0.58)	0.229	0.511	0.253	0.542
Leucine	Leu	133.8 ± 42.3	0.13	(-0.04;0.30)	0.125	0.471	133.3 ± 33.7	ref.	129.5 ± 46	-0.02	(-0.37;0.32)	0.894	0.944	138.6 ± 46.2	0.31	(-0.06;0.67)	0.100	0.338	0.116	0.369
Lysine	Lys	193.5 ± 41.8	0.17	(0.00;0.33)	0.050	0.320	191.8 ± 37.6	ref.	185.2 ± 38.9	-0.05	(-0.39;0.30)	0.788	0.907	203.4 ± 46.8	0.38	(0.02;0.74)	0.039	0.263	0.051	0.329
Methionine	Met	20.20 ± 5.8	0.11	(-0.06;0.28)	0.195	0.528	19.8 ± 5.7	ref.	19.9 ± 5.4	0.00	(-0.35;0.35)	0.998	0.998	21.0 ± 6.3	0.34	(-0.02;0.70)	0.068	0.327	0.058	0.329
Ornithine	Orn	71.3 ± 24.6	-0.14	(-0.31;0.03)	0.111	0.471	74.7 ± 26.8	ref.	71.6 ± 24.4	-0.14	(-0.49;0.22)	0.452	0.907	67.6 ± 22.2	-0.38	(-0.75;-0.01)	0.045	0.263	0.050	0.329
Phenylalanine	Phe	62.8 ± 13.6	-0.03	(-0.20;0.13)	0.718	0.934	63.1 ± 11.7	ref.	62.1 ± 14.3	-0.07	(-0.41;0.28)	0.711	0.907	63.1 ± 14.8	0.03	(-0.33;0.39)	0.863	0.936	0.815	0.912
Proline	Pro	242.6 ± 79.5	0.06	(-0.11;0.22)	0.495	0.787	238.3 ± 76.6	ref.	243.8 ± 85.3	0.09	(-0.26;0.45)	0.602	0.907	245.7 ± 77.5	0.18	(-0.19;0.54)	0.349	0.623	0.398	0.683
Serine	Ser	93 ± 20.3	-0.06	(-0.24;0.11)	0.461	0.774	94.0 ± 20.3	ref.	90.5 ± 22.3	-0.27	(-0.63;0.09)	0.146	0.781	94.6 ± 18.2	-0.04	(-0.41;0.34)	0.841	0.936	0.835	0.922
Threonine	Thr	108.7 ± 27.7	0.00	(-0.16;0.17)	0.960	0.982	108.0 ± 29.7	ref.	111.3 ± 28.5	0.06	(-0.29;0.42)	0.734	0.907	106.9 ± 25	0.06	(-0.31;0.43)	0.745	0.875	0.659	0.845
Tryptophan	Trp	53.1 ± 11.0	0.18	(0.01;0.35)	**0.036**	0.252	52.5 ± 12.2	ref.	52.1 ± 10.0	0.13	(-0.23;0.48)	0.482	0.907	54.8 ± 10.6	0.44	(0.08;0.81)	0.019	0.195	0.019	0.199
Tyrosine	Tyr	74.8 ± 24.1	0.12	(-0.05;0.29)	0.180	0.523	71.9 ± 21.3	ref.	74.7 ± 26.0	0.07	(-0.28;0.43)	0.686	0.907	77.6 ± 24.8	0.32	(-0.05;0.69)	0.093	0.338	0.085	0.369
Valine	Val	242.1 ± 64.0	0.13	(-0.04;0.29)	0.137	0.472	243.2 ± 53.3	ref.	231.1 ± 68.5	-0.09	(-0.43;0.26)	0.626	0.907	251.9 ± 68.4	0.36	(0.00;0.71)	0.051	0.273	0.060	0.329
**BIOGENIC AMINES**																				
Creatinine	Creatinine	74.5 ± 17.4	0.03	(-0.10;0.17)	0.630	0.891	76.5 ± 14.8	ref.	77.6 ± 21.2	0.28	(-0.01;0.57)	0.056	0.685	69.3 ± 14.3	0.05	(-0.25;0.35)	0.723	0.866	0.715	0.868
Kynurenine	Kynurenine	2.7 ± 0.7	-0.13	(-0.29;0.03)	0.125	0.471	2.7 ± 0.9	ref.	2.7 ± 0.7	-0.05	(-0.39;0.30)	0.792	0.907	2.5 ± 0.5	-0.14	(-0.50;0.21)	0.435	0.715	0.604	0.839
Sarcosine	Sarcosine	4.4 ± 1.1	-0.18	(-0.35;-0.01)	**0.038**	0.252	4.8 ± 1.2	ref.	4.2 ± 0.9	-0.34	(-0.70;0.01)	0.061	0.685	4.2 ± 1.1	-0.41	(-0.78;-0.04)	**0.033**	0.259	**0.020**	0.199
Serotonin	Serotonin	0.8 ± 0.6	0.01	(-0.16;0.18)	0.942	0.970	0.8 ± 0.6	ref.	0.8 ± 0.6	-0.28	(-0.64;0.08)	0.125	0.728	0.7 ± 0.5	-0.29	(-0.66;0.08)	0.127	0.371	0.163	0.445
Taurine	Taurine	120.9 ± 49.4	0.04	(-0.13;0.21)	0.638	0.891	120.6 ± 48.2	ref.	118.8 ± 52.1	-0.23	(-0.59;0.14)	0.223	0.907	123.2 ± 48.4	-0.19	(-0.57;0.19)	0.330	0.604	0.321	0.604
Trans 4-Hydroxyproline	t4-OH-Pro	10.2 ± 5.0	-0.12	(-0.29;0.05)	0.164	0.517	10.6 ± 4.1	ref.	10.1 ± 4.4	0.05	(-0.31;0.40)	0.789	0.907	9.8 ± 6.3	-0.09	(-0.45;0.28)	0.642	0.835	0.602	0.839
Asymmetric dimethylarginine	ADMA	0.5 ± 0.1	-0.12	(-0.27;0.04)	0.136	0.472	0.5 ± 0.1	ref.	0.5 ± 0.1	-0.02	(-0.34;0.30)	0.908	0.944	0.4 ± 0.1	-0.28	(-0.61;0.06)	0.106	0.339	0.106	0.369
Symmetric dimethylarginine	SDMA	0.5 ± 0.1	-0.05	(-0.21;0.11)	0.554	0.843	0.5 ± 0.1	ref.	0.5 ± 0.1	0.17	(-0.16;0.50)	0.311	0.907	0.4 ± 0.1	-0.28	(-0.62;0.06)	0.112	0.340	0.116	0.369
**GLYCEROPHOSPHOLIPIDS**																				
Lysophosphatidylcholine (acyl) C16:0	lysoPC a C16:0	90.4 ± 20.1	-0.12	(-0.29;0.06)	0.196	0.528	93.2 ± 22.9	ref.	87.3 ± 18.3	-0.20	(-0.57;0.17)	0.297	0.907	90.8 ± 18.7	-0.12	(-0.50;0.26)	0.540	0.786	0.444	0.725
Lysophosphatidylcholine (acyl) C16:1	lysoPC a C16:1	2.6 ± 0.9	-0.28	(-0.45;-0.11)	**0.001**	0.056	2.8 ± 1.2	ref.	2.5 ± 0.8	-0.35	(-0.71;0.01)	0.061	0.685	2.4 ± 0.7	-0.44	(-0.82;-0.06)	**0.024**	0.213	**0.018**	0.199
Lysophosphatidylcholine (acyl) C17:0	lysoPC a C17:0	1.6 ± 0.5	0.11	(-0.06;0.28)	0.198	0.528	1.5 ± 0.6	ref.	1.5 ± 0.5	0.16	(-0.19;0.51)	0.380	0.907	1.7 ± 0.4	0.24	(-0.12;0.61)	0.197	0.480	0.247	0.542
Lysophosphatidylcholine (acyl) C18:0	lysoPC a C18:0	26.4 ± 6.8	-0.04	(-0.21;0.13)	0.643	0.891	26.3 ± 7.9	ref.	25.8 ± 6.4	-0.04	(-0.40;0.32)	0.846	0.919	27 ± 6.1	-0.04	(-0.41;0.34)	0.848	0.936	0.730	0.868
Lysophosphatidylcholine (acyl) C18:1	lysoPC a C18:1	17.9 ± 5.4	-0.19	(-0.36;-0.02)	**0.026**	0.208	18.9 ± 6.6	ref.	16.9 ± 4.3	-0.30	(-0.65;0.06)	0.108	0.728	18.0 ± 4.8	-0.19	(-0.57;0.18)	0.312	0.596	0.233	0.542
Lysophosphatidylcholine (acyl) C18:2	lysoPC a C18:2	29.2 ± 10.4	-0.05	(-0.22;0.12)	0.551	0.843	30.1 ± 11.8	ref.	28.1 ± 10.2	-0.06	(-0.41;0.29)	0.754	0.907	29.6 ± 9.0	0.03	(-0.33;0.39)	0.868	0.936	0.939	0.968
Lysophosphatidylcholine (acyl) C20:3	lysoPC a C20:3	2.0 ± 0.7	-0.16	(-0.33;0.01)	0.059	0.361	2.2 ± 0.9	ref.	1.9 ± 0.5	-0.28	(-0.64;0.07)	0.120	0.728	2.0 ± 0.6	-0.25	(-0.62;0.12)	0.194	0.480	0.148	0.430
Lysophosphatidylcholine (acyl) C20:4	lysoPC a C20:4	5.9 ± 1.7	-0.12	(-0.29;0.05)	0.158	0.517	6.2 ± 2.1	ref.	5.7 ± 1.6	-0.11	(-0.48;0.25)	0.536	0.907	5.8 ± 1.4	-0.16	(-0.54;0.22)	0.407	0.686	0.317	0.604
Lysophosphatidylcholine (acyl) C24:0	lysoPC a C24:0	0.14 ± 0.03	-0.01	(-0.17;0.14)	0.867	0.947	0.13 ± 0.03	ref.	0.13 ± 0.03	-0.08	(-0.41;0.24)	0.618	0.907	0.14 ± 0.03	0.13	(-0.21;0.47)	0.461	0.718	0.539	0.776
Lysophosphatidylcholine (acyl) C28:1	lysoPC a C28:1	0.3 ± 0.1	0.07	(-0.07;0.21)	0.359	0.707	0.3 ± 0.1	ref.	0.3 ± 0.1	0.12	(-0.17;0.42)	0.419	0.907	0.3 ± 0.1	0.18	(-0.13;0.49)	0.248	0.522	0.254	0.542
Phosphatidylcholine (diacyl) C28:1	PC aa C28:1	3.0 ± 0.8	0.10	(-0.06;0.26)	0.212	0.531	2.8 ± 0.7	ref.	3 ± 0.8	0.17	(-0.17;0.50)	0.327	0.907	3.2 ± 0.7	0.31	(-0.04;0.65)	0.085	0.327	0.098	0.369
Phosphatidylcholine (diacyl) C30:0	PC aa C30:0	4.0 ± 1.3	-0.04	(-0.21;0.13)	0.634	0.891	4.0 ± 1.5	ref.	3.9 ± 1.2	-0.09	(-0.46;0.27)	0.617	0.907	4.2 ± 1.3	-0.02	(-0.39;0.36)	0.932	0.968	0.961	0.976
Phosphatidylcholine (diacyl) C32:0	PC aa C32:0	26.6 ± 5.5	-0.08	(-0.24;0.09)	0.364	0.707	26.5 ± 6.3	ref.	26 ± 4.9	-0.17	(-0.52;0.18)	0.351	0.907	27.4 ± 5.1	-0.02	(-0.39;0.34)	0.898	0.947	0.898	0.960
Phosphatidylcholine (diacyl) C32:1	PC aa C32:1	32.0 ± 16.2	-0.21	(-0.39;-0.04)	**0.015**	0.153	35.2 ± 20.9	ref.	30.5 ± 13.7	-0.29	(-0.65;0.08)	0.123	0.728	30.3 ± 12.4	-0.33	(-0.71;0.04)	0.083	0.327	0.084	0.369

			Continuous				Tertile 1		Tertile 2					Tertile 3						
**Metabolite**	**Abbreviation**	Mean ± SD [2]	β	95% CI [3]	*p* -value	p_{FDR} [4]	Mean ± SD [2]	β	Mean ± SD [2]	β	95% CI [3]	*p* -value	p_{FDR} [4]	Mean ± SD [2]	β	95% CI [3]	*p* -value	p_{FDR} [4]	p_{trend}	$p_{trend\ FDR}$ [4]
Phosphatidylcholine (diacyl) C32:2	PC aa C32:2	8.3 ± 3.1	0.02	(-0.15;0.19)	0.785	0.947	7.9 ± 3.2	ref.	8.6 ± 3.1	0.22	(-0.13;0.58)	0.223	0.907	8.6 ± 3.0	0.07	(-0.30;0.45)	0.694	0.853	0.732	0.868
Phosphatidylcholine (diacyl) C32:3	PC aa C32:3	0.9 ± 0.2	0.04	(-0.12;0.19)	0.646	0.891	0.8 ± 0.3	ref.	0.9 ± 0.2	0.17	(-0.16;0.50)	0.308	0.907	1.0 ± 0.2	0.17	(-0.17;0.51)	0.330	0.604	0.403	0.683
Phosphatidylcholine (diacyl) C34:1	PC aa C34:1	365.4 ± 82.8	-0.21	(-0.38;-0.04)	**0.017**	0.164	377.4 ± 96	ref.	350.7 ± 76.4	-0.36	(-0.72;0.00)	0.051	0.685	368.1 ± 73.2	-0.27	(-0.64;0.10)	0.155	0.424	0.143	0.425
Phosphatidylcholine (diacyl) C34:2	PC aa C34:2	739.8 ± 128.1	0.00	(-0.18;0.17)	0.980	0.983	723.8 ± 137	ref.	739.4 ± 127.2	0.07	(-0.29;0.44)	0.702	0.907	756.1 ± 119.3	0.10	(-0.28;0.48)	0.616	0.818	0.668	0.845
Phosphatidylcholine (diacyl) C34:3	PC aa C34:3	30.1 ± 10.2	-0.11	(-0.28;0.06)	0.202	0.528	29.6 ± 10.2	ref.	29.7 ± 9.4	-0.05	(-0.41;0.30)	0.778	0.907	31.1 ± 10.9	-0.11	(-0.48;0.26)	0.569	0.804	0.493	0.754
Phosphatidylcholine (diacyl) C34:4	PC aa C34:4	3.1 ± 1.2	0.01	(-0.16;0.18)	0.904	0.947	3.0 ± 1.3	ref.	3.1 ± 1.1	0.18	(-0.17;0.54)	0.314	0.907	3.2 ± 1.1	0.07	(-0.30;0.44)	0.704	0.858	0.769	0.880
Phosphatidylcholine (diacyl) C36:0	PC aa C36:0	3.2 ± 1.0	0.10	(-0.07;0.26)	0.243	0.593	3.1 ± 1.2	ref.	3.2 ± 0.9	0.18	(-0.17;0.53)	0.312	0.907	3.4 ± 1.0	0.30	(-0.07;0.66)	0.110	0.340	0.135	0.411
Phosphatidylcholine (diacyl) C36:1	PC aa C36:1	62.9 ± 17.8	-0.13	(-0.29;0.04)	0.130	0.471	63.3 ± 19.9	ref.	60.20 ± 15.3	-0.20	(-0.54;0.14)	0.252	0.907	65.3 ± 17.7	-0.14	(-0.49;0.22)	0.454	0.716	0.431	0.713
Phosphatidylcholine (diacyl) C36:2	PC aa C36:2	384.7 ± 79.7	0.02	(-0.15;0.18)	0.836	0.947	369.3 ± 79.1	ref.	386.6 ± 82.6	0.13	(-0.21;0.48)	0.444	0.907	398.3 ± 75.7	0.08	(-0.28;0.44)	0.665	0.849	0.719	0.868
Phosphatidylcholine (diacyl) C36:3	PC aa C36:3	192.6 ± 43.8	-0.08	(-0.25;0.08)	0.321	0.682	191.9 ± 49.8	ref.	188.7 ± 38.9	-0.08	(-0.43;0.26)	0.636	0.907	197.2 ± 42.2	-0.11	(-0.47;0.25)	0.538	0.786	0.501	0.754
Phosphatidylcholine (diacyl) C36:4	PC aa C36:4	291.5 ± 72.0	-0.03	(-0.19;0.14)	0.742	0.947	289.8 ± 76.5	ref.	290.20 ± 71.2	0.04	(-0.31;0.39)	0.810	0.919	294.4 ± 69.2	-0.03	(-0.39;0.33)	0.871	0.936	0.804	0.912
Phosphatidylcholine (diacyl) C36:5	PC aa C36:5	45.8 ± 27.8	0.03	(-0.14;0.20)	0.744	0.947	46.8 ± 36.4	ref.	42.2 ± 20.4	-0.02	(-0.38;0.34)	0.930	0.944	48.2 ± 24	0.22	(-0.15;0.59)	0.249	0.522	0.275	0.562
Phosphatidylcholine (diacyl) C36:6	PC aa C36:6	1.5 ± 0.6	0.13	(-0.04;0.30)	0.129	0.471	1.4 ± 0.7	ref.	1.5 ± 0.6	0.21	(-0.14;0.56)	0.243	0.907	1.7 ± 0.7	0.37	(0.01;0.74)	**0.046**	0.263	0.064	0.329
Phosphatidylcholine (diacyl) C38:0	PC aa C38:0	2.9 ± 0.9	0.08	(-0.08;0.25)	0.312	0.675	2.7 ± 1.0	ref.	2.8 ± 0.7	0.15	(-0.19;0.49)	0.389	0.907	3.1 ± 0.8	0.32	(-0.04;0.67)	0.079	0.327	0.089	0.369
Phosphatidylcholine (diacyl) C38:3	PC aa C38:3	53.2 ± 13.6	-0.11	(-0.27;0.04)	0.158	0.517	52.9 ± 14.1	ref.	52.1 ± 12.5	-0.16	(-0.49;0.16)	0.330	0.907	54.6 ± 14.3	-0.22	(-0.56;0.12)	0.198	0.480	0.194	0.490
Phosphatidylcholine (diacyl) C38:4	PC aa C38:4	131.5 ± 35.0	-0.05	(-0.20;0.11)	0.562	0.845	130 ± 39.0	ref.	132.1 ± 33	0.06	(-0.27;0.38)	0.738	0.907	132.5 ± 33.1	-0.12	(-0.46;0.22)	0.479	0.737	0.456	0.737
Phosphatidylcholine (diacyl) C38:5	PC aa C38:5	66.2 ± 19.0	-0.07	(-0.23;0.09)	0.401	0.729	66.4 ± 21.8	ref.	63.9 ± 16.5	-0.12	(-0.46;0.22)	0.500	0.907	68.4 ± 18.5	-0.05	(-0.40;0.31)	0.798	0.930	0.738	0.868
Phosphatidylcholine (diacyl) C38:6	PC aa C38:6	101.1 ± 33.6	0.13	(-0.03;0.30)	0.124	0.471	95.7 ± 36.4	ref.	97.4 ± 27.3	0.12	(-0.23;0.47)	0.508	0.907	110.20 ± 35	0.38	(0.02;0.74)	**0.039**	0.263	0.053	0.329
Phosphatidylcholine (diacyl) C40:2	PC aa C40:2	0.3 ± 0.1	-0.08	(-0.24;0.08)	0.346	0.707	0.3 ± 0.1	ref.	0.3 ± 0.1	-0.15	(-0.50;0.19)	0.381	0.907	0.3 ± 0.1	0.03	(-0.33;0.39)	0.875	0.936	0.939	0.968
Phosphatidylcholine (diacyl) C40:3	PC aa C40:3	0.4 ± 0.1	-0.13	(-0.29;0.03)	0.110	0.471	0.4 ± 0.2	ref.	0.3 ± 0.1	-0.36	(-0.70;-0.03)	**0.034**	0.685	0.4 ± 0.1	-0.16	(-0.50;0.19)	0.378	0.649	0.329	0.604
Phosphatidylcholine (diacyl) C40:4	PC aa C40:4	3.0 ± 0.8	-0.22	(-0.39;-0.06)	**0.010**	0.113	3.2 ± 1.1	ref.	2.9 ± 0.6	-0.39	(-0.74;-0.04)	**0.031**	0.685	2.9 ± 0.7	-0.54	(-0.90;-0.18)	**0.004**	0.091	**0.004**	0.098
Phosphatidylcholine (diacyl) C40:5	PC aa C40:5	8.3 ± 2.4	-0.19	(-0.35;-0.02)	**0.025**	0.208	8.6 ± 2.9	ref.	8.1 ± 2.1	-0.27	(-0.62;0.08)	0.130	0.728	8.1 ± 2.3	-0.42	(-0.78;-0.06)	**0.023**	0.213	**0.024**	0.203
Phosphatidylcholine (diacyl) C40:6	PC aa C40:6	28.3 ± 10.2	0.07	(-0.09;0.23)	0.403	0.729	27.1 ± 11.6	ref.	27.5 ± 7.8	0.07	(-0.28;0.41)	0.694	0.907	30.3 ± 10.7	0.22	(-0.14;0.58)	0.233	0.512	0.246	0.542
Phosphatidylcholine (diacyl) C42:0	PC aa C42:0	0.3 ± 0.1	0.06	(-0.10;0.22)	0.463	0.774	0.3 ± 0.1	ref.	0.3 ± 0.1	0.13	(-0.21;0.46)	0.450	0.907	0.4 ± 0.1	0.29	(-0.06;0.64)	0.102	0.338	0.116	0.369
Phosphatidylcholine (diacyl) C42:1	PC aa C42:1	0.18 ± 0.05	0.09	(-0.07;0.24)	0.291	0.651	0.17 ± 0.05	ref.	0.17 ± 0.04	0.11	(-0.22;0.45)	0.509	0.907	0.2 ± 0.1	0.31	(-0.03;0.66)	0.077	0.327	0.089	0.369
Phosphatidylcholine (diacyl) C42:2	PC aa C42:2	0.16 ± 0.04	0.02	(-0.14;0.19)	0.790	0.947	0.15 ± 0.05	ref.	0.15 ± 0.04	0.12	(-0.22;0.47)	0.482	0.907	0.16 ± 0.03	0.25	(-0.11;0.61)	0.169	0.443	0.189	0.487
Phosphatidylcholine (diacyl) C42:4	PC aa C42:4	0.10 ± 0.02	-0.09	(-0.26;0.08)	0.291	0.651	0.10 ± 0.03	ref.	0.11 ± 0.03	-0.04	(-0.40;0.32)	0.826	0.919	0.10 ± 0.02	-0.26	(-0.64;0.11)	0.175	0.450	0.182	0.479
Phosphatidylcholine (diacyl) C42:5	PC aa C42:5	0.3 ± 0.1	-0.10	(-0.27;0.07)	0.266	0.615	0.3 ± 0.2	ref.	0.2 ± 0.1	-0.26	(-0.62;0.10)	0.159	0.812	0.3 ± 0.1	-0.14	(-0.52;0.23)	0.448	0.715	0.464	0.740
Phosphatidylcholine (diacyl) C42:6	PC aa C42:6	0.4 ± 0.1	-0.15	(-0.32;0.01)	0.071	0.388	0.4 ± 0.2	ref.	0.3 ± 0.1	-0.40	(-0.76;-0.05)	**0.025**	0.685	0.4 ± 0.1	-0.30	(-0.67;0.06)	0.103	0.338	0.096	0.369
Phosphatidylcholine (acyl-alkyl) C30:0	PC ae C30:0	0.3 ± 0.1	0.02	(-0.15;0.18)	0.825	0.947	0.3 ± 0.1	ref.	0.3 ± 0.1	0.02	(-0.33;0.37)	0.911	0.944	0.4 ± 0.1	0.09	(-0.27;0.45)	0.640	0.835	0.652	0.845
Phosphatidylcholine (acyl-alkyl) C30:2	PC ae C30:2	0.08 ± 0.02	0.20	(0.06;0.34)	**0.006**	0.098	0.07 ± 0.02	ref.	0.09 ± 0.02	0.42	(0.13;0.72)	**0.006**	0.685	0.09 ± 0.02	0.50	(0.2;0.81)	**0.002**	0.091	**0.002**	0.098
Phosphatidylcholine (acyl-alkyl) C32:1	PC ae C32:1	5.3 ± 1.2	-0.02	(-0.18;0.15)	0.841	0.947	5.0 ± 1.2	ref.	5.3 ± 1.3	0.09	(-0.26;0.44)	0.613	0.907	5.4 ± 1.1	0.10	(-0.26;0.46)	0.586	0.804	0.613	0.839
Phosphatidylcholine (acyl-alkyl) C32:2	PC ae C32:2	1.4 ± 0.4	0.01	(-0.14;0.17)	0.873	0.947	1.3 ± 0.4	ref.	1.4 ± 0.4	0.17	(-0.16;0.50)	0.321	0.907	1.4 ± 0.3	0.19	(-0.16;0.53)	0.294	0.571	0.321	0.604
Phosphatidylcholine (acyl-alkyl) C34:0	PC ae C34:0	2.5 ± 0.6	0.06	(-0.11;0.22)	0.484	0.782	2.3 ± 0.7	ref.	2.4 ± 0.6	0.08	(-0.26;0.43)	0.641	0.907	2.6 ± 0.6	0.20	(-0.16;0.56)	0.272	0.537	0.247	0.542
Phosphatidylcholine (acyl-alkyl) C34:1	PC ae C34:1	18.0 ± 3.9	-0.06	(-0.22;0.10)	0.471	0.774	17.4 ± 4.1	ref.	18.0 ± 4.0	-0.05	(-0.38;0.29)	0.788	0.907	18.8 ± 3.4	0.01	(-0.34;0.35)	0.968	0.968	0.955	0.976
Phosphatidylcholine (acyl-alkyl) C34:2	PC ae C34:2	20.2 ± 5.3	0.01	(-0.15;0.17)	0.926	0.962	19.3 ± 5.7	ref.	20.5 ± 5.4	0.18	(-0.16;0.52)	0.307	0.907	20.9 ± 4.8	0.08	(-0.27;0.44)	0.650	0.838	0.713	0.868
Phosphatidylcholine (acyl-alkyl) C34:3	PC ae C34:3	13.1 ± 4.0	0.05	(-0.12;0.21)	0.570	0.849	12.4 ± 3.7	ref.	13.1 ± 4.2	0.13	(-0.21;0.47)	0.461	0.907	13.9 ± 3.8	0.23	(-0.12;0.59)	0.204	0.480	0.282	0.562
Phosphatidylcholine (acyl-alkyl) C36:0	PC ae C36:0	1.2 ± 0.3	-0.15	(-0.32;0.02)	0.082	0.392	1.2 ± 0.4	ref.	1.1 ± 0.2	-0.31	(-0.67;0.04)	0.088	0.728	1.2 ± 0.2	-0.19	(-0.56;0.18)	0.322	0.604	0.339	0.604
Phosphatidylcholine (acyl-alkyl) C36:1	PC ae C36:1	11.5 ± 2.7	0.07	(-0.08;0.23)	0.356	0.707	10.8 ± 2.7	ref.	11.4 ± 2.7	0.13	(-0.21;0.46)	0.452	0.907	12.2 ± 2.5	0.21	(-0.13;0.56)	0.226	0.511	0.230	0.542
Phosphatidylcholine (acyl-alkyl) C36:2	PC ae C36:2	21.7 ± 5.4	0.16	(0.01;0.32)	**0.036**	0.252	19.9 ± 5.5	ref.	22.0 ± 5.4	0.31	(-0.01;0.64)	0.057	0.685	23.3 ± 4.8	0.35	(0.01;0.68)	**0.043**	0.263	0.052	0.329
Phosphatidylcholine (acyl-alkyl) C36:3	PC ae C36:3	11.4 ± 2.8	-0.06	(-0.22;0.10)	0.463	0.774	11.1 ± 3	ref.	11.4 ± 2.9	0.05	(-0.29;0.39)	0.782	0.907	11.7 ± 2.6	-0.04	(-0.39;0.32)	0.841	0.936	0.759	0.880
Phosphatidylcholine (acyl-alkyl) C36:4	PC ae C36:4	26.3 ± 6.4	-0.07	(-0.24;0.10)	0.412	0.736	26.2 ± 6.9	ref.	26.1 ± 5.4	0.02	(-0.34;0.38)	0.918	0.944	26.4 ± 6.9	-0.10	(-0.47;0.27)	0.597	0.804	0.532	0.775
Phosphatidylcholine (acyl-alkyl) C36:5	PC ae C36:5	18.3 ± 5.0	0.01	(-0.15;0.17)	0.899	0.947	17.8 ± 5.4	ref.	18.4 ± 4.4	0.20	(-0.14;0.55)	0.246	0.907	18.8 ± 5.1	0.14	(-0.22;0.50)	0.447	0.715	0.524	0.775
Phosphatidylcholine (acyl-alkyl) C38:0	PC ae C38:0	2.5 ± 0.8	0.11	(-0.06;0.27)	0.205	0.528	2.4 ± 0.9	ref.	2.4 ± 0.7	0.12	(-0.22;0.47)	0.483	0.907	2.7 ± 0.8	0.36	(0.01;0.72)	**0.047**	0.263	0.074	0.354
Phosphatidylcholine (acyl-alkyl) C38:2	PC ae C38:2	2.2 ± 0.6	0.01	(-0.15;0.17)	0.890	0.947	2.1 ± 0.6	ref.	2.2 ± 0.6	0.14	(-0.20;0.48)	0.435	0.907	2.3 ± 0.5	0.07	(-0.28;0.43)	0.686	0.851	0.716	0.868
Phosphatidylcholine (acyl-alkyl) C38:3	PC ae C38:3	4.8 ± 1.1	0.06	(-0.09;0.22)	0.423	0.746	4.5 ± 1.1	ref.	4.8 ± 1.1	0.13	(-0.20;0.45)	0.442	0.907	5.0 ± 1.1	0.16	(-0.18;0.50)	0.359	0.625	0.361	0.629
Phosphatidylcholine (acyl-alkyl) C38:4	PC ae C38:4	17.2 ± 3.6	0.00	(-0.16;0.16)	0.983	0.983	16.5 ± 3.5	ref.	17.4 ± 3.5	0.14	(-0.20;0.49)	0.410	0.907	17.7 ± 3.9	0.01	(-0.34;0.37)	0.943	0.968	0.997	0.997
Phosphatidylcholine (acyl-alkyl) C38:5	PC ae C38:5	21.9 ± 4.7	-0.09	(-0.25;0.07)	0.263	0.615	21.7 ± 5.0	ref.	21.8 ± 4.1	0.03	(-0.31;0.38)	0.850	0.919	22.3 ± 4.9	-0.08	(-0.43;0.28)	0.678	0.849	0.611	0.839
Phosphatidylcholine (acyl-alkyl) C38:6	PC ae C38:6	9.2 ± 2.6	0.08	(-0.08;0.24)	0.352	0.707	8.7 ± 3.0	ref.	9.2 ± 2.2	0.24	(-0.10;0.58)	0.164	0.812	9.8 ± 2.5	0.30	(-0.05;0.65)	0.092	0.338	0.110	0.369
Phosphatidylcholine (acyl-alkyl) C40:1	PC ae C40:1	1.1 ± 0.3	0.03	(-0.13;0.20)	0.700	0.919	1.1 ± 0.3	ref.	1.1 ± 0.3	0.10	(-0.26;0.45)	0.592	0.907	1.2 ± 0.3	0.23	(-0.14;0.59)	0.224	0.511	0.285	0.562
Phosphatidylcholine (acyl-alkyl) C40:2	PC ae C40:2	1.6 ± 0.4	0.14	(-0.02;0.30)	0.078	0.388	1.5 ± 0.4	ref.	1.5 ± 0.4	0.09	(-0.25;0.42)	0.610	0.907	1.7 ± 0.4	0.39	(0.04;0.74)	**0.029**	0.240	**0.031**	0.243
Phosphatidylcholine (acyl-alkyl) C40:3	PC ae C40:3	0.8 ± 0.2	-0.01	(-0.17;0.14)	0.860	0.947	0.8 ± 0.2	ref.	0.8 ± 0.2	-0.04	(-0.36;0.28)	0.791	0.907	0.9 ± 0.2	0.09	(-0.24;0.42)	0.595	0.804	0.668	0.845
Phosphatidylcholine (acyl-alkyl) C40:4	PC ae C40:4	2.2 ± 0.4	-0.02	(-0.18;0.14)	0.767	0.947	2.1 ± 0.4	ref.	2.2 ± 0.4	-0.05	(-0.39;0.29)	0.762	0.907	2.3 ± 0.5	-0.01	(-0.36;0.34)	0.953	0.968	0.927	0.968
Phosphatidylcholine (acyl-alkyl) C40:5	PC ae C40:5	3.4 ± 0.7	-0.01	(-0.17;0.15)	0.900	0.947	3.2 ± 0.6	ref.	3.4 ± 0.7	0.05	(-0.29;0.38)	0.789	0.907	3.5 ± 0.7	0.10	(-0.25;0.44)	0.591	0.804	0.610	0.839
Phosphatidylcholine (acyl-alkyl) C40:6	PC ae C40:6	4.8 ± 1.2	0.13	(-0.03;0.29)	0.102	0.471	4.4 ± 1.3	ref.	4.7 ± 1.1	0.26	(-0.07;0.59)	0.129	0.728	5.2 ± 1.3	0.42	(0.07;0.76)	**0.019**	0.195	**0.022**	0.199
Phosphatidylcholine (acyl-alkyl) C42:1	PC ae C42:1	0.3 ± 0.1	-0.15	(-0.30;0.01)	0.075	0.388	0.3 ± 0.1	ref.	0.3 ± 0.1	-0.39	(-0.72;-0.05)	**0.024**	0.685	0.3 ± 0.1	-0.26	(-0.60;0.09)	0.149	0.417	0.100	0.369
Phosphatidylcholine (acyl-alkyl) C42:2	PC ae C42:2	0.4 ± 0.1	0.02	(-0.13;0.18)	0.759	0.947	0.4 ± 0.1	ref.	0.4 ± 0.1	-0.14	(-0.46;0.19)	0.408	0.907	0.5 ± 0.1	0.19	(-0.15;0.53)	0.271	0.537	0.327	0.604
Phosphatidylcholine (acyl-alkyl) C42:3	PC ae C42:3	0.6 ± 0.1	0.04	(-0.12;0.21)	0.5847	0.861	0.5 ± 0.1	ref.	0.5 ± 0.1	-0.07	(-0.40;0.27)	0.6975	0.907	0.6 ± 0.2	0.27	(-0.07;0.62)	0.1238	0.369	0.182	0.479
Phosphatidylcholine (acyl-alkyl) C42:4	PC ae C42:4	0.7 ± 0.2	-0.04	(-0.20;0.12)	0.652	0.891	0.7 ± 0.1	ref.	0.7 ± 0.2	-0.10	(-0.44;0.25)	0.582	0.907	0.7 ± 0.2	0.01	(-0.35;0.36)	0.964	0.968	0.994	0.997
Phosphatidylcholine (acyl-alkyl) C42:5	PC ae C42:5	1.5 ± 0.3	-0.02	(-0.19;0.14)	0.797	0.947	1.5 ± 0.3	ref.	1.5 ± 0.3	0.04	(-0.31;0.38)	0.841	0.919	1.6 ± 0.4	0.10	(-0.26;0.45)	0.600	0.804	0.660	0.845
Phosphatidylcholine (acyl-alkyl) C44:3	PC ae C44:3	0.07 ± 0.02	-0.11	(-0.27;0.05)	0.170	0.517	0.07 ± 0.02	ref.	0.07 ± 0.02	-0.21	(-0.54;0.12)	0.217	0.907	0.07 ± 0.02	-0.17	(-0.52;0.18)	0.334	0.604	0.343	0.604
Phosphatidylcholine (acyl-alkyl) C44:4	PC ae C44:4	0.2 ± 0.1	0.01	(-0.15;0.17)	0.899	0.947	0.2 ± 0.1	ref.	0.2 ± 0.1	-0.06	(-0.40;0.29)	0.743	0.907	0.2 ± 0.1	0.17	(-0.19;0.52)	0.359	0.625	0.416	0.697
Phosphatidylcholine (acyl-alkyl) C44:5	PC ae C44:5	1.0 ± 0.3	-0.06	(-0.23;0.10)	0.456	0.774	1.0 ± 0.3	ref.	1.0 ± 0.3	-0.10	(-0.45;0.26)	0.597	0.907	1.0 ± 0.3	-0.05	(-0.41;0.32)	0.810	0.936	0.766	0.880
Phosphatidylcholine (acyl-alkyl) C44:6	PC ae C44:6	0.8 ± 0.2	-0.02	(-0.18;0.14)	0.811	0.947	0.8 ± 0.2	ref.	0.8 ± 0.2	0.03	(-0.31;0.37)	0.848	0.919	0.8 ± 0.2	0.10	(-0.25;0.45)	0.589	0.804	0.665	0.845
**SPHINGOLIPIDS**																				
Hydroxysphingomyelin C14:1	SM OH C14:1	4.0 ± 1.0	0.20	(0.06;0.35)	**0.007**	0.098	3.5 ± 0.9	ref.	4.0 ± 1.1	0.27	(-0.03;0.58)	0.076	0.728	4.4 ± 1.0	0.50	(0.18;0.81)	**0.002**	0.091	**0.002**	0.098

			Continuous				Tertile 1		Tertile 2					Tertile 3						
**Metabolite**	**Abbreviation**	Mean ± SD [2]	β	95% CI [3]	*p*-value	p_{FDR} [4]	Mean ± SD [2]	β	Mean ± SD [2]	β	95% CI [3]	*p*-value	p_{FDR} [4]	Mean ± SD [2]	β	95% CI [3]	*p*-value	p_{FDR} [4]	p_{trend}	$p_{trend\ FDR}$ [4]
Hydroxysphingomyelin C16:1	SM OH C16:1	1.9 ± 0.5	0.22	(0.08;0.37)	**0.004**	0.095	1.6 ± 0.4	ref.	1.8 ± 0.5	0.26	(-0.06;0.57)	0.110	0.728	2.1 ± 0.5	0.51	(0.18;0.83)	**0.003**	0.091	**0.002**	0.098
Hydroxysphingomyelin C22:1	SM OH C22:1	4.4 ± 1.1	0.20	(0.05;0.35)	**0.010**	0.113	4.0 ± 1.1	ref.	4.4 ± 1.0	0.29	(-0.03;0.61)	0.081	0.728	4.8 ± 1.0	0.43	(0.1;0.76)	**0.013**	0.168	**0.014**	0.188
Hydroxysphingomyelin C22:2	SM OH C22:2	3.3 ± 0.9	0.20	(0.06;0.34)	**0.007**	0.098	2.9 ± 0.8	ref.	3.3 ± 0.9	0.30	(0.00;0.60)	0.053	0.685	3.7 ± 0.8	0.46	(0.15;0.77)	**0.004**	0.091	**0.006**	0.118
Hydroxysphingomyelin C24:1	SM OH C24:1	0.3 ± 0.1	0.19	(0.03;0.35)	**0.025**	0.208	0.3 ± 0.1	ref.	0.3 ± 0.1	0.14	(-0.21;0.48)	0.439	0.907	0.3 ± 0.1	0.36	(0.01;0.72)	**0.047**	0.263	0.061	0.329
Sphingomyelin C16:0	SM C16:0	67.2 ± 13.6	-0.02	(-0.17;0.13)	0.790	0.947	65.5 ± 14.8	ref.	65.3 ± 13.8	-0.16	(-0.48;0.16)	0.324	0.907	70.6 ± 11.6	0.06	(-0.27;0.39)	0.715	0.863	0.846	0.922
Sphingomyelin C16:1	SM C16:1	9.5 ± 2.2	0.06	(-0.08;0.21)	0.386	0.720	8.8 ± 2.0	ref.	9.4 ± 2.5	0.10	(-0.21;0.40)	0.536	0.907	10.1 ± 1.9	0.23	(-0.08;0.55)	0.144	0.411	0.226	0.542
Sphingomyelin C18:0	SM C18:0	12.0 ± 3.3	0.06	(-0.10;0.22)	0.474	0.774	11.6 ± 3.8	ref.	11.6 ± 3.1	-0.08	(-0.41;0.25)	0.640	0.907	12.9 ± 2.7	0.20	(-0.14;0.55)	0.253	0.522	0.281	0.562
Sphingomyelin C18:1	SM C18:1	5.7 ± 1.6	0.10	(-0.05;0.25)	0.189	0.528	5.2 ± 1.6	ref.	5.6 ± 1.8	0.07	(-0.24;0.38)	0.645	0.907	6.2 ± 1.4	0.30	(-0.03;0.62)	0.075	0.327	0.100	0.369
Sphingomyelin C20:2	SM C20:2	0.2 ± 0.1	0.08	(-0.07;0.23)	0.298	0.654	0.14 ± 0.05	ref.	0.15 ± 0.05	0.17	(-0.15;0.49)	0.304	0.907	0.2 ± 0.1	0.13	(-0.20;0.47)	0.445	0.715	0.477	0.751
Sphingomyelin C24:0	SM C24:0	6.1 ± 1.4	0.02	(-0.14;0.19)	0.771	0.947	6.0 ± 1.5	ref.	6.0 ± 1.4	-0.04	(-0.38;0.30)	0.826	0.919	6.4 ± 1.3	0.11	(-0.25;0.46)	0.558	0.804	0.662	0.845
Sphingomyelin C24:1	SM C24:1	13.9 ± 3.4	-0.05	(-0.21;0.11)	0.554	0.843	14 ± 4.4	ref.	13.3 ± 3.1	-0.19	(-0.53;0.15)	0.273	0.907	14.4 ± 2.5	0.01	(-0.34;0.36)	0.955	0.968	0.899	0.960
Sphingomyelin C26:0	SM C26:0	0.04 ± 0.01	0.15	(-0.02;0.32)	0.076	0.388	0.03 ± 0.01	ref.	0.04 ± 0.01	0.00	(-0.35;0.35)	0.996	0.998	0.04 ± 0.01	0.31	(-0.06;0.67)	0.101	0.338	0.155	0.433
Sphingomyelin C26:1	SM C26:1	0.10 ± 0.04	0.08	(-0.09;0.25)	0.369	0.707	0.1 ± 0.1	ref.	0.10 ± 0.03	0.15	(-0.21;0.50)	0.420	0.907	0.11 ± 0.03	0.33	(-0.04;0.70)	0.083	0.327	0.102	0.369
**HEXOSES**																				
Hexoses	H1	5574.0 ± 1481.3	-0.01	(-0.18;0.16)	0.897	0.947	5575.9 ± 1303.6	ref.	5451.5 ± 1480.2	-0.11	(-0.47;0.25)	0.543	0.907	5694.4 ± 1652.4	0.04	(-0.34;0.41)	0.845	0.936	0.841	0.922

Bold metabolites and corresponding p-values are significantly associated with colorectal cancer stage after FDR correction;

[1] Tested using multiple linear regression models analyzing associations of dietary exposures (continuous and in tertiles) as independent variable and log transformed Z-standardized metabolite concentrations as dependent variable. The continuous analysis is presented per one-point, ten-points, SD increase for the WCRF dietary score, DHD15-index, and dietary patterns, respectively. Tertile cut-off s the DHD15-index. Tertile cut-off scores were -0.3 and 0.5, -0.1 and 0.4, and -0.3 and 0.5 for the Western, Carnivore, and Prudent pattern, respectively. Regression models were adjusted for sex, age, analytical batch, body mass index (continuous), smoking status, and stage;

[2] untransformed and unadjusted metabolite concentrations in µmol/l;

[3] Confidence Interval;

[4] p-value corrected for False Discovery Rate.
