# Supplementary material for: Diet quality indices and dietary patterns are associated with plasma metabolites in colorectal cancer patients
Source: Eur J Nutr. 2021 Feb 5;60(6):3171–84. doi: 10.1007/s00394-021-02488-1 (PMC8354955; doi:10.1007/s00394-021-02488-1)
Supplement: Supplementary file 3 — Supplementary file3 (DOCX 20 KB) [file 394_2021_2488_MOESM3_ESM.docx]

**Supplementary Table S3** Baseline characteristics by diet quality index and dietary pattern, comparing the lowest tertile (T1) with the highest tertile (T3)

|  |  | ***WCRF dietary score*** | |  | ***DHD15-index*** | |  | ***Western pattern*** | |  | ***Carnivore pattern*** | |  | ***Prudent pattern*** | |
| --- | --- | --- | --- | --- | --- | --- | --- | --- | --- | --- | --- | --- | --- | --- | --- |
|  |  | *T1* | *T3* |  | *T1* | *T3* |  | *T1* | *T3* |  | *T1* | *T3* |  | *T1* | *T3* |
| Number of participants |  | 77 | 62 |  | 65 | 65 |  | 65 | 65 |  | 65 | 65 |  | 65 | 65 |
| Male sex, n (%) |  | 51 (66) | 32 (52) |  | 47 (72) | 36 (55) |  | 35 (54) | 43 (66) |  | 31 (48) | 45 (69) |  | 52 (80) | 26 (40) |
| Age |  | 65.5 ± 8.3 | 66.7 ± 10.1 |  | 64.0 ± 9.5 | 67.4 ± 9.5 |  | 69.5 ± 9.2 | 62.1 ± 8.4 |  | 65.4 ± 10.2 | 67.2 ± 8.2 |  | 65.1 ± 8.0 | 66.2 ± 9.0 |
| Body mass index (kg/m^2^) |  | 25.0 ± 5.6 | 26.0 ± 5.2 |  | 26.4 ± 3.8 | 25.0 ± 5.7 |  | 26.2 ± 3.6 | 25.4 ± 6.0 |  | 23.7 ± 6.2 | 27.0 ± 4.0 |  | 26.6 ± 4.0 | 24.5 ± 5.7 |
| Underweight, <18.5, n (%) |  | 2 (3) | 1 (2) |  | 0 (0) | 2 (3) |  | 0 (0) | 2 (3) |  | 3 (5) | 0 (0) |  | 0 (0) | 2 (3) |
| Normal weight, 18.5-24.9, n (%) |  | 36 (47) | 26 (42) |  | 27 (42) | 27 (42) |  | 28 (43) | 26 (40) |  | 34 (52) | 26 (40) |  | 26 (40) | 31 (48) |
| Overweight, 25-29.9, n (%) |  | 27 (35) | 24 (39) |  | 27 (42) | 29 (45) |  | 26 (40) | 27 (42) |  | 23 (35) | 26 (40) |  | 25 (39) | 26 (40) |
| Obese, ≥30, n (%) |  | 12 (16) | 11 (18) |  | 11 (17) | 7 (11) |  | 11 (17) | 10 (15) |  | 5 (8) | 13 (20) |  | 14 (22) | 6 (9) |
| Smoking, n (%) |  |  |  |  |  |  |  |  |  |  |  |  |  |  |  |
| Current |  | 11 (14) | 7 (11) |  | 15 (23) | 1 (2) |  | 6 (9) | 8 (12) |  | 9 (14) | 6 (9) |  | 12 (19) | 2 (3) |
| Former |  | 49 (64) | 37 (60) |  | 36 (55) | 42 (65) |  | 34 (52) | 40 (62) |  | 31 (48) | 44 (68) |  | 39 (60) | 41 (63) |
| Never |  | 17 (22) | 18 (29) |  | 14 (22) | 22 (34) |  | 25 (39) | 17 (26) |  | 25 (39) | 15 (23) |  | 14 (22) | 22 (34) |
| Stage, n (%) |  |  |  |  |  |  |  |  |  |  |  |  |  |  |  |
| I |  | 20 (26) | 18 (29) |  | 14 (22) | 22 (34) |  | 17 (26) | 15 (23) |  | 22 (34) | 12 (19) |  | 20 (31) | 17 (26) |
| II |  | 24 (31) | 18 (29) |  | 23 (35) | 20 (31) |  | 21 (32) | 18 (28) |  | 20 (31) | 24 (37) |  | 23 (35) | 23 (35) |
| III |  | 29 (38) | 24 (39) |  | 25 (39) | 20 (31) |  | 25 (39) | 29 (45) |  | 21 (32) | 27 (42) |  | 19 (29) | 24 (37) |
| IV |  | 4 (5) | 2 (3) |  | 3 (5) | 3 (5) |  | 2 (3) | 3 (5) |  | 2 (3) | 2 (3) |  | 3 (5) | 1 (2) |
| Tumor site^1^, n (%) |  |  |  |  |  |  |  |  |  |  |  |  |  |  |  |
| Colon – proximal |  | 23 (30) | 15 (24) |  | 17 (26) | 14 (22) |  | 21 (32) | 14 (22) |  | 21 (32) | 22 (34) |  | 21 (32) | 16 (25) |
| Colon – distal |  | 26 (34) | 25 (40) |  | 20 (31) | 30 (46) |  | 21 (32) | 24 (37) |  | 25 (39) | 22 (34) |  | 20 (31) | 28 (43) |
| Rectal |  | 28 (26) | 22 (36) |  | 28 (43) | 21 (32) |  | 23 (35) | 27 (42) |  | 19 (29) | 21 (32) |  | 24 (37) | 21 (32) |
| Treatment, n (%) |  |  |  |  |  |  |  |  |  |  |  |  |  |  |  |
| Surgery |  | 76 (99) | 62 (100) |  | 64 (99) | 65 (100) |  | 64 (99) | 65 (100) |  | 64 (99) | 65 (100) |  | 64 (99) | 65 (100) |
| Neo-adjuvant treatment |  | 24 (31) | 19 (31) |  | 25 (39) | 18 (28) |  | 20 (31) | 23 (35) |  | 17 (26) | 20 (31) |  | 21 (32) | 20 (31) |
| Total energy intake (kcal/d) |  | 1953 ± 573 | 1777 ± 547 |  | 1764 ± 531 | 1947 ± 530 |  | 1593 ± 481 | 2061 ± 602 |  | 1894 ± 570 | 1749 ± 540 |  | 1970 ± 612 | 1803 ± 528 |
| Total WCRF dietary score^2^ |  | 1.4 ± 0.3 | 2.8 ± 0.4 |  | 1.7 ± 0.6 | 2.4 ± 0.6 |  | 2.4 ± 0.7 | 1.7 ± 0.5 |  | 2.0 ± 0.7 | 2.1 ± 0.5 |  | 1.8 ± 0.6 | 2.4 ± 0.7 |
| Total DHD15-index^3^ |  | 66.0 ± 11.6 | 80.7 ± 14.8 |  | 58.6 ± 6.6 | 89.2 ± 8.5 |  | 74.9 ± 12.8 | 71.2 ± 14.6 |  | 73.6 ± 13.8 | 73.4 ± 13.9 |  | 64.6 ± 11.1 | 84.0 ± 12.0 |

Numbers are presented as mean ± SD or median (IQR) unless mentioned otherwise;
^1^Proximal consisting of: hepatic flexure, transverse colon, cecum, appendix, ascending colon; Distal consisting of: descending colon, sigmoid colon, splenic flexure; Rectal consisting of: rectosigmoid junction, rectum
^2^Adherence to the dietary recommendations of the World Cancer Research Fund (WCRF), ranged 0 to 5 [18];
^3^Adherence to the Dutch Healthy Diet guidelines 2015 (DHD15), ranged 0 to 130 [19].
